# Novel lactate dehydrogenase inhibitors with *in vivo* efficacy against *Cryptosporidium parvum*

**DOI:** 10.1371/journal.ppat.1007953

**Published:** 2019-07-29

**Authors:** Kun Li, Sara M. Nader, Xuejin Zhang, Benjamin C. Ray, Chi Yong Kim, Aditi Das, William H. Witola

**Affiliations:** 1 Department of Pathobiology, College of Veterinary Medicine, University of Illinois at Urbana-Champaign, Urbana, Illinois, United States of America; 2 Department of Chemistry, University of Illinois at Urbana-Champaign, Urbana, Illinois, United States of America; 3 Department of Comparative Biosciences, College of Veterinary Medicine, University of Illinois at Urbana-Champaign, Urbana, Illinois, United States of America; University of Wisconsin Medical School, UNITED STATES

## Abstract

*Cryptosporidium parvum* is a highly prevalent zoonotic and anthroponotic protozoan parasite that causes a diarrheal syndrome in children and neonatal livestock, culminating in growth retardation and mortalities. Despite the high prevalence of *C*. *parvum*, there are no fully effective and safe drugs for treating infections, and there is no vaccine. We have previously reported that the bacterial-like *C*. *parvum* lactate dehydrogenase (CpLDH) enzyme is essential for survival, virulence and growth of *C*. *parvum in vitro* and *in vivo*. In the present study, we screened compound libraries and identified inhibitors against the enzymatic activity of recombinant CpLDH protein *in vitro*. We tested the inhibitors for anti-*Cryptosporidium* effect using *in vitro* infection assays of HCT-8 cells monolayers and identified compounds NSC158011 and NSC10447 that inhibited the proliferation of intracellular *C*. *parvum in vitro*, with IC_50_ values of 14.88 and 72.65 μM, respectively. At doses tolerable in mice, we found that both NSC158011 and NSC10447 consistently significantly reduced the shedding of *C*. *parvum* oocysts in infected immunocompromised mice’s feces, and prevented intestinal villous atrophy as well as mucosal erosion due to *C*. *parvum*. Together, our findings have unveiled promising anti-*Cryptosporidium* drug candidates that can be explored further for the development of the much needed novel therapeutic agents against *C*. *parvum* infections.

## Author summary

*Cryptosporidium parvum* is a protozoan parasite that can cause a life-threatening gastrointestinal disease in children and in immunocompromised adults. The only approved drug for treatment of *Cryptosporidium* infections in humans is nitazoxanide, but it is not effective in immunocompromised individuals or in children with malnutrition. *C*. *parvum* possesses a unique lactate dehydrogenase (CpLDH) enzyme that it uses for generating metabolic energy (ATP) via the glycolytic pathway to fuel its growth and proliferation in the host. We have identified novel inhibitors for the enzymatic activity of CpLDH. Further, we have demonstrated that two of the CpLDH inhibitors effectively block the growth, proliferation and pathogenicity of *C*. *parvum* at tolerable doses in immunocompromised mice. Together, our findings have unveiled novel CpLDH inhibitors that can be explored for the development of efficacious therapeutic drugs against *C*. *parvum* infections.

## Introduction

The zoonotic and anthroponotic protozoan parasite, *Cryptosporidium parvum*, is a major cause of diarrheal diseases in children under the age of two, resulting in significant morbidity and mortality in poor-resource areas of developing countries [1]. In livestock, particularly in calves, lambs and goat kids, it causes a serious diarrheal syndrome, culminating in growth retardation and high neonatal mortalities [2–4]. *C*. *parvum* is highly prevalent because of its enormous capacity to reproduce in infected livestock, resulting in large amounts of infective parasite oocysts being shed in animal feces, and contaminating water sources as well as the general environment. The parasite oocysts in the environment are difficult to eliminate because of their resistance to virtually all kinds of chemical disinfectants, as well as to commonly used water treatments such as chlorination [5]. The efficacy of the only FDA-approved anti-*Cryptosporidium* drug in humans, nitazoxanide, is modest. Of particular concern, nitazoxanide is ineffective in those individuals most at risk for morbidity and mortality due to *Cryptosporidium* infections, including malnourished children and immunocompromised individuals [6]. There is currently no vaccine against *Cryptosporidium* infections.

Efforts to develop fully effective drugs against *Cryptosporidium* have largely been hampered by the lack of genetic tools for functional interrogation and validation of potential molecular drug targets in the parasite. Recently, however, a CRISPR/Cas9 gene manipulation approach [7], and a morpholino-based targeted gene knockdown approach [8, 9] in *C*. *parvum* have been developed. The completed and annotated genome sequence of *Cryptosporidium* indicates that, while the parasite lacks genes for conventional molecular drug targets found in other important protozoan parasites, it has several genes encoding plant-like and bacterial-like enzymes that catalyze potentially essential biosynthetic and metabolic pathways in *Cryptosporidium* [10]. Using a morpholino-based approach for targeted gene knockdown in *C*. *parvum*, we have previously validated that the *C*. *parvum* lactate dehydrogenase gene (CpLDH) that encodes a bacterial-like enzyme, is essential for survival, virulence and reproduction of *C*. *parvum* both *in vitro* and *in vivo* [8, 9].

In the present study, we screened compound libraries and identified compounds with inhibitory effect against the enzymatic activity of recombinant CpLDH protein *in vitro*. Among the identified CpLDH inhibitors, we have demonstrated that two of the inhibitors can effectively block the growth, proliferation and pathogenicity of *C*. *parvum in vivo* at tolerable doses, suggesting that they are potential candidates for development of drugs against *C*. *parvum* infections.

## Results

### Enzymatic activity of recombinant CpLDH protein

**Fig 1:**
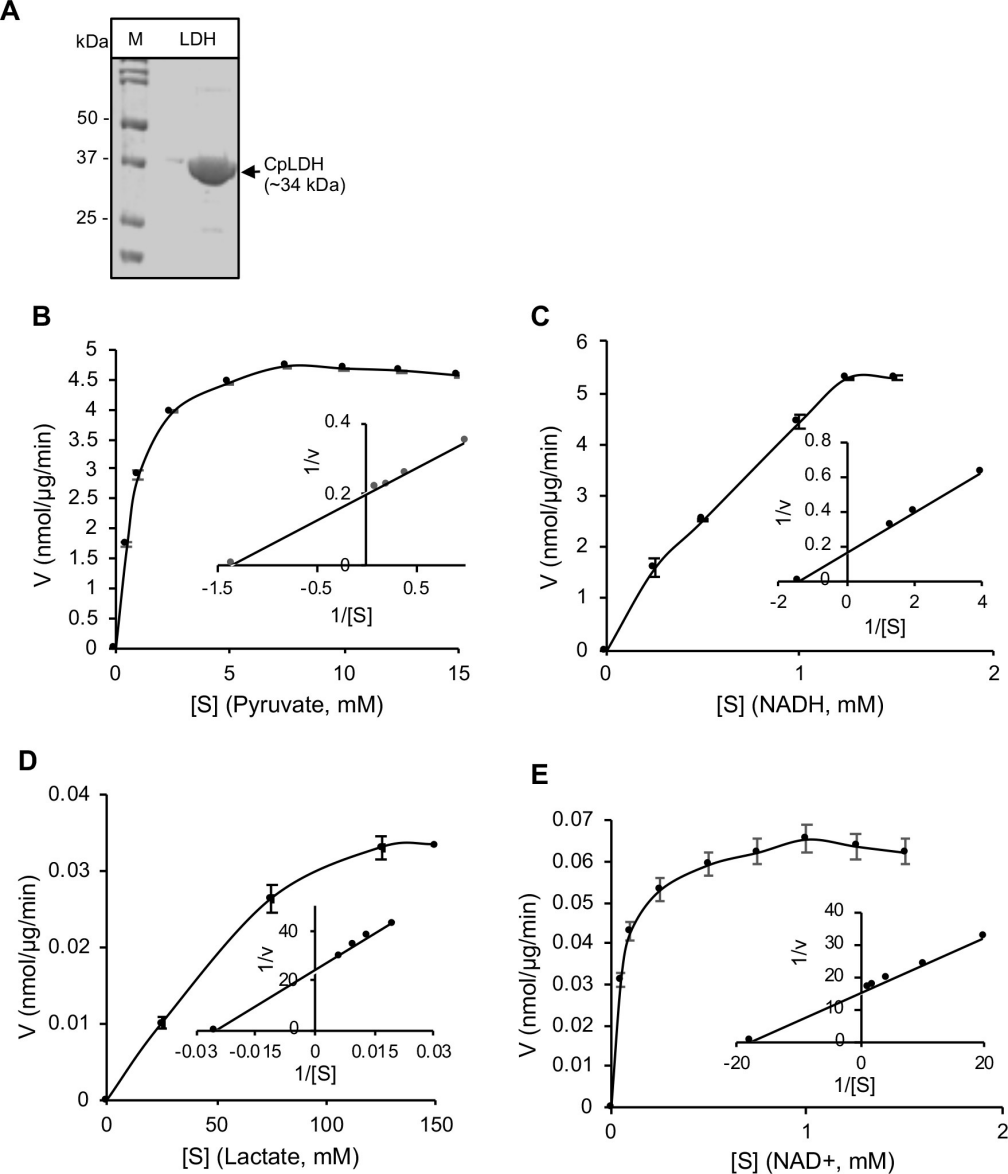
Analysis of the enzymatic activity of recombinant CpLDH protein. (A) SDS-PAGE analysis of the nickel affinity column chromatography-purified His-tagged recombinant CpLDH protein stained with coomassie blue (Lane M: protein ladder; Lane LDH: CpLDH protein). (B and C) Enzyme kinetics of recombinant CpLDH protein for the reduction of pyruvate to lactate on pyruvate as substrate (B), and NADH as co-factor (C). (D and E) Enzyme kinetics of recombinant CpLDH protein for the oxidation of lactate to pyruvate on lactate as substrate (D), and NAD+ as co-factor (E). Insets are Lineweaver–Burk representations of the saturation curves. S, substrate; V, velocity of the reaction. The data shown represent means of three independent experiments.

By sequencing, the cloned open reading frame of CpLDH gene was verified to be 966 bp long, and 99.79% identical to that reported in the genome database (GenBank accession number AF274310.1). It coded for a 321 amino acids long protein with amino acid residue substitutions of F-198-L, R-251-K and K-295-E when compared to that in GenBank. The expressed and purified His-tagged CpLDH protein was of the expected molecular size of about 34 kDa (Fig 1A). By analyzing the *in vitro* catalytic activities of recombinant CpLDH, we found that it depicted more activity in catalyzing the reduction of pyruvate to lactate than the oxidation of lactate to pyruvate. We found that recombinant CpLDH enzymatic catalytic activity was consistent with the Michaelis-Menten kinetics on pyruvate, NADH, lactate and NAD^+^ (Fig 1B–1E). The Lineweaver–Burk representation of the saturation curves (insets in Fig 1B–1E) showed that the *Km* of recombinant CpLDH for pyruvate was at least 54-fold lower than that for lactate, while its *Vmax* for pyruvate was 123-fold higher than that for lactate (Table 1). Our obtained enzymatic kinetic parameters for recombinant CpLDH in comparison to those reported previously for *C*. *parvum* CpLDH [11] are summarized in Table 1.

**Table 1:** CpLDH enzyme kinetics on substrates and co-factors.

Parameter	Pyruvate	NADH	Lactate	NAD^+^
*K*_*m*_ (μM)	731 (427)*	734 (92)*	39,625 (10,830)*	56 (62)*
*V*_*max*_ (nmol^-1^·μg^-1^·min^-1^)	5.04 (12.89)*	6.29 (9.33)*	0.04 (0.53)*	0.07 (0.56)*
*K*_*cat*_ (s^-1^)	8.42 (16.10)*	10.50 (11.70)*	0.07 (0.66)*	0.11 (0.70)*
*K*_*cat*_*/Km* (s^-1^·M^-1^)	1.15 x 10^4^ (3.77 x 10^4^)*	1.43 x 10^4^ (1.27 x 10^5^)*	1.73 (61.2)*	1.97 x 10^3^ (1.13 x 10^4^)*

*Reported by Zhang et al., 2015 [11].

### *In vitro* identification of inhibitors for recombinant CpLDH enzyme

**Fig 2:**
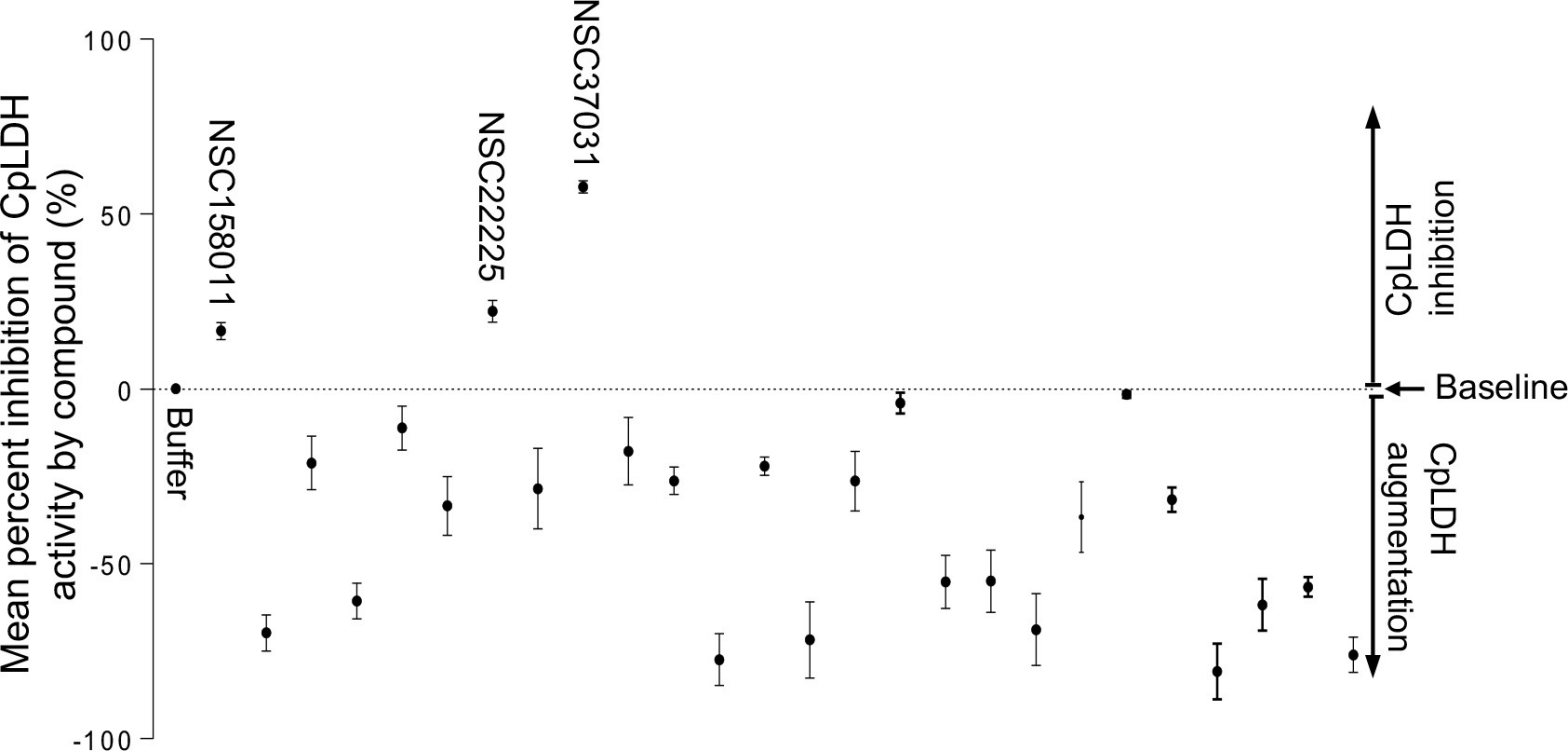
Effect of the diverse set compounds on the catalytic activity of recombinant CpLDH protein. Individually reconstituted compounds were used at a final concentration of 20 μM in the reaction for the reduction of pyruvate to lactate with recombinant CpLDH protein as enzyme. The mean percent inhibition of CpLDH activity by each compound was derived by dividing the mean change in optical density (ΔOD_340_) of the reaction after 2 min in the presence of the compound by the mean ΔOD_340_ of the reaction without compound, and multiplying the product by 100. The baseline mean percent inhibition of 0 (buffer) was for the reaction without compound, but with an equivalent volume of solvent used to reconstitute compound. Compounds with mean percent inhibition values greater than 0 were designated as inhibitors of the activity of CpLDH, while those with mean percent inhibition values less than 0 were classified as augmenters. Each reaction was performed in triplicate, and the data shown represents means of three independent experiments.

**Fig 3:**
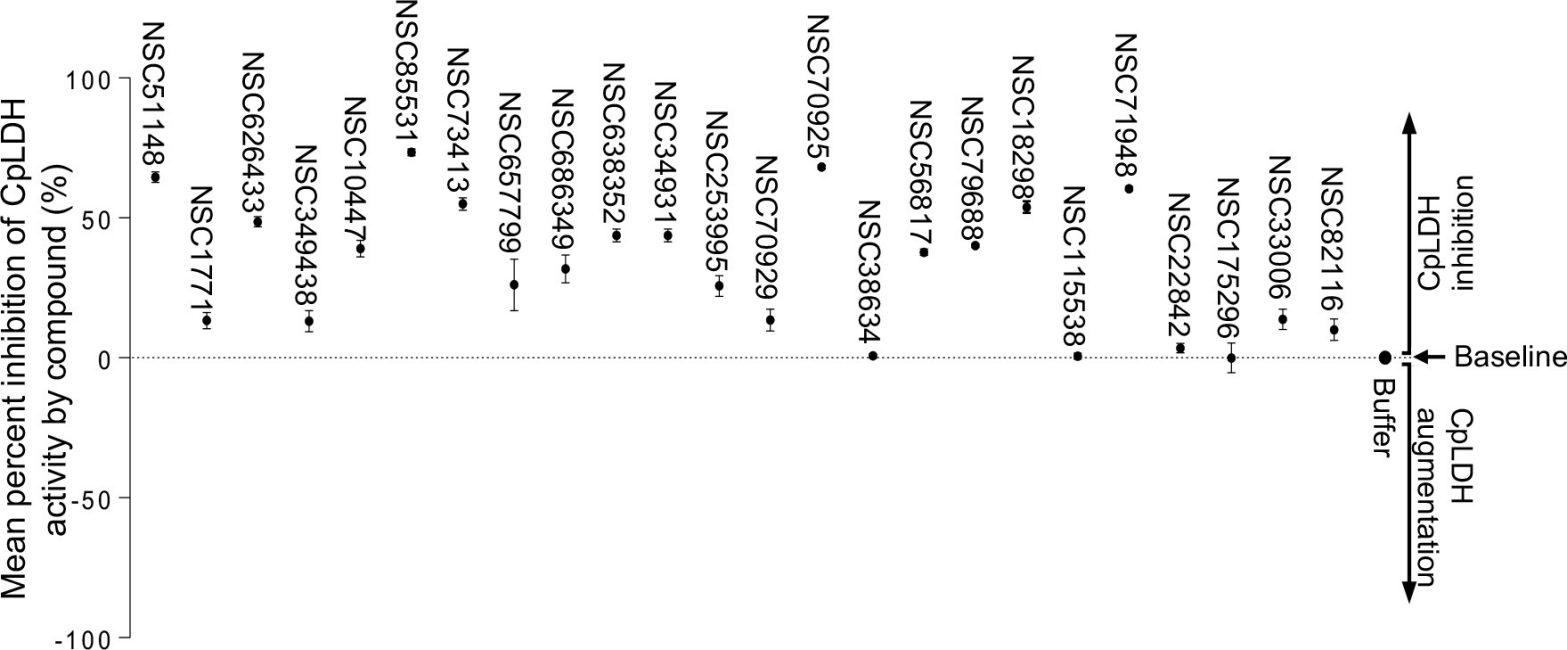
Effect of the Mechanistic Set IV compounds on the catalytic activity of recombinant CpLDH protein. Individually reconstituted compounds were used at a final concentration of 20 μM in the reaction for the reduction of pyruvate to lactate with recombinant CpLDH protein as enzyme. The mean percent inhibition of CpLDH activity by each compound was derived by dividing the mean change in optical density (ΔOD_340_) of the reaction after 2 min in the presence of the compound by the mean ΔOD_340_ of the reaction without compound, and multiplying the product by 100. The baseline mean percent inhibition of 0 (buffer) was for the reaction without compound, but with an equivalent volume of solvent used to reconstitute compound. Compounds with mean percent inhibition values greater than 0 were designated as inhibitors of the activity of CpLDH, while those with mean percent inhibition values less than 0 were classified as augmenters (data points below 0 that are not shown in Fig 3 were 765 in total, and are listed in S4 Table together with their corresponding CpLDH percent inhibition values). Each reaction was performed in triplicate, and the data shown represents means of three independent experiments.

We found that recombinant CpLDH had more catalytic activity for the reduction of pyruvate to lactate than for the oxidation of lactate to pyruvate. Therefore, we used the assay for reduction of pyruvate to lactate to screen chemical compounds for inhibitors of the enzymatic activity of recombinant CpLDH *in vitro*. Within the group of the 27 diverse chemical compounds (S1 Table) [12], we identified three compounds (NSC22225, NSC37031 and NSC158011) that significantly (*P* < 0.05) inhibited the catalytic activity of recombinant CpLDH for the reduction of pyruvate to lactate (Fig 2). On the other hand, among the 800 compounds in the Mechanistic Set IV (S2 Table), we found 20 that had significant (*P* < 0.05) inhibitory effect on the catalytic activity of recombinant CpLDH (Fig 3). Those 20 compounds included: NSC51148, NSC1771, NSC626433, NSC349438, NSC10447, NSC85561, NSC73413, NSC657799, NSC686349, NSC638352, NSC34931, NSC253995, NSC70929, NSC70925, NSC56817, NSC79688, NSC18298, NSC71948, NSC22842, NSC33006, and NSC82116. The rest of the compounds either had no effect or augmented the catalytic activity of recombinant CpLDH and were thus not pursued further.

### Molecular docking simulations of NSC158011 and NSC10447 binding to CpLDH and human LDH

To decipher the differences in the interactions of NSC150811 and NSC10447 (S1 Fig) with CpLDH and human LDH proteins, an *in silico* molecular docking using Autodock Vina [13] was performed to determine the most energetically favorable poses of the compounds complexed with the rigid structures of both CpLDH and human LDH. Both compounds were found to bind most favorably into the co-factor-binding pocket of the CpLDH and human LDH, where NADH binds to reduce pyruvate to lactate.

NSC158011 complexed with CpLDH with an affinity of -6.4 kcal/mol. (Table 2). The ligand was surrounded in hydrophobic and hydrophilic interactions. Hydrophobic interactions occurred with the Ile-100, Ala-80, and Ile-15 residues and the nonpolar aromatic rings of the molecule, while polar interactions occurred with the Asn-97 and Gln-14 residues and the highly polar thio-amide group (S2 Fig). Although secondary amines do not possess a strong dipole moment, it is possible that the positively-charged Arg-85 residue interacts with the resonance-stabilized deprotonated thio-amide moiety of NSC158011. The docked NSC158011 possessed little solvent exposure, likely due to its folded nature and position, tight within the protein pocket (S2 Fig). NSC158011 complexed with human LDH with an affinity of -7.2 kcal/mol. (Table 2). The hydrophobic Phe-119, Ile-120, Val-116, Val-98, Ala-96, Val-26, and Val-28 residues interacted with the non-polar aromatic rings of the NSC158011 (S3 Fig). Remarkably, these same non-polar aromatic rings are also heavily solvent-exposed (S3 Fig).

**Table 2:** Affinity of the most favorable compound poses within lactate dehydrogenase active site.

Lactate Dehydrogenase	Ligand	Affinity (kcal/mol)	Grid Box size (Å)	Exhaustiveness
4ND2 (CpLDH)	NSC10447	-7.6	40*40*40	10
1I0Z (human LDH)	NSC10447	-7.1	24*14*20	10
4ND2 (CpLDH)	NSC158011	-6.4	40*40*40	10
1I0Z (human LDH)	NSC158011	-7.2	24*14*20	10

NSC10447 complexed with CpLDH with an affinity of -7.6 kcal/mol. (Table 2). The ligand was involved primarily in polar interactions with the surrounding Ser81, Thr-79, Thr-229, Thr-231, and positive-charged Arg-85 residues, mediated through interactions with semi-polar carbonyl carbons abundant on one side of the molecule (S4 Fig). The non-polar side of the molecule interacted favorably with the hydrophobic Tyr-233 residue internal to the protein (S4 Fig). There was a weak hydrogen-bonding interaction between the backbone of Asn-97 and an alcohol group on the ligand. Additionally, the hydrophilic carbons had weak but notable solvent exposure (S4 Fig). NSC10447 complexed with human LDH with an affinity of -7.1 kcal/mol. (Table 2). The ligand was involved primarily in hydrophobic interactions with the surrounding Val-98, Ala-96, Val-94, Phe119, Val-26, Tyr-83, and Val-116 residues that stabilized the non-polar moiety of the molecule (S5 Fig). Interestingly, there was a weak, polar, anti-bonding interaction with the Thr-95 residue. The polar alcohol groups and the attached carbons were heavily solvent exposed in the final docking conformation (S5 Fig).

The results of the molecular docking simulation showed that due to the high level of interaction between the two lead compounds and the residues within the LDH cofactor-binding pocket, NSC158011 and NSC10447 each bound favorably to both CpLDH and human LDH proteins. It can be proposed that the compounds act as competitive inhibitors for the LDH enzyme, binding favorably to the hydrophobic residues internal to the co-factor-binding pocket and blocking the enzyme from binding NADH, thus preventing the hydride transfer that powers the conversion of pyruvate to lactate.

### NSC158011 and NSC10447 inhibit *C*. *parvum* growth *in vitro*

**Fig 4:**
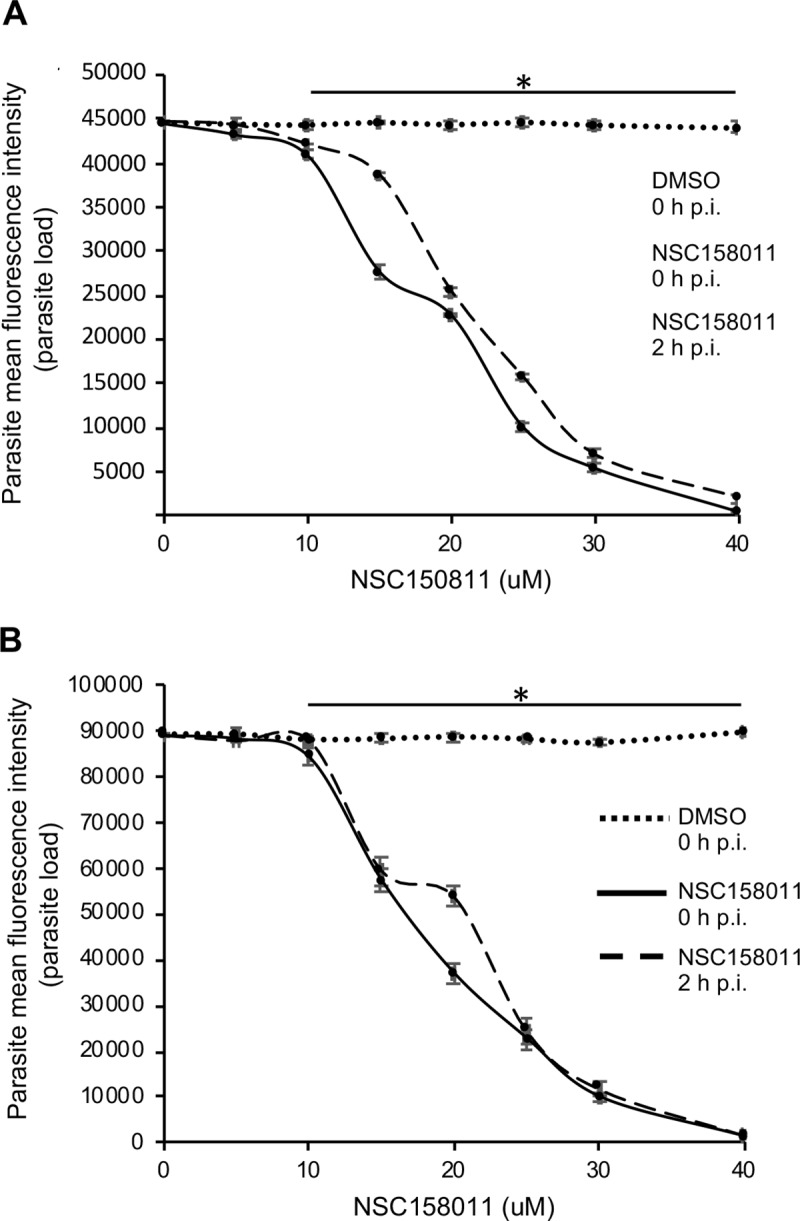
Analysis of the effect of varying concentrations of compound NSC158011 on the growth of *Cryptosporidium parvum* in HCT-8 cells. Equal amounts of freshly excysted sporozoites of *C*. *parvum* were inoculated into HCT-8 cells in culture and varying concentrations of NSC158011 added at the time of infection (solid line) or added 2 h post-infection (p.i.) (dashed line). Control infected cells (dotted line) were treated immediately p.i. with volumes of DMSO equivalent to those used in the compound-treated cultures. The cells were analyzed for parasite infectivity and proliferation by an immunofluorescence assay after (A) 48 h, and (B) 72 h of culture. The fluorescence generated by intracellular *C*. *parvum* merozoites was quantified and is shown on the *Y*-axis representing the parasite load. The data shown represent means of three independent experiments with standard error bars and levels of statistical significance between groups indicated by asterisk (*, *P* < 0.05).

All the compounds that we found to have inhibitory effect against recombinant CpLDH activity were first analyzed for *in vitro* cytotoxicity in a mammalian cell line, HCT-8 (American Type Culture Collection Item number: CCL244) before testing their anti-*Cryptosporidium* efficacy. For cytotoxicity screening, varying concentrations of each compound (from 0 to 700 μM) were tested in triplicate using the WST-1 cell proliferation assay and the half maximal inhibitory concentration (cytotoxicity IC_50_ values) of the compounds in HCT-8 cells (S3 Table) were derived from dose–response curves using GraphPad PRISM software. To test the compounds’ efficacy against *C*. *parvum in vitro*, an initial screen was performed using concentrations that were at least 50% lower than the compounds’ respective cytotoxicity IC_50_ values (S3 Table). NSC10447 and NSC158011 from the diverse group and Mechanistic Set IV group, respectively, were found to significantly (*P* < 0.05) inhibit *C*. *parvum* proliferation *in vitro* at 48 h post-infection. Therefore, these two compounds were selected for secondary analysis of anti-*Cryptosporidium* efficacy using varying concentrations and durations of culture to derive the IC_50_ values for the inhibition of parasite proliferation. For each compound, the assays were done in two formats: (1) by adding the compound to the HCT-8 cells culture shortly before infecting them with *C*. *parvum* sporozoites, with the goal to assess whether the compounds would block host cell invasion by sporozoites, and (2) by adding the compounds to the cells 2 h post-infection to determine the effect of the compounds on intracellular parasites. When the cultures were analyzed at 48 h post-infection, compound NSC158011 was found to have a significant (*P* < 0.05) concentration-dependent effect of inhibiting proliferation of intracellular *C*. *parvum* merozoites in HCT-8 cells starting at 10 μM (with 40 μM blocking parasite growth almost completely) relative to the control infected cultures without compound treatment (Fig 4A). Treating the cultures with NSC158011 compound 2 h post-infection also resulted in a concentration-dependent reduction in parasite proliferation, but with a slight decrease in compound efficacy relative to treating at the time of infection (Fig 4A). By using GraphPad PRISM software, the half maximal inhibitory concentration (IC_50_) values of NSC158011 for *C*. *parvum in vitro* were derived from the dose–response curves. The NSC158011 IC_50_ values at 48 h post-infection for inhibition of *C*. *parvum* growth when compound was added immediately, or 2 h after infecting the HCT-8 cells were 14.88 and 15.81 μM, respectively. Analysis of the inhibitory effect of NSC158011 on parasite proliferation at 72 h post-infection, depicted similar concentration-dependent effects (Fig 4B), with IC_50_ values of 15.63 and 16.50 μM when compound was added immediately or 2 h post-infection, respectively. After infecting HCT-8 cells, *C*. *parvum* is able to proliferate for 3–4 days before becoming growth-arrested. Therefore, during both observation time-points of 48 h and 72 h post-infection, *C*. *parvum* if untreated was expected to be in proliferative phase. Consistently, in the untreated infected cells, the relative parasite load at 72 h post-infection was about 2-fold that observed at 48 h post-infection (Fig 4A and 4B).

**Fig 5:**
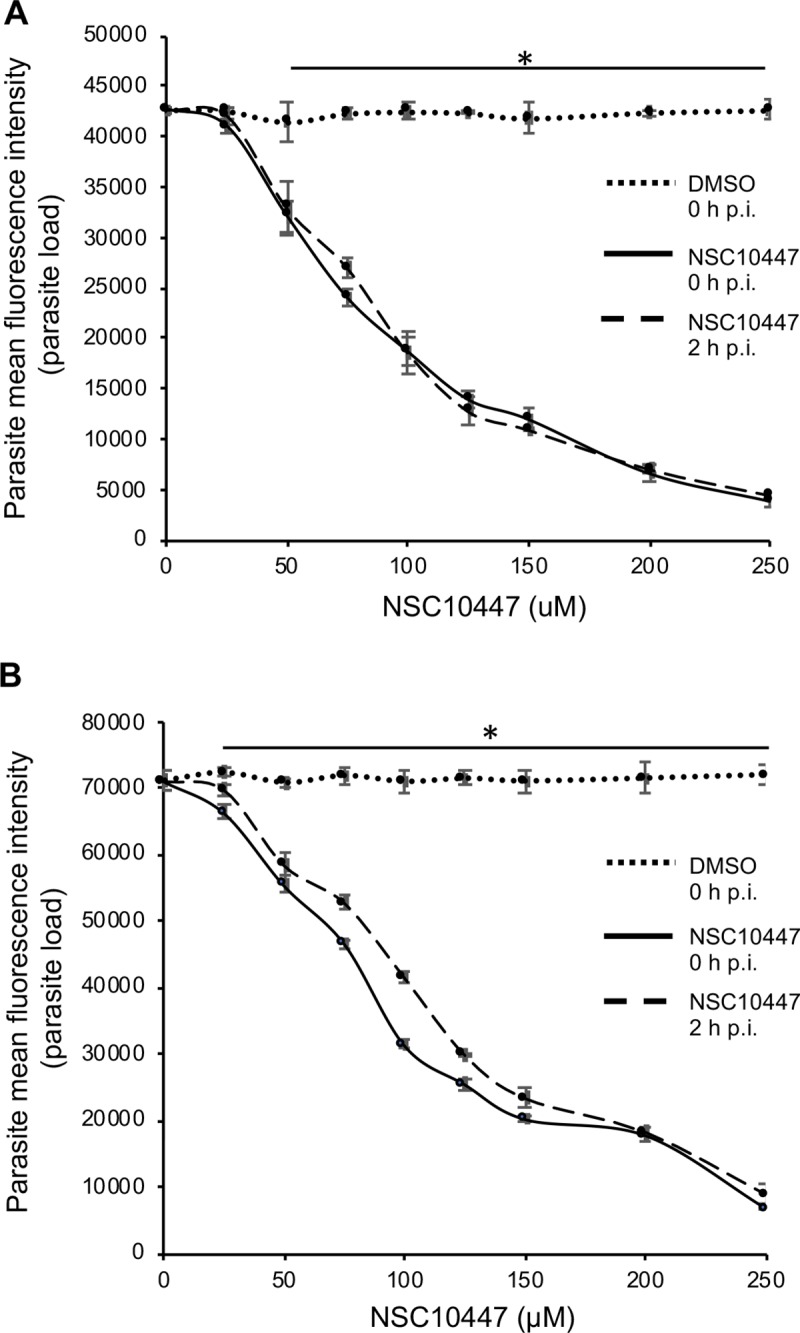
Analysis of the effect of varying concentrations of compound NSC10447 on the growth of *Cryptosporidium parvum* in HCT-8 cells. Equal amounts of freshly excysted sporozoites of *C*. *parvum* were inoculated into HCT-8 cells in culture and varying concentrations of NSC10447 added at the time of infection (solid line) or added 2 h post-infection (p.i.) (dashed line). Control infected cells (dotted line) were treated immediately p.i. with volumes of DMSO equivalent to those used in the compound-treated cultures. The cultures were analyzed for parasite infectivity and proliferation by an immunofluorescence assay after (A) 48 h, and (B) 72 h of culture. The fluorescence generated by intracellular *C*. *parvum* merozoites was quantified and is shown on the *Y*-axis representing the parasite load. The data shown represent means of three independent experiments with standard error bars and levels of statistical significance between groups indicated by asterisk (*, *P* < 0.05).

Compound NSC10447 also depicted a concentration-dependent inhibitory effect on parasite growth, both at 48 h (Fig 5A) and 72 h (Fig 5B) time points of observation. The efficacy of NSC10447 when added immediately or 2 h after infecting the cells was similar (Fig 5A). The NSC10447 IC_50_ values at 48 h post-infection for inhibition of *C*. *parvum* growth when compound was added immediately, and 2 h after infecting the HCT-8 cells were 72.65 and 79.52 μM, respectively. Consistently, NSC10447 had a concentration-dependent inhibitory effect on parasite growth at 72 h time point of observation (Fig 5B), with IC_50_ values of 83.63 and 95.17 μM, when the compound was added immediately, and 2 h after infecting the HCT-8 cells, respectively. Noteworthy, in all instances, NSC158011 depicted significantly higher *in vitro* efficacy against *C*. *parvum* than NSC10447 (Table 3). The IC_50_ values of NSC158011 and NSC10447 for the inhibition of the catalytic activity of recombinant CpLDH *in vitro* were 76.59 μM and 46.33 μM, respectively (Table 3).

**Table 3:** NSC158011 and NSC10447 IC_50_ values on CpLDH enzyme and *C*. *parvum*.

Parameter	NSC158011 (μM)	NSC10447 (μM)
Inhibition of recombinant CpLDH activity	76.59	46.33
Inhibition of *C*. *parvum* growth at 48 h p.i.	14.88	72.65
Inhibition of *C*. *parvum* growth at 48 h p.i. (2 h)*	15.81	79.52
Inhibition of *C*. *parvum* growth at 72 h p.i.	15.63	83.63
Inhibition of *C*. *parvum* growth at 72 h p.i. (2 h)*	16.50	95.17

*Compounds were added to culture 2 h post-infection (p.i.)

**Fig 6:**
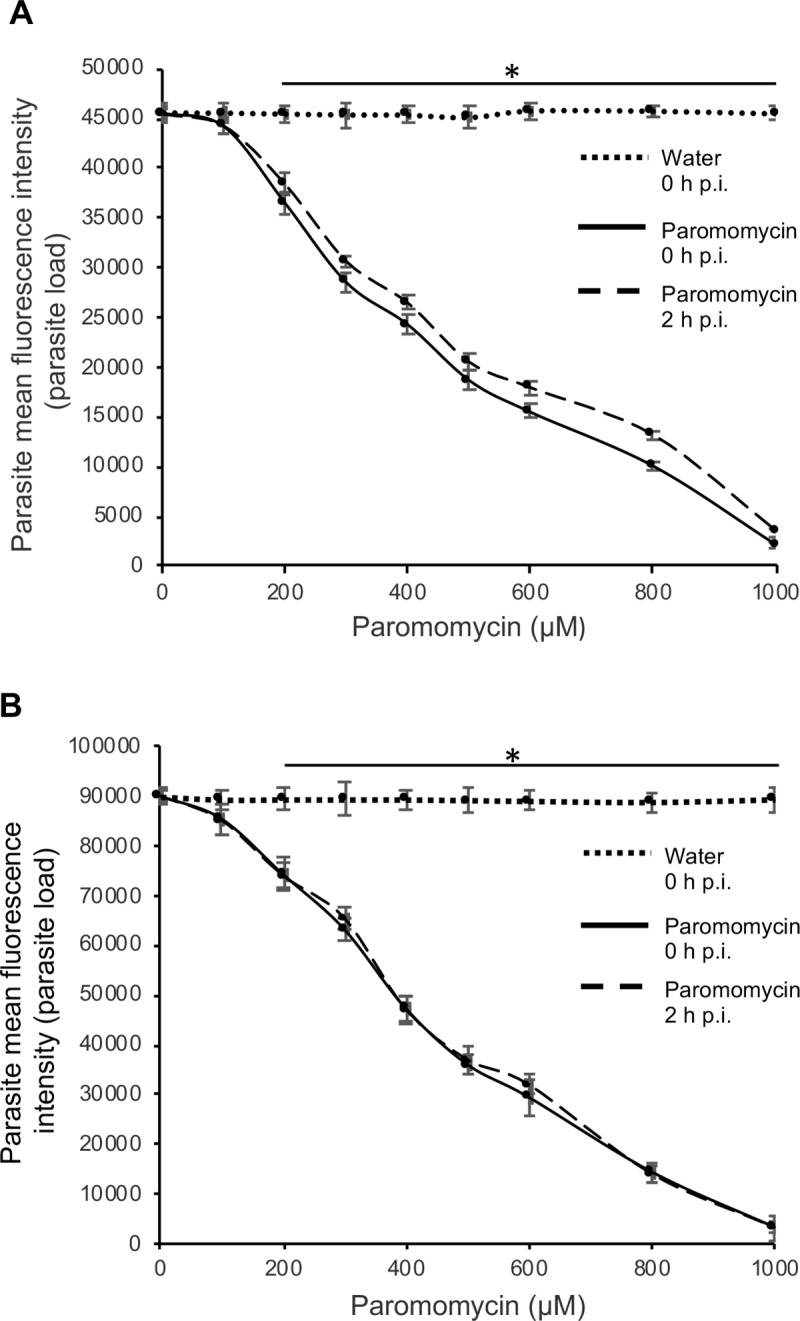
Analysis of the effect of varying concentrations of paromomycin on the growth of *Cryptosporidium parvum* in HCT-8 cells. Equal amounts of freshly excysted sporozoites of *C*. *parvum* were inoculated into HCT-8 cells in culture and varying concentrations of paromomycin dissolved in sterile distilled water was added at infection (solid line) or 2 h post-infection (p.i.) (dashed line). Control infected cells (dotted line) were treated immediately p.i. with volumes of sterile distilled water equivalent to those used in the paromomycin-treated cultures. The cultures were analyzed for parasite infectivity and proliferation by an immunofluorescence assay after (A) 48 h, and (B) 72 h of culture. The fluorescence generated by intracellular *C*. *parvum* merozoites was quantified and is shown on the *Y*-axis representing the parasite load. The data shown represent means of three independent experiments with standard error bars and levels of statistical significance between groups indicated by asterisk (*, *P* < 0.05).

We used paromomycin as the positive control treatment. Using *in vitro* assays the cytotoxicity of paromomycin in HCT-8 cells has been reported to be negligible even when used at concentrations above 1000 μM [14, 15]. Therefore, we tested the *in vitro* efficacy of paromomycin against *C*. *parvum* at varying concentrations up to a maximum of 1000 μM, and found it to have a concentration-dependent effect of inhibiting *C*. *parvum* growth *in vitro*, both at 48 h (Fig 6A) and 72 h (Fig 6B) post-infection, with IC_50_ values of 450 and 400 μM, respectively. Others have previously reported paromomycin to have an IC_50_ of 711 μM for inhibition of *C*. *parvum* growth in HCT-8 cells [15]. There was no notable significant difference in paromomycin inhibitory effect against *C*. *parvum* between starting the treatment immediately or 2 h after infection of the HCT-8 cells.

### NSC158011 and NSC10447 possess *in vivo* efficacy against *C*. *parvum*

Compound NSC158011 and NSC10447 that were found to inhibit *C*. *parvum* growth *in vitro* were selected for *in vivo* testing using a mouse infection model. Prior to use in mice, the highest tolerable doses in mice for the two compounds were found to be 400 mg/kg and 1000 mg/kg for NSC158011 and NSC10447, respectively. These doses consistently did not induce any toxicity signs (changes from normal physical activity, respiration, body temperature, feeding pattern, body posture, fur condition or occurrence of death) over 7 days of daily oral gavage in mice. In the case of NSC158011, the dose of 400 mg/kg intraperitoneal administration in mice has also been previously shown not to be toxic to mice [16]. Thus, the doses of 400 mg/kg and 1000 mg/kg were selected as the highest doses for testing the efficacy of NSC158011 and NSC10447, respectively, against *C*. *parvum* growth and proliferation in mice. Paromomycin at 100 mg/kg daily by oral gavage was used as a positive control. The daily load of *C*. *parvum* oocysts shed in mice feces was determined using real time PCR quantification of the *C*. *parvum* 18s rRNA gene. As expected, in the feces of untreated infected mice, *C*. *parvum* genomic DNA was almost undetectable during the first 2 days post-infection, but was detectable from 3 days post-infection, and increased progressively with increase in the number of days post-infection (Tables 4–6). The notable lower *C*. *parvum* DNA in the feces of the untreated infected mice in Table 6 when compared to those for Tables 4 and 5 is because the infection assays for Tables 3 and 4 were done using freshly purified oocysts, while those for Table 6 were done using oocysts that were purified 3 months earlier, and thus their infectivity could have been lower. We found that NSC158011 at 400 mg/kg significantly (*P* < 0.05) reduced shedding of *C*. *parvum* oocysts in mice feces, comparable to the efficacy of paromomycin (Table 4).

**Table 4:** Real-time PCR quantification of *C*. *parvum* DNA in fecal samples of treated or untreated infected mice.

DPI*	Infected untreated mice fecal *C*. *parvum* DNA load (ng/μL)	Infected NSC158011 (400 mg/kg) treated mice fecal *C*. *parvum* DNA load (ng/μL)	Infected paromomycin (100 mg/kg) treated mice fecal *C*. *parvum* DNA load (ng/μL)	Uninfected mice fecal *C*. *parvum* DNA load (ng/μL)
1	1.3 ± 0.8 (x10^-11^)	2.9 ± 1.7 (x10^-10^)	9.6 ± 4.4 (x10^-11^)	0.0
2	4.2 ± 0.2 (x10^-10^)	4.8 ± 0.3 (x10^-10^)	4.5 ± 3.1 (x10^-9^)	0.0
3	6.1 ± 0.1 (x10^-8^)	8.8 ± 0.8 (x10^-9^)	1.8 ± 0.9 (x10^-9^)	0.0
4	8.5 ± 0.5 (x10^-7^)	5.3 ± 0.4 (x10^-8^)	4.3 ± 0.1 (x10^-8^)	0.0
5	6.5 ± 1.2 (x10^-5^)	2.2 ± 0.08 (x10^-7^)	2.3 ± 0.8 (x10^-7^)	0.0
6	1.4 ± 0.3 (x10^-4^)	2.4 ± 0.1 (x10^-6^)	4.3 ± 0.1 (x10^-6^)	0.0
7	7.5 ± 1.3 (x10^-3^)	2.3 ± 0.1 (x10^-6^)	3.5 ± 0.5 (x10^-6^)	0.0
8	5.3 ± 0.9 (x10^-3^)	1.6 ± 0.1 (x10^-6^)	2.4 ± 0.1 (x10^-6^)	0.0

*Days post-infection with *C*. *parvum*

**Table 5:** Real-time PCR quantification of *C*. *parvum* DNA in fecal samples of treated or untreated infected mice.

DPI*	Infected untreated mice fecal *C*. *parvum* DNA load (ng/μL)	Infected NSC158011 (100 mg/kg) treated mice fecal *C*. *parvum* DNA load (ng/μL)	Infected NSC158011 (200 mg/kg) treated mice fecal *C*. *parvum* DNA load (ng/μL)	Infected paromomycin (100 mg/kg) treated mice fecal *C*. *parvum* DNA load (ng/μL)	Uninfected mice fecal *C*. *parvum* DNA load (ng/μL)
1	1.5 ± 0.2 (x10^-9^)	1.5 ± 0.2 (x10^-9^)	1.2 ± 0.2 (x10^-9^)	1.2 ± 0.3 (x10^-9^)	0.0
2	4.6 ± 0.1 (x10^-8^)	1.8 ± 0.4 (x10^-8^)	1.3 ± 0.4 (x10^-8^)	1.1 ± 0.3 (x10^-8^)	0.0
3	5.8 ± 0.2 (x10^-7^)	2.3 ± 0.4 (x10^-7^)	1.7 ± 0.2 (x10^-7^)	2.3 ± 0.2 (x10^-7^)	0.0
4	6.2 ± 0.2 (x10^-5^)	3.4 ± 0.1 (x10^-6^)	2.9 ± 0.1 (x10^-6^)	3.3 ± 0.1 (x10^-6^)	0.0
5	1.7 ± 0.1 (x10^-4^)	9.8 ± 0.3 (x10^-5^)	8.0 ± 0.3 (x10^-5^)	9.2 ± 0.3 (x10^-6^)	0.0
6	4.2 ± 0.1 (x10^-3^)	3.1 ± 0.2 (x10^-5^)	2.0 ± 0.1 (x10^-5^)	2.1 ± 0.1 (x10^-6^)	0.0
7	2.0 ± 0.3 (x10^-3^)	8.0 ± 0.1 (x10^-5^)	4.0 ± 0.1 (x10^-5^)	6.4 ± 0.1 (x10^-6^)	0.0
8	6.3 ± 0.4 (x10^-3^)	4.1 ± 0.1 (x10^-5^)	1.5 ± 0.1 (x10^-5^)	2.7 ± 0.1 (x10^-6^)	0.0

*Days post-infection with *C*. *parvum*

**Table 6:** Real-time PCR quantification of *C*. *parvum* DNA in fecal samples of treated or untreated infected mice.

DPI*	Infected untreated mice fecal *C*. *parvum* DNA load (ng/μL)	Infected NSC10447 (250 mg/kg) treated mice fecal *C*. *parvum* DNA load (ng/μL)	Infected NSC10447 (500 mg/kg) treated mice fecal *C*. *parvum* DNA load (ng/μL)	Infected NSC10447 (1000 mg/kg) treated mice fecal *C*. *parvum* DNA load (ng/μL)	Infected paromomycin (100 mg/kg) treated mice fecal *C*. *parvum* DNA load (ng/μL)	Uninfected mice fecal *C*. *parvum* DNA load (ng/μL)
1	4.0 ± 0.1 (x10^-10^)	4.4 ± 0.5 (x10^-10^)	1.0 ± 0.5 (x10^-10^)	1.2 ± 0.2 (x10^-9^)	3.1 ± 0.2 (x10^-10^)	0.0
2	6.1 ± 0.2 (x10^-9^)	7.3 ± 0.2 (x10^-10^)	9.4 ± 0.2 (x10^-10^)	3.0 ± 0.7 (x10^-9^)	6.6 ± 0.5 (x10^-9^)	0.0
3	2.5 ± 0.1 (x10^-8^)	1.0 ± 0.1 (x10^-8^)	6.7 ± 0.6 (x10^-9^)	6.4 ± 0.2 (x10^-9^)	5.2 ± 0.3 (x10^-9^)	0.0
4	2.5 ± 0.2 (x10^-7^)	1.2 ± 0.1 (x10^-7^)	1.4 ± 0.1 (x10^-7^)	1.1 ± 0.1 (x10^-7^)	4.7 ± 0.2 (x10^-8^)	0.0
5	3.1 ± 0.7 (x10^-6^)	1.2 ± 0.1 (x10^-6^)	1.6 ± 0.1 (x10^-6^)	1.3 ± 0.1 (x10^-6^)	5.0 ± 0.6 (x10^-7^)	0.0
6	1.2 ± 0.2 (x10^-5^)	4.8 ± 0.2 (x10^-6^)	5.9 ± 0.4 (x10^-6^)	5.6 ± 0.4 (x10^-6^)	4.8 ± 0.1 (x10^-6^)	0.0
7	2.7 ± 0.8 (x10^-4^)	1.9 ± 0.2 (x10^-5^)	9.3 ± 0.2 (x10^-6^)	6.3 ± 0.4 (x10^-6^)	5.1 ± 0.8 (x10^-6^)	0.0
8	1.7 ± 0.9 (x10^-3^)	4.7 ± 0.1 (x10^-5^)	3.0 ± 0.4 (x10^-5^)	1.2 ± 0.8 (x10^-6^)	5.4 ± 0.1 (x10^-6^)	0.0

*Days post-infection with *C*. *parvum*

As expected, *C*. *parvum* DNA was consistently undetectable at all time points sampled in the uninfected mice (Table 4). By day 7 post-infection, both NSC158011 and paromomycin treatment had reduced the shedding of *C*. *parvum* in mice feces by about 3000-fold when compared to the untreated infected mice samples (Table 4). This suggested that NSC158011, at 400 mg/kg, had sustained anti-*Cryptosporidium* efficacy *in vivo* comparable to that of 100 mg/kg of paromomycin. We titrated the dose of NSC158011 to determine the effect of lower dosages. We observed a dose-dependent reduction in efficacy of NSC158011, with 200 mg/kg having about 10-fold lower efficacy than paromomycin during the last three days of treatment. Consistently, 100 mg/kg of NSC158011 showed lower efficacy than 200 mg/kg NSC158011 (Table 5).

For testing the *in vivo* anti-*Cryptosporidium* efficacy of NSC10447, in addition to testing the highest tolerable dose of 1000 mg/kg, we also tested lower doses of 250 mg/kg and 500 mg/kg. While the *C*. *parvum* DNA was undetectable in the uninfected mice’s feces, the untreated infected mice had readily detectable *C*. *parvum* DNA by day 3 post-infection, that then increased progressively with increase in number of days post-infection (Table 6).

From day 3 until day 8 of treatment, compared to the infected untreated, mice treated with NSC10447 at 250, 500 and 1000 mg/kg showed sustained significantly lower (by at least 50%) *C*. *parvum* DNA load in their feces (Table 6). There was a notable dose-dependent effect, with 1000 mg/kg having the highest efficacy (Table 6). Notably, on day 7 and 8 post-infection, the 1000 mg/kg dose of NSC10447 maintained anti-*Cryptosporidium* efficacy that was comparable to that of paromomycin, while both 250 and 500 mg/kg doses depicted lower efficacies than paromomycin (Table 6).

**Fig 7:**
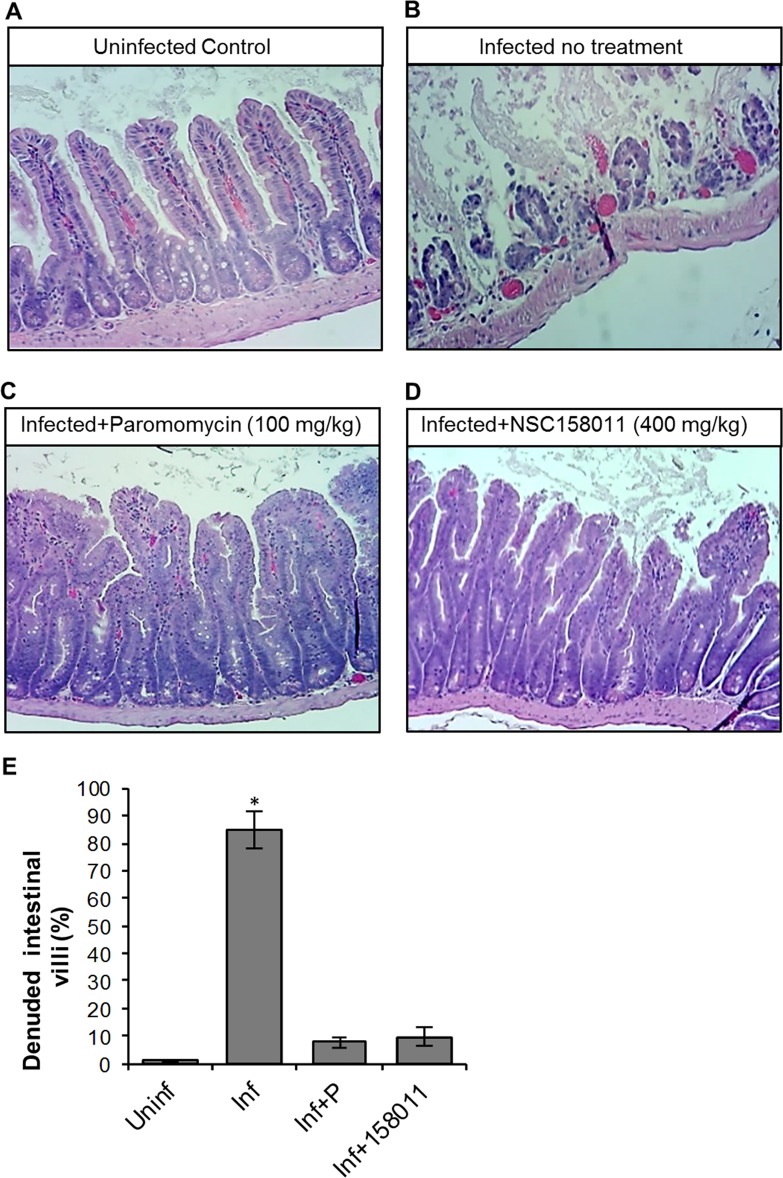
Histopathological analysis of the effect of *Cryptosporidium parvum* infection in the lower small intestines of mice with or without NSC158011 treatment. Mice infected with *C*. *parvum* were maintained untreated (Infected no treatment), treated with paromocycin (Infected+Paromomycin (100 mg/kg)) or treated with compound NSC158011 at 400 mg/kg (Infected+NSC158011 (400 mg/kg)) for 8 days. Uninfected mice were maintained as control (Uninfected Control). After 8 days, mice were sacrificed and the lower intestinal tissue processed for histology and stained with hematoxylin and eosin. (A) Uninfected control mice samples depicted intact intestinal epithelium with prominent villi. (B) In contrast, infected mice without treatment depicted denuded villi. Both (C) paromomycin and (D) NSC158011 treated infected mice depicted intact intestinal epithelium with prominent villi that were comparable to the uninfected control. The images are representative of samples analyzed from 3 mice per treatment group. (E) The mean percentage of denuded intestinal villi in 4 randomly chosen microscopic fields per sample from the uninfected (Uninf), infected untreated (Inf), Infected treated with paromomycin (Inf+P), and infected treated with 400 mg/kg NSC150811 (Inf+150811) mice. The data shown represent means for samples from three mice per group with standard error bars and levels of statistical significance depicted (*P < 0.05).

**Fig 8:**
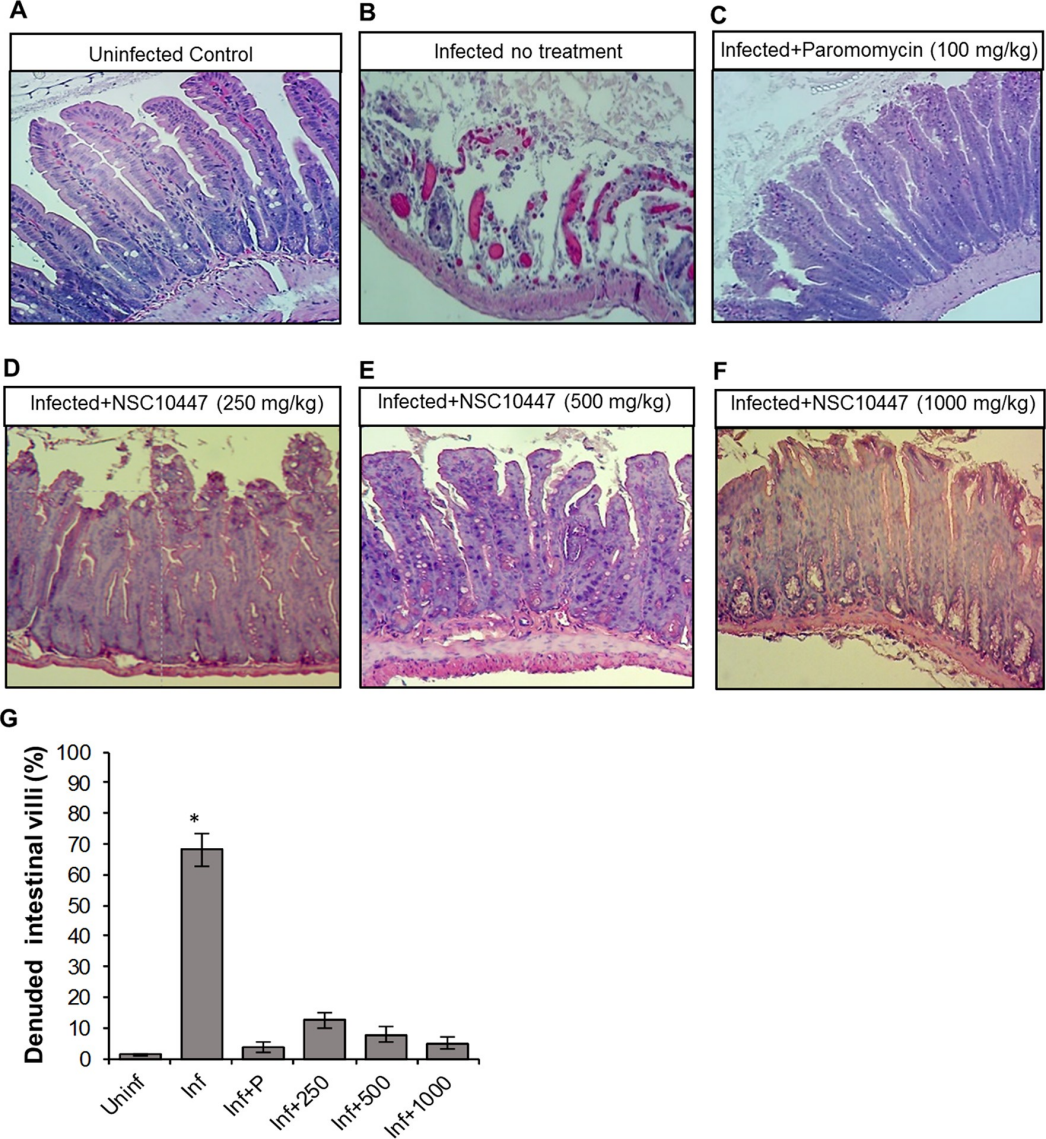
Histopathological analysis of the effect of *Cryptosporidium parvum* infection in the lower small intestines of mice with or without NSC10447 treatment. Mice infected with *C*. *parvum* were maintained untreated (Infected no treatment), treated with paromocycin at 100 mg/kg (Infected+Paromomycin (100 mg/kg)) or treated with compound NSC10447 (Infected+NSC10447) at varying doses (250, 500 or 1000 mg/kg) for 8 days. Uninfected mice were maintained as control (Uninfected Control). After 8 days, mice were sacrificed and the lower intestinal tissue processed for histology, and stained with hematoxylin and eosin. (A) Uninfected control mice samples depicted intact intestinal epithelium with prominent villi. (B) In contrast, infected mice without treatment depicted denuded villi. Infected mice treated with (C) 100 mg/kg Paromomycin, (D) 250 mg/kg NSC10447, (E) 500 mg/kg NSC10447, and (F) 1000 mg/kg NSC10447 depicted intact intestinal epithelium with prominent villi that were comparable to the uninfected control. The images are representative of samples analyzed from 3 mice per treatment group. (G) The mean percentage of denuded villi in 4 randomly chosen microscopic fields per sample from the uninfected (Uninf), infected untreated (Inf), infected treated with 100 mg/kg paromomycin (Inf+P), infected treated with 250 mg/kg NSC10447 (Inf+250), infected treated with 500 mg/kg NSC10447 (Inf+500), and infected treated with 1000 mg/kg NSC10447 (Inf+1000) mice. The data shown represent means for samples from 3 mice per group with standard error bars and levels of statistical significance depicted (*P < 0.05).

During *C*. *parvum* infection, usually the distal small intestines are severely affected, characterized by villous atrophy, erosion and ulceration of the intestinal mucosa. Thus, we performed histopathological examination of the distal small intestines of the experimental mice at 9 days post-infection. As expected, while uninfected mice maintained the integrity of the intestinal mucosa (Fig 7A and Fig 8A), infected untreated mice had microscopic lesions characterized by villous atrophy and mucosal erosion (Figs 7B and 8B). Infected mice treated with NSC158011 maintained intact intestinal mucosa and villi (Fig 7D) whose integrity was similar to that of mice treated with paromomycin (Fig 7C). Likewise, mice treated with NSC10447 also prevented villous atrophy and maintained the integrity of the intestinal mucosa (8D-F) similar to treatment with paromomycin (Fig 8C). We enumerated the mean percentage of denuded intestinal villi in 4 randomly chosen microscopic fields per sample from representative histopathology images. We observed that in infected mice, just like treatment with paromomycin, treatment with NSC158011 and NSC10447 reduced the percentage of denuded intestinal villi by 7-fold or more compared to infected untreated mice (Fig 7E and Fig 8G). These findings corroborated the observations that treatment with NSC158011 and NSC10447, just like paromomycin, inhibited *C*. *parvum* oocysts shedding in mice’s feces to almost undetectable levels.

## Discussion

Because of the lack of genetic tools for identifying essential molecular components in *Cryptosporidium*, screening for potential drug lead-compounds against *Cryptosporidium* has been based on molecular targets identified in other protozoan parasites such as *Toxoplasma* and *Plasmodium*. However, the completed and annotated *Cryptosporidium* genome sequence shows the absence of conventional drug targets being pursued in other protozoan parasites [10]. Nevertheless, the completed genome sequence of *Cryptosporidium* has unveiled a number of bacterial-like and plant-like classic and novel drug molecular targets that now require functional characterization and validation using genetic tools [10]. Among the identified potential drug molecular targets, is the *C*. *parvum* lactate dehydrogenase (CpLDH), which is a bacterial-type lactate dehydrogenase enzyme that the parasite uses to generate metabolic energy (ATP) in the glycolytic pathway [11, 17, 18]. Importantly, *C*. *parvum* lacks both the Krebs cycle and the cytochrome-based respiration chain [10], suggesting that the glycolysis pathway is the sole energy source in *C*. *parvum* [19–21]. Consistently, using morpholino-based targeted knockdown of CpLDH, we recently showed that CpLDH is essential for growth, propagation and viability of *C*. *parvum in vitro* and *in vivo* [8, 9]. Corroboratively, previous studies have shown that known inhibitors of lactate dehydrogenase enzymes, gossypol and FX11, are able to inhibit the enzymatic activity of CpLDH [11]. However, both gossypol and FX11 are not specific to CpLDH, but also inhibit mammalian lactate dehydrogenases, implying that they would be toxic to mammalian cells. Regardless, it is noteworthy that CpLDH is unique to *C*. *parvum*, and is very significantly different from the lactate dehydrogenase enzymes found in mammals [17].

In the present study, we first established the *in vitro* enzymatic kinetic parameters of the natively purified recombinant CpLDH protein. Consistent with previous reports by others [11], we found that recombinant CpLDH preferentially catalyzed the reduction of pyruvate to lactate, and displayed Michaelis-Menten enzymatic kinetics. Using the *in vitro* enzymatic assay, we identified 29 chemical compounds that inhibited the catalytic activity of recombinant CpLDH for the reduction of pyruvate to lactate. Lactate dehydrogenase is a key enzyme for the anaerobic respiration in which pyruvate is reduced to lactate, with the concomitant oxidation of NADH to NAD^+^ [22]. Thus, we tested the candidate compounds for toxicity in a mammalian cell line (HCT-8) and selected only those that were tolerable at high micromolar concentrations (IC_50_ > 140 μM) as candidate compounds for further testing. The cytotoxicity IC_50_ values of the candidate compounds were at least 2-fold higher than the cytotoxicity IC_50_ values of known mammalian lactate dehydrogenase inhibitors (gossypol and FX11) in HCT-8 cells [11]. We subsequently tested the candidate compounds for anti-*Cryptosporidium* effect using *in vitro* infection assays of HCT-8 cells monolayers and identified compounds NSC158011 and NSC10447 that sustainably inhibited the proliferation of intracellular C. *parvum*. The HCT-8 cells were infected with excysted *C*. *parvum* sporozoites that infect host cells and transform into proliferative merozoites. In *C*. *parvum* sporozoites and merozoites, CpLDH is expressed and localized in the cytosol [18], suggesting that it is utilized for energy generation during these parasite stages that are important for host cell invasion and intracellular parasite growth. Interestingly, NSC158011 has been previously shown to inhibit the catalytic activity of the *Plasmodium faclciparum* phosphoethanolamine methyltransferase enzyme, and to inhibit *in vitro* intracellular growth of the parasite [23]. However, based on the completed genome sequence of *C*. *parvum*, there are no homologs of genes encoding a phosphoethanolamine methyltransferase in *C*. *parvum*. Therefore, our findings suggest that the anti-*Cryptopsoridium* activity of NSC158011 is associated with its ability to inhibit the catalytic activity of CpLDH which is an essential enzyme for survival and growth of *C*. *parvum*, both *in vitro* and *in vivo* [8, 9].

At amino acid sequence level, CpLDH is only 25% identical to human LDH, with the active site conformation of CpLDH being significantly different from that of the human LDH [24]. Further, in the 3-dimensional structure model of the two enzymes, the helix-loop portion of CpLDH is more proximal to the active site loop than it is in the human LDH [24]. Additionally, the co-factor binding site of human LDH possesses a network of hydrogen-bonding formed by a serine residue with NAD^+^, while the co-factor binding site of CpLDH only forms two hydrogen bonds with NAD^+^ [24]. This is thought to lower the CpLDH affinity for NAD^+^/NADH than human LDH [24]. When we modeled NSC158011 and NSC10447 onto the 3-D crystal structure of CpLDH and human LDH, we found that both NSC158011 and NSC10447 bind to the NAD+ co-factor binding site. Interestingly, in the docking simulation, NSC10447 displayed better affinity for CpLDH than human LDH, while NSC158011 displayed better affinity for the human LDH. We had selected NSC10447 and NSC158011 based on their low toxicity in a human cell line, but high inhibitory activity against *C*. *parvum*, though these molecules still bind to the human LDH crystal structure. A docking simulation calculates the free energy of the interaction between a protein and a ligand but does not consider the interaction between the ligand and its surrounding solvent (solvation energy). Due to the unfavorable high solvent exposure of the non-polar, aromatic rings in NSC158011 docked to human LDH, it can be inferred that the binding stability is greatly reduced. The docking pose of NSC158011 to CpLDH exposes the non-polar regions of the molecule to less solvent, leading to a much more stable interaction. These ligand binding properties suggest that NSC158011 and NSC10447 would more effectively compete out the binding of NAD+ to CpLDH than to human LDH. This is consistent with our observations that both NSC158011 and NSC10447 effectively inhibit *C*. *parvum* growth and replication (both *in vitro* and *in vivo*) at concentrations that are not toxic to mammalian (including human) cells.

Typically, solvent-exposed protein pockets like the one in LDH are often not targeted in lead-compound optimization due to their poor binding characteristics, but the results of our *in silico* docking reveal that solvent-exposed protein pockets may be useful for enhancing lead-compound selectivity. Importantly, these differences in ligand-binding stability between CpLDH and human LDH offer prospects for identifying inhibitors that would specifically target CpLDH, without being toxic to mammalian host cells, and would thus be potential lead-compounds for development of effective anti-*Cryptosporidium* drugs.

We observed that NSC158011’s IC_50_ for the inhibition of recombinant CpLDH in an *in vitro* enzymatic assay was higher than its IC_50_ for inhibition of *C*. *parvum* growth. Based on our observation that NSC158011 binds to the co-factor binding site in CpLDH, the likely reason for this discordance is that in the recombinant CpLDH enzymatic assay *in vitro*, excessive amounts of NADH co-factor (1 mM) were used that in turn required high concentration of NSC158011 to effectively compete out the co-factor and reduce the generation of the product. In comparison, intracellular (intra-parasite) levels of co-factor are likely much lower (μM range). For instance, in human cells the absolute concentration of NADH has been reported to be in the range of 97 to 168 μM [25, 26]. The lower intracellular concentrations of NADH when compared to the higher *in vitro* concentrations, would translate into lower concentrations of NSC158011 to effectively compete out the co-factor and register a decrease in CpLDH activity, and subsequent reduction in parasite growth. Importantly, the chemical structure of NSC158011 suggests that it possesses promising drug-like properties that render it amenable to drug development [23].

Using doses that were tolerable in mice, we tested the *in vivo* efficacies of NSC158011 and NSC10447 in Gamma interferon knockout mice (B6.129S7-Ifng) that when infected with *C*. *parvum*, develop debilitating clinical disease, with completion of the parasite life cycle and shedding of oocysts in feces [27]. We found that both NSC158011 and NSC10447 consistently significantly reduced the shedding of *C*. *parvum* oocysts during the experimental period of 9 days, and prevented the occurrence of villous atrophy and intestinal mucosal erosion that is associated with *C*. *parvum* infection. NSC158011 displayed better efficacy than NSC10447, both *in vitro* and *in vivo*, with lower anti-*Cryptosporidium* IC_50_ values. Importantly, compared to the only FDA-approved nitazoxanide that lacks efficacy in immunocompromised individuals, both NCS158011 and NSC10447 were efficacious against *C*. *parvum* in the immunocompromised mice we used in the study.

In conclusion, we have demonstrated NSC158011 and NSC10477 as specific inhibitors for CpLDH that have efficacy against *C*. *parvum* both *in vitro* and *in vivo*. Thus, our findings provide promising anti-*Cryptosporidium* drug candidates that can be explored further for the development of much needed novel cryptosporidiosis therapeutic interventions.

## Materials and methods

### Ethics statement

All experiments involving the use of mice and Holstein calves were carried out in accordance with guidelines and protocols number 17024 and 18108, respectively, approved by the University of Illinois Institutional Animal Care and Use Committee, in compliance with the United States Department of Agriculture Animal Welfare Act and the National Institute of Health Public Health Service Policy on the Humane Care and Use of Animals guidelines.

### Parasites

For all experiments, the AUCP-1 isolate of *C*. *parvum* was used. The parasites were maintained and propagated in male Holstein calves in accordance with the guidelines of protocol number 18108 approved by the University of Illinois at Urbana-Champaign, USA. Freshly shed *C*. *parvum* oocysts in calf feces were extracted and purified by sequential sieve filtration, Sheather's sugar flotation, and discontinuous sucrose density gradient centrifugation, essentially as previously described [28, 29]. The purified oocysts were rinsed and stored at 4°C in 50 mM Tris–10 mM EDTA, pH 7.2, and used within 3 months. Sporozoites were excysted from *C*. *parvum* oocysts following the method described previously [30]. Briefly, to about 1 × 10^8^ purified *C*. *parvum* oocysts suspended in 500 μl of PBS, an equal volume of 40% commercial laundry bleach was added and incubated for 10 minutes at 4°C. The oocysts were washed four times in PBS containing 1% (w/v) bovine serum albumin and resuspended in Hanks balanced salt solution, incubated for 60 minutes at 37°C, and mixed with an equal volume of warm 1.5% sodium taurocholate in Hanks balanced salt solution. The excysted sporozoites were collected by centrifugation and resuspended in supplemented PBS. The sporozoites were purified by passing the suspension through a sterile 5 μM syringe filter (Millex) and enumerated with a hemocytometer.

### Biochemical assays

The coding sequence of CpLDH was cloned from cDNA prepared from the AUCP-1 isolate of *C*. *parvum*, and the His-tagged CpLDH recombinant protein expressed in *Escherichia coli*, and purified in native form essentially as previously described [8]. Briefly, cDNA was prepared from total RNA extracted from the AUCP-1 isolate of *C*. *parvum*, and the coding sequence of CpLDH (Genebank accession number AF274310.1) was PCR-amplified from the cDNA using the primer pair 5’-*CTCGAG***ATG**ATTGAAAGACGCAAGA-3’ (Forward, with the *XhoI* restriction site italicized and start codon in bold) and 5’-*GGATCC***TTA**TGCTCCAGCTGGT-3’ (Reverse, with the *BamHI* site italicized and stop codon in bold). The PCR amplicon was cloned at the *XhoI/BamHI* site of the pET15b expression vector in-frame with the hexahistidine (His-tag) at the N-terminal and sequenced to confirm identity. The recombinant expression vector was transformed into protein expression *E*. *coli* BL21-CodonPlus-DE3-RIL (Stratagene). Transformed *E*. *coli* was cultured at 37°C in Luria broth medium (supplemented with 100 μg/ml ampicillin and 34 μg/ml chloramphenicol) to an *A*_600_ of 0.8 followed by addition of 1 mM isopropyl-β-d-thiogalactopyranoside to induce protein expression. The expression *E*. *coli* was harvested and lysed under native conditions by sonicating in lysis buffer (50 mM NaH_2_PO_4_, 300 mM NaCl, 10 mM Imidazole, pH 8.0) containing a 1x EDTA-free protease inhibitor cocktail, 600 units benzonase and 30 kU lysozyme (EMD Millipore). The lysate was clarified by centrifugation and the His-tagged recombinant protein purified under native conditions by nickel-affinity chromatography according to the manufacturer's instructions (Novagen). The wash buffer used contained 50 mM NaH_2_PO_4_, 300 mM NaCl and 20 mM Imidazole, pH 8.0, while the elution buffer was composed of 50 mM NaH_2_PO_4_, 300 mM NaCl, 250 mM Imidazole, pH 8.0. The eluate was dialyzed using a buffer containing 5 mM Hepes–KOH (pH 7.8) and 0.5 mM DTT. The purity of the recombinant protein was determined by SDS/PAGE, and the concentration measured using a Qubit 3.0 fluorometer (Life technologies). The *in vitro* enzymatic activity of the recombinant CpLDH protein for catalyzing the reduction of pyruvate to lactate was determined by measuring the change in optical density of a 100 μl reaction mixture containing 10 mM pyruvate, 1 mM NADH, 100 mM Tris, pH 7.5 and varying concentrations of CpLDH recombinant protein at 25°C. On the other hand, the catalytic activity of CpLDH recombinant protein for the oxidation of lactate to pyruvate was determined by measuring the change in optical density of a 100 μl reaction mixture containing 100 mM lactate, 1 mM NAD^+^, 100 mM Tris, pH 9.2, with varying concentrations of CpLDH recombinant protein at 25°C. For determining the kinetic parameters, a fixed concentration of CpLDH recombinant protein was used in reactions with varying substrate and co-factor concentrations (pyruvate from 0.5–15 mM; NADH from 0.25–1.5 mM for the reduction reaction, while for the oxidation reaction lactate varied from 25–125 mM; NAD^+^ from 0.05–1.5 mM). In all assays, reaction mixtures without recombinant CpLDH protein were included as negative controls. All assays were performed in triplicate and repeated at least thrice. The change in optical density was measured every 15 seconds for a total of 2 minutes using a Spectra Max 384 Plus plate reader (Molecular Devices) at a wave length of 340 nm.

### Screening compounds for inhibitory activity against CpLDH

The chemical compounds were obtained from the National Cancer Institute/Developmental Therapeutics Program Open Chemical Repository. They consisted of a diverse set of compounds (n = 27) (S1 Table) reported previously [12], and a Mechanistic Set IV compounds (n = 800) (S2 Table). The compounds were reconstituted in dimethyl sulfoxide (DMSO) as stock solutions. Just before use, aliquots of the stock solutions were diluted in sterile distilled water to generate working solutions, such that the final amount of DMSO added to the reaction mixtures was less than 1% (V/V). The compounds were tested for their inhibitory effect against the enzymatic activity of recombinant His-tagged CpLDH for catalyzing the reduction of pyruvate to lactate. The reactions were performed in 100 μl reaction volume containing 10 mM pyruvate, 1 mM NADH, 100 mM Tris, pH 7.5, 15 ng/μl of recombinant CpLDH protein with or without 20 μM of compound. Control reactions without recombinant CpLDH protein were included. Reactions were performed in triplicate and repeated at least thrice. The change in optical density was measured every 15 seconds for a total of 2 minutes using a Spectra Max 384 Plus plate reader (Molecular Devices) at a wave length of 340 nm. The mean percent inhibition effect of each compound on recombinant CpLDH activity was derived using the following formula:

Mean Percent Inhibition (MPI) = (ΔOD_340_ of reaction with compound / ΔOD_340_ of reaction without compound) X 100

Where:

- ΔOD_340_ is the mean change in optical density for triplicate reactions after 2 minutes.
- MPI values greater and less than 0 indicate inhibition and augmentation of CpLDH activity, respectively.
- MPI value of 0 is for the reaction without compound, but with an equivalent volume of solvent used to reconstitute compound.

### Compound cytotoxicity assay

Compounds with inhibitory effect against the enzymatic activity of recombinant CpLDH were tested for cytotoxicity in a human cell line, HCT-8 (American Type Culture Collection Item number: CCL244), that was used for *in vitro* culture of *C*. *parvum*. A colorimetric assay using the cell proliferation reagent WST-1 (Roche, USA) for the quantification of cell viability was performed. HCT-8 cells were cultured in 96-well plates in 200 μl of RPMI 1640 medium without phenol red (Life Technologies), but supplemented with 2 g/L of sodium bicarbonate, 2.5 g/L of glucose, 10% FBS (Gibco, USA), 1× antibiotic–antimycotic (Gibco), and 1× sodium pyruvate (Gibco). When the cells were confluent, the old medium was replaced with fresh medium with or without varying concentrations of chemical compound. After 24 h of culture, 10 μl of the cell proliferation reagent WST-1, (for quantification of cell viability) was added to each well, mixed and the plates incubated for 1 h at 37 C with 5% CO_2_ in the dark. Following incubation, 150 μL of the medium from each well was transferred to a new 96-well plate and quantification of the formazan dye produced by metabolically active cells was read as absorbance at a wavelength of 420 nm using a scanning multi-well spectrophotometer (Spectra Max 384 Plus; Molecular Devices, USA). Three independent assays were performed and the dose–response curves of the means of triplicate assays were generated using GraphPad PRISM software to derive the half maximal inhibitory concentration (IC_50_) of compounds in HCT-8 cells.

### *In vitro* testing of the anti-*Cryptosporidium* effect of CpLDH inhibitors

HCT-8 cells were cultured in supplemented RPMI-1640 medium in 96-well plates. When the cells were confluent, old medium was replaced with fresh medium. To one set of wells, varying concentrations of recombinant CpLDH inhibitors (reconstituted in DMSO and diluted in RPMI medium) were added, while another set was left without inhibitors. Paromomycin was used as a positive control drug reconstituted in distilled sterile water. Then, 4 x 10^4^ freshly excysted sporozoites were added to each well and incubated at 37°C with 5% CO_2_. After 2 h incubation, varying concentrations of CpLDH inhibitors were added to the set of infected cells that were not treated initially. Control infected cells without inhibitors, but with added DMSO volumes equivalent to those used in the wells with inhibitors, were included. The cells were maintained in culture for a total of either 48 h or 72 h and processed for immunofluorescence assay as previously described [8, 16]. Briefly, medium was decanted and the cells fixed with methanol-acetic acid (9:1) for 2 minute at room temperature. The cells were rehydrated and permeabilized by two successive washes with buffer (0.1% Triton X-100, 0.35 M NaCl, 0.13 M Tris-base, pH 7.6) and blocked with 5% normal goat serum, followed by staining with antibody to *C*. *parvum* (SporoGlo; Waterborn, Inc.) overnight at 4°C. The stained cells were washed twice with PBS, followed by water, and then imaged with an inverted fluorescence microscope. Fluorescence quantification was done using ImageJ version 1.37v software (NIH). Assays were performed in triplicate and repeated at least thrice.

### *In silico* modeling of NSC158011 and NSC10447 binding to CpLDH and Human LDH

To propose a model for the specific binding of NSC15801 and NSC10447 to lactate dehydrogenase, the crystal structure of CpLDH complexed with substrate pyruvate and cofactor analogue 3-acetylpyridine adenine dinucleotide (APAD) was obtained from the RCSB protein database (4ND2). The chemical structures of NSC158011 and NSC10447 were obtained from the PubChem library. The protein crystal structure was loaded into the AutoDockTools software suite and a search location box was drawn encompassing the co-factor analogue APAD in one subunit of the homotetrameric protein. APAD was removed from the active site of the crystal structure using the Swiss-Model DeepView software. Using Autodock Vina (Scripps Institute, USA) as previously done [13], polar hydrogen atoms were added to the APAD-deficient LDH structure and its non-polar hydrogen atoms were merged. NSC15801 and NSC10447 were each docked into the empty co-factor binding site of one subunit of the protein with exhaustiveness = 10. Both compounds were docked using a 40 × 40 × 40 Å grid box, and all single bonds within the ligands were set to allow free rotation. The procedure was subsequently repeated with the crystal structure of human LDH (1I0Z) and a 24 × 14 × 20 Å grid box around the co-factor binding pocket of the new structure. Docking results were visualized using VMD (University of Illinois at Urbana-Champaign, USA) as previously done [31]. The most energetically favorable result of each docking was then loaded as a protein-ligand complex into the Schrödinger Maestro software (Schrodinger LLC, USA) to investigate the nature of the protein-ligand interactions and propose a mechanism for the lead compounds’ inhibition of LDH. Because the presence or absence of natural LDH substrate, pyruvate, complexed within the active site did not significantly affect binding of the compounds in CpLDH or human LDH, those models were excluded in the final molecular docking simulations.

### *In vivo* testing of the anti-*Cryptosporidium* effect of CpLDH inhibitors

Gamma interferon knockout mice (B6.129S7-Ifng), 8 weeks of age, were purchased from Charles River, USA. The care and use of the mice was done following the guidelines of protocol number 17024 approved by the University of Illinois at Urbana-Champaign, USA. The animals were allowed to acclimatize for 1 week before experiments commenced. Stock solutions of recombinant CpLDH inhibitors reconstituted in DMSO were diluted in sterile distilled water to reduce the final amount of DMSO in the solution to less than 1% (v/v) before administering them to mice. Prior to testing the anti-*Cryptosporidium* effect of the inhibitors in mice, the tolerability of each inhibitor was tested by oral gavage using varying dosages (100–2000 mg/kg body weight) of each inhibitor in groups of mice (three mice for each dose) daily for 7 days. Mice were observed daily for signs of toxicity including changes from normal physical activity, respiration, body temperature, feeding, posture, fur condition or occurrence of death. The highest dose (1000 mg/kg for NSC10447, and 400 mg/kg for NSC158011) of each inhibitor that did not induce any toxicity signs over the 7 days of administration was used as the maximum dose limit for subsequent *in vivo* experiments. The subsequent dosages of NSC10447 used in the mice infection assays were 250, 500 and 1000 mg/kg mouse body weight, while the NSC158011 dosages used were 100, 200 and 400 mg/kg mouse body weight. Mice were allocated to groups as follows: “Infected plus inhibitor treatment”; “Infected minus inhibitor treatment”; “Uninfected minus inhibitor treatment” and “Infected plus paromomycin treatment”. Each group contained at least three mice. Each mouse in the infection groups received 5,000 *C*. *parvum* oocysts (resuspended in 50 μl of PBS) by oral gavage. Mice were housed individually in cages lined with sterile gauze as bedding. One day post-infection (PI), daily oral gavage administration of recombinant CpLDH inhibitor or paromomycin commenced and continued for a total of 7 days. Untreated mice received an equivalent volume of sterile distilled water (containing DMSO equivalent to the amount administered in the inhibitor-treated group) by oral gavage. Fecal pellets were collected daily from each cage and placed in individual sterile 15 ml conical tubes. An equivalent volume of PBS containing a cocktail of penicillin (100 units/ml), streptomycin (100 μg/ml), chloramphenicol (34 μg/ml) and amphotericin (0.25 μg/ml) was added to the fecal samples and stored at 4°C until use for quantification of *C*. *parvum* genomic DNA load. Three independent replicate infection assays were performed. At 9 days PI, mice were euthanized and 5 cm of the distal small intestine resected 2 cm anterior to the cecum and immediately submerged in 4% buffered formalin. The intestinal tissues were submitted for histopathology to the Veterinary Diagnostic Laboratory at the University of Illinois at Urbana-Champaign. Briefly, intestinal tissues preserved in 4% buffered formalin were washed in 70% ethanol and embedded in 1% agar and then processed for paraffin embedding. For hematoxylin and eosin staining, five μm transverse and cross sections were cut and processed and stained following standard procedures of the Veterinary Diagnostic Laboratory. Sections were imaged using a Zeiss microscope and images captured with a color camera.

### Quantification of *C*. *parvum* shed in mice feces

Genomic DNA was extracted from individual fecal samples collected from mice at different days as described above. For each sample, 220 mg of homogenized feces were used to extract genomic DNA using the QIAamp PowerFecal DNA Kit (Qiagen, USA) following the manufacturer’s instructions. Quantification of the amount of *C*. *parvum* 18s rRNA gene (GenBank accession number AF164102) was performed essentially as described previously [9]. Briefly, the primer pair 5′-CTGCGAATGGCTCATTATAACA-3′ (Forward), and 5′-AGGCCAATACCCTACCGTCT-3′ (Reverse) was used to generate a 240 bp amplicon from *C*. *parvum* genomic DNA by conventional PCR. The PCR product was fractionated on agarose gel, extracted using the QIAquick Gel extraction kit (Qiagen, USA), and the concentration measured by Nanodrop Spectrophotometer (Fisher, USA). Ten-fold serial dilutions of the extracted DNA fragment were made and used as quantification standards for real-time PCR. Each real time PCR mixture contained 2 μl of DNA template, 1 μl of primer mix (500 nM each), and 10 μl of SsoAdvanced Universal SYBR Green Supermix (Bio-Rad, USA), with the final volume made up to 20 μl with nuclease-free water. The cycling conditions included an initial denaturation for 10 min at 98°C, 40 cycles at 98°C for 15 s and 60°C for 1 min, and a final melting curve step. Cycling was performed using a 7500 Real Time PCR System (Applied Biosystems, USA). DNA quantities were derived by the system software using the generated quantification standard curves.

### Statistical analyses

Statistical analyses were performed using two-tailed Student’s t test. *P* values of 0.05 or less were considered significant.

## Supporting information

S1 Table Cytotoxicity IC_50_ values in HCT-8 cells for inhibitors of CpLDH. (DOC)

S2 Table Diverse Set compounds. (DOCX)

S3 Table Mechanistic Set IV compounds. (DOC)

S4 Table CpLDH inhibition (%) values for mechanistic Set IV compounds. (DOCX)

S1 Fig Chemical structures of NSC158011 (A) and NSC10447 (B).
(TIF)

S2 Fig *In silico* modeling of the docking of compound NSC158011 to *Cryptosporidium parvum* lactate dehydrogenase protein (CpLDH). (**A**) Configuration of NSC158011 in the NADH (co-factor) binding site of CpLDH (4ND2). NSC158011 is shown as a stick model with bonds to carbon, Sulphur and nitrogen depicted in gray, yellow and blue, respectively. The various amino acid residues lining the pocket are also depicted as stick models (green, cyan, blue and red). (**B**) Hydrogen bonding and hydrophobic residue contacts between docked NSC158011 and CpLDH’s NADH-binding site.
(TIF)

S3 Fig *In silico* modeling of the docking of compound NSC158011 to human LDH protein. (**A**) Configuration of NSC158011 in the NADH (co-factor) binding site of human LDH (1I0Z). NSC158011 is shown as a stick model with bonds to carbon and sulphur depicted in gray and yellow, respectively. The various amino acid residues lining the pocket are also depicted as stick models (green, cyan, blue and red). (**B**) Hydrogen bonding and hydrophobic residue contacts between docked NSC158011 and human LDH’s NADH-binding site.
(TIF)

S4 Fig *In silico* modeling of the docking of compound NSC10447 to *Cryptosporidium parvum* lactate dehydrogenase protein (CpLDH). (**A**) Configuration of NSC10447 in the NADH (co-factor) binding site of CpLDH (4ND2). NSC10447 is shown as a stick model with bonds to carbon and oxygen depicted in gray and red, respectively. The various amino acid residues lining the pocket are also depicted as stick models (green, cyan and blue). (**B**) Hydrogen bonding and hydrophobic residue contacts between docked NSC10447 and CpLDH’s NADH-binding site.
(TIF)

S5 Fig *In silico* modeling of the docking of compound NSC10447 to human LDH. (**A**) Configuration of NSC10447 in the NADH (co-factor) binding site of human LDH (1I0Z). NSC10447 is shown as a stick model with bonds to carbon and oxygen depicted in gray and red, respectively. The various amino acid residues lining the pocket are also depicted as stick models (green and cyan). (**B**) Hydrogen bonding and hydrophobic residue contacts (between docked NSC10447 and human LDH’s NADH-binding site.
(TIF)
