# Supplementary material for: Novel lactate dehydrogenase inhibitors with in vivo efficacy against Cryptosporidium parvum
Source: PLoS Pathog. 2019 Jul 29;15(7):e1007953. doi: 10.1371/journal.ppat.1007953 (PMC6687188; doi:10.1371/journal.ppat.1007953)
Supplement: S1 Table — (DOC) [file ppat.1007953.s001.doc]

**S1** Table. Diverse set of compounds.

| **NSC NUMBER** | **MOLECULAR FORMULAR** | **MOLECULAR WEIGHT** |
| --- | --- | --- |
| 7346 | C10H12CuN2O8.2Na | 398.0 |
| 22225 | C9H4Cl6O4.Cd | 501.0 |
| 24048 | C21H18N2O.HI | 442.0 |
| 37031 | C20H14N2O7S2 | 458.0 |
| 39225 | C12H20N2O2 | 224.0 |
| 46613 | C13H13N5 | 239.0 |
| 47924 | C18H17NO2 | 279.0 |
| 57998 | C13H19NS.C7H4ClNO3 | 407.0 |
| 85459 | C16H24N2O4 | 308.0 |
| 88947 | C17H13NO8S2.Na | 446.0 |
| 109268 | C32H36Cl2Cu2N2O2 | 679.0 |
| 113997 | C14H10O4S2 | 306.0 |
| 125034 | C18H12N2O2S2 | 352.0 |
| 150080 | C12H17N3O3 | 251.0 |
| 158011 | C18H15NS2 | 309.0 |
| 169942 | C16H28Cu2N8O8 | 588.0 |
| 173904 | C24H29ClN2O4 | 445.0 |
| 175493 | C9H11FeNOS2 | 269.0 |
| 310551 | C18H20CuN6S4 | 512.0 |
| 323241 | C16H22N4Se | 349.0 |
| 348401 | C11H6N8O3S | 330.0 |
| 371777 | C15H14N2O4S | 318.0 |
| 622648 | C8H13N2S3.ClO4 | 333.0 |
| 632233 | C16H15NO2S | 285.0 |
| 638646 | C26H24Cl4N2O3.ClH | 591.0 |
| 641296 | C13H19ClCuN4S | 362.0 |
| 668394 | C17H12Br2N2O3 | 452.0 |
