# Supplementary material for: Novel lactate dehydrogenase inhibitors with in vivo efficacy against Cryptosporidium parvum
Source: PLoS Pathog. 2019 Jul 29;15(7):e1007953. doi: 10.1371/journal.ppat.1007953 (PMC6687188; doi:10.1371/journal.ppat.1007953)
Supplement: S2 Table — (DOCX) [file ppat.1007953.s002.docx]

**S2 Table. Mechanistic Set IV compounds**

| **PLATE KEY** | **PLATE NUMBER** | **WELL NUMBER** | **NSC NUMBER** | **MOLECULAR WEIGHT** | **MOLECULAR**  **FORMULA** |
| --- | --- | --- | --- | --- | --- |
| 4847057 | 4847 | A02 | 1026 | 129 | C6H11NO2 |
| 4847057 | 4847 | A03 | 295156 | 124 | C7H8O2 |
| 4847057 | 4847 | A04 | 755 | 152 | C5H4N4S |
| 4847057 | 4847 | A05 | 614928 | 160 | C8H16O3 |
| 4847057 | 4847 | A06 | 51148 | 183 | CdCl2 |
| 4847057 | 4847 | A07 | 369317 | 180 | C8H5FN2S |
| 4847057 | 4847 | A08 | 176324 | 195 | C5H7ClN2O4 |
| 4847057 | 4847 | A09 | 63446 | 213 | C6H7N5O2S |
| 4847057 | 4847 | A10 | 357683 | 223 | C10H9NO5 |
| 4847057 | 4847 | A11 | 693053 | 220 | C11H12N2O3 |
| 4847057 | 4847 | B02 | 3970 | 112 | C4H4N2O2 |
| 4847057 | 4847 | B03 | 118976 | 149 | C8H7NS |
| 4847057 | 4847 | B04 | 11779 | 168 | C10H16O2 |
| 4847057 | 4847 | B05 | 695218 | 159 | C7H13NO3 |
| 4847057 | 4847 | B06 | 54297 | 182 | C7H6N2S2 |
| 4847057 | 4847 | B07 | 369318 | 190 | C10H10N2S |
| 4847057 | 4847 | B08 | 620050 | 198 | C9H10O3S |
| 4847057 | 4847 | B09 | 107415 | 217 | C13H15NO2 |
| 4847057 | 4847 | B10 | 606532 | 213 | C7H7N3O5 |
| 4847057 | 4847 | B11 | 1771 | 240 | C6H12N2S4 |
| 4847057 | 4847 | C02 | 4728 | 101 | C2H3N3S |
| 4847057 | 4847 | C03 | 261726 | 150 | C6H6N4O |
| 4847057 | 4847 | C04 | 18804 | 164 | C10H12O2 |
| 4847057 | 4847 | C05 | 3852 | 174 | C9H6N2O2 |
| 4847057 | 4847 | C06 | 99733 | 179 | C6H5N5O2 |
| 4847057 | 4847 | C07 | 625639 | 178 | C9H10N2O2 |
| 4847057 | 4847 | C08 | 625748 | 192 | C8H12N6 |
| 4847057 | 4847 | C09 | 128734 | 221 | C12H7N5 |
| 4847057 | 4847 | C10 | 403883 | 222 | C15H26O |
| 4847057 | 4847 | C11 | 3905 | 233 | C15H11N3 |
| 4847057 | 4847 | D02 | 4960 | 124 | C7H8O2 |
| 4847057 | 4847 | D03 | 285166 | 145 | C9H7NO |
| 4847057 | 4847 | D04 | 18805 | 164 | C10H12O2 |
| 4847057 | 4847 | D05 | 4170 | 172 | C11H8O2 |
| 4847057 | 4847 | D06 | 111041 | 189.17 | C9H7N3O2 |
| 4847057 | 4847 | D07 | 61805 | 196 | C10H12O4 |
| 4847057 | 4847 | D08 | 664298 | 204 | C12H16N2O |
| 4847057 | 4847 | D09 | 132493 | 218 | C11H10N2O3 |
| 4847057 | 4847 | D10 | 635441 | 220 | C11H12N2O3 |
| 4847057 | 4847 | D11 | 5200 | 230 | C10H10N6O |
| 4847057 | 4847 | E02 | 5354 | 108 | C6H8N2 |
| 4847057 | 4847 | E03 | 529469 | 149 | C3H7N3O4 |
| 4847057 | 4847 | E04 | 37364 | 165 | C6H7N5O |
| 4847057 | 4847 | E05 | 4857 | 172 | C5H11NS2.Na |
| 4847057 | 4847 | E06 | 163501 | 179 | C5H7ClN2O3 |
| 4847057 | 4847 | E07 | 95580 | 200 | C10H20N2O2 |
| 4847057 | 4847 | E08 | 673912 | 191 | C10H13N3O |
| 4847057 | 4847 | E09 | 159935 | 224 | C10H12N2O4 |
| 4847057 | 4847 | E10 | 635975 | 206 | C13H18O2 |
| 4847057 | 4847 | E11 | 7210 | 240 | C14H8O4 |
| 4847057 | 4847 | F02 | 65381 | 112 | C4H4N2O2 |
| 4847057 | 4847 | F03 | 626433 | 142 | C4H2N2O4 |
| 4847057 | 4847 | F04 | 349438 | 168.19 | C9H12O3 |
| 4847057 | 4847 | F05 | 11897 | 188 | C11H8O3 |
| 4847057 | 4847 | F06 | 253272 | 189 | C6H11N3O4 |
| 4847057 | 4847 | F07 | 95678 | 196 | C7H8N4OS |
| 4847057 | 4847 | F08 | 1620 | 215.21 | C10H9N5O |
| 4847057 | 4847 | F09 | 166464 | 216 | C9H10ClNOS |
| 4847057 | 4847 | F10 | 657449 | 212 | C8H8N2O3S |
| 4847057 | 4847 | F11 | 7833 | 238 | C14H10N2O2 |
| 4847057 | 4847 | G02 | 89303 | 122 | C7H6O2 |
| 4847057 | 4847 | G03 | 635968 | 141 | C7H11NO2 |
| 4847057 | 4847 | G04 | 400944 | 159 | C9H5NO2 |
| 4847057 | 4847 | G05 | 40212 | 177.03 | C6H6Cl2N2 |
| 4847057 | 4847 | G06 | 326397 | 187 | C7H4Cl2N2 |
| 4847057 | 4847 | G07 | 98447 | 199 | C10H5N3O2 |
| 4847057 | 4847 | G08 | 4280 | 208 | C14H12N2 |
| 4847057 | 4847 | G09 | 296961 | 214.22 | C5H15N2O3PS |
| 4847057 | 4847 | G10 | 666526 | 223 | C10H16Cl2O |
| 4847057 | 4847 | G11 | 11905 | 242 | C15H14O3 |
| 4847057 | 4847 | H02 | 174280 | 111 | C4H8O2.Na |
| 4847057 | 4847 | H03 | 749 | 152 | C4H4N6O |
| 4847057 | 4847 | H04 | 603719 | 151 | C7H5NO3 |
| 4847057 | 4847 | H05 | 45388 | 182.18 | C6H10N6O |
| 4847057 | 4847 | H06 | 347466 | 175 | H2O3Se.2Na |
| 4847057 | 4847 | H07 | 148958 | 200 | C8H9FN2O3 |
| 4847057 | 4847 | H08 | 26045 | 216 | C8H3Cl2NO2 |
| 4847057 | 4847 | H09 | 326231 | 222.3 | C8H18N2O3S |
| 4847057 | 4847 | H10 | 688795 | 220 | C12H12O4 |
| 4847057 | 4847 | H11 | 18938 | 244 | C14H28O3 |
| 4848057 | 4848 | A02 | 21548 | 242 | C10H14N2O5 |
| 4848057 | 4848 | A03 | 126849 | 243 | C10H13NO6 |
| 4848057 | 4848 | A04 | 375575 | 239 | 375575 |
| 4848057 | 4848 | A05 | 664286 | 241 | C14H15N3O |
| 4848057 | 4848 | A06 | 10447 | 256 | C14H8O5 |
| 4848057 | 4848 | A07 | 65423 | 252.23 | C10H12N4O4 |
| 4848057 | 4848 | A08 | 146604 | 262 | C9H11FN2O6 |
| 4848057 | 4848 | A09 | 283162 | 258.28 | C9H18N6O3 |
| 4848057 | 4848 | A10 | 400978 | 248 | C15H20O3 |
| 4848057 | 4848 | A11 | 667235 | 248 | C13H12O5 |
| 4848057 | 4848 | B02 | 33004 | 227 | C13H9NOS |
| 4848057 | 4848 | B03 | 169543 | 244 | C7H8N4O2S2 |
| 4848057 | 4848 | B04 | 379531 | 231 | C8H4Cl2N2S |
| 4848057 | 4848 | B05 | 664331 | 228 | C13H12N2O2 |
| 4848057 | 4848 | B06 | 13966 | 246 | C12H10N2O2S |
| 4848057 | 4848 | B07 | 80087 | 247 | C18H17N |
| 4848057 | 4848 | B08 | 175274 | 263 | C16H13N3O |
| 4848057 | 4848 | B09 | 286193 | 260.26 | C9H12N2O5S |
| 4848057 | 4848 | B10 | 405158 | 257 | C14H9ClN2O |
| 4848057 | 4848 | B11 | 667251 | 248 | C17H16N2 |
| 4848057 | 4848 | C02 | 56544 | 228 | C13H8O2S |
| 4848057 | 4848 | C03 | 175634 | 244 | C13H12N2OS |
| 4848057 | 4848 | C04 | 602617 | 238 | C14H10N2O2 |
| 4848057 | 4848 | C05 | 674495 | 240 | C14H12N2S |
| 4848057 | 4848 | C06 | 15200 | 259 | Ga.3HNO3 |
| 4848057 | 4848 | C07 | 85236 | 262.3 | C15H18O4 |
| 4848057 | 4848 | C08 | 176655 | 265 | C16H11NO3 |
| 4848057 | 4848 | C09 | 292147 | 250 | C15H10N2O2 |
| 4848057 | 4848 | C10 | 634471 | 265 | C9H10Cl2N2O3 |
| 4848057 | 4848 | C11 | 676561 | 247 | C13H13NO2S |
| 4848057 | 4848 | D02 | 66914 | 226 | C7H7Cl3N2 |
| 4848057 | 4848 | D03 | 299879 | 235 | C11H9NO5 |
| 4848057 | 4848 | D04 | 607347 | 239 | C9H9Cl2FS |
| 4848057 | 4848 | D05 | 697726 | 234 | C12H14N2O3 |
| 4848057 | 4848 | D06 | 22194 | 256 | C18H24O |
| 4848057 | 4848 | D07 | 85998 | 265.22 | C8H15N3O7 |
| 4848057 | 4848 | D08 | 191384 | 265 | C18H19NO |
| 4848057 | 4848 | D09 | 307454 | 259 | C9H11ClN4O3 |
| 4848057 | 4848 | D10 | 634650 | 252 | C16H12O3 |
| 4848057 | 4848 | D11 | 7364 | 269 | C11H10Cl2N4 |
| 4848057 | 4848 | E02 | 86100 | 234 | C16H14N2 |
| 4848057 | 4848 | E03 | 319726 | 234.32 | C11H14N4S |
| 4848057 | 4848 | E04 | 625355 | 230 | C9H11FN2O4 |
| 4848057 | 4848 | E05 | 186 | 250 | C13H14O5 |
| 4848057 | 4848 | E06 | 29603 | 263 | C11H10N6.ClH |
| 4848057 | 4848 | E07 | 105808 | 253 | C9H5BrN2O2 |
| 4848057 | 4848 | E08 | 191389 | 251 | C17H17NO |
| 4848057 | 4848 | E09 | 310618 | 246 | C15H18O3 |
| 4848057 | 4848 | E10 | 637729 | 250 | C15H10N2O2 |
| 4848057 | 4848 | E11 | 9856 | 282 | C18H34O2 |
| 4848057 | 4848 | F02 | 87206 | 232 | C16H12N2 |
| 4848057 | 4848 | F03 | 328587 | 245 | C8H10N2O2Se |
| 4848057 | 4848 | F04 | 635563 | 238 | C8H6N4O3S |
| 4848057 | 4848 | F05 | 750 | 246.29 | C6H14O6S2 |
| 4848057 | 4848 | F06 | 32946 | 257.13 | C5H12N8.2ClH |
| 4848057 | 4848 | F07 | 119686 | 263 | C17H13NS |
| 4848057 | 4848 | F08 | 263500 | 257 | C12H14N2.2Cl |
| 4848057 | 4848 | F09 | 320846 | 249.27 | C15H11N3O |
| 4848057 | 4848 | F10 | 643028 | 246 | C16H10N2O |
| 4848057 | 4848 | F11 | 9706 | 204 | C9H12N6 |
| 4848057 | 4848 | G02 | 99027 | 239 | C10H7BrO2 |
| 4848057 | 4848 | G03 | 373853 | 231 | C8H7ClN2O2S |
| 4848057 | 4848 | G04 | 637833 | 238 | C7H10O3S3 |
| 4848057 | 4848 | G05 | 1906 | 246 | C6H11NS2.C5H11N |
| 4848057 | 4848 | G06 | 54650 | 253 | C15H11NO3 |
| 4848057 | 4848 | G07 | 145150 | 260 | C15H16O4 |
| 4848057 | 4848 | G08 | 267213 | 258 | C9H10N2O5S |
| 4848057 | 4848 | G09 | 336628 | 263 | C11H9N3O3S |
| 4848057 | 4848 | G10 | 643031 | 246 | C16H10N2O |
| 4848057 | 4848 | G11 | 659501 | 258 | C13H10N2O2S |
| 4848057 | 4848 | H02 | 118742 | 233 | C8H16N6.ClH |
| 4848057 | 4848 | H03 | 375294 | 234 | C15H22O2 |
| 4848057 | 4848 | H04 | 645987 | 242 | C12H10N4O2 |
| 4848057 | 4848 | H05 | 4114 | 250 | C5H9NOS2.C4H9NO |
| 4848057 | 4848 | H06 | 63984 | 251 | C10H13N5O3 |
| 4848057 | 4848 | H07 | 146268 | 261 | C8H11N3O5S |
| 4848057 | 4848 | H08 | 278619 | 256 | C8H17N3O4.ClH |
| 4848057 | 4848 | H09 | 382007 | 260 | C12H14ClNO.ClH |
| 4848057 | 4848 | H10 | 663996 | 246 | C14H18N2O2 |
| 4848057 | 4848 | H11 | 106296 | 262 | C17H14N2O |
| 4849057 | 4849 | A02 | 13973 | 274 | C15H15N3.ClH |
| 4849057 | 4849 | A03 | 69187 | 276 | C18H16N2O |
| 4849057 | 4849 | A04 | 89671 | 280.36 | C16H24O4 |
| 4849057 | 4849 | A05 | 154754 | 270 | C9H8BrN3O2 |
| 4849057 | 4849 | A06 | 208914 | 280 | C15H24N2O3 |
| 4849057 | 4849 | A07 | 605756 | 268 | C13H11N3.8/5ClH |
| 4849057 | 4849 | A08 | 635404 | 281 | C15H11N3O3 |
| 4849057 | 4849 | A09 | 643910 | 276 | C14H16N2O2S |
| 4849057 | 4849 | A10 | 31702 | 287 | C18H13N3O |
| 4849057 | 4849 | A11 | 119875 | 300.06 | Cl2H6N2Pt |
| 4849057 | 4849 | B02 | 19994 | 283 | C10H13N5O5 |
| 4849057 | 4849 | B03 | 70422 | 267 | C10H13N5O4 |
| 4849057 | 4849 | B04 | 97703 | 274 | C19H14O2 |
| 4849057 | 4849 | B05 | 155595 | 284 | C16H14ClN3 |
| 4849057 | 4849 | B06 | 264880 | 283 | C8H14N4O5.ClH |
| 4849057 | 4849 | B07 | 621889 | 283 | C9H18NO.I |
| 4849057 | 4849 | B08 | 636817 | 283 | C17H17NOS |
| 4849057 | 4849 | B09 | 654705 | 273 | C15H15NO4 |
| 4849057 | 4849 | B10 | 36826 | 292 | C12H10ClN5S |
| 4849057 | 4849 | B11 | 168221 | 286 | C15H14N2O2S |
| 4849057 | 4849 | C02 | 22842 | 268.23 | C15H8O5 |
| 4849057 | 4849 | C03 | 71851 | 283 | C10H13N5O3S |
| 4849057 | 4849 | C04 | 102811 | 267 | C10H13N5O4 |
| 4849057 | 4849 | C05 | 165897 | 274 | C14H14N2O2S |
| 4849057 | 4849 | C06 | 269142 | 282 | C15H22O5 |
| 4849057 | 4849 | C07 | 622627 | 284.57 | C8H4ClF3N2O4 |
| 4849057 | 4849 | C08 | 637731 | 277 | C18H15NO2 |
| 4849057 | 4849 | C09 | 655255 | 274 | C15H14O5 |
| 4849057 | 4849 | C10 | 39202 | 292 | C18H32N2O |
| 4849057 | 4849 | C11 | 175296 | 286 | C16H14O5 |
| 4849057 | 4849 | D02 | 22992 | 271.74 | C13H17NO3.ClH |
| 4849057 | 4849 | D03 | 72961 | 268 | C9H12N6O4 |
| 4849057 | 4849 | D04 | 105014 | 285.69 | C10H12ClN5O3 |
| 4849057 | 4849 | D05 | 169779 | 268 | C11H16N4O4 |
| 4849057 | 4849 | D06 | 284356 | 272 | C14H12N2O4 |
| 4849057 | 4849 | D07 | 622640 | 279 | C8H4F3N3O5 |
| 4849057 | 4849 | D08 | 639754 | 284 | C15H16N4O2 |
| 4849057 | 4849 | D09 | 658144 | 274 | C14H14N2O4 |
| 4849057 | 4849 | D10 | 41809 | 286 | C4H6O4S.2Li.Sb |
| 4849057 | 4849 | D11 | 224117 | 295 | C14H21N3O4 |
| 4849057 | 4849 | E02 | 33006 | 268 | C15H12N2OS |
| 4849057 | 4849 | E03 | 74420 | 273 | C11H13ClN4.ClH |
| 4849057 | 4849 | E04 | 126771 | 283.11 | C13H8Cl2O3 |
| 4849057 | 4849 | E05 | 175493 | 269 | C9H11FeNOS2 |
| 4849057 | 4849 | E06 | 308847 | 283 | C16H17N3O2 |
| 4849057 | 4849 | E07 | 622684 | 275 | C11H14N2O2S.ClH |
| 4849057 | 4849 | E08 | 640391 | 271 | C11H22N2O.2ClH |
| 4849057 | 4849 | E09 | 687667 | 278 | C19H18O2 |
| 4849057 | 4849 | E10 | 45575 | 298 | C20H14N2O |
| 4849057 | 4849 | E11 | 233872 | 299 | C12H17N3O4S |
| 4849057 | 4849 | F02 | 35866 | 271 | C13H13N5S |
| 4849057 | 4849 | F03 | 76747 | 272 | C19H16N2 |
| 4849057 | 4849 | F04 | 129943 | 268.27 | C11H16N4O4 |
| 4849057 | 4849 | F05 | 185065 | 273 | C12H11N5OS |
| 4849057 | 4849 | F06 | 404241 | 267 | C10H13N5O4 |
| 4849057 | 4849 | F07 | 625641 | 277 | C14H13ClN2O2 |
| 4849057 | 4849 | F08 | 640624 | 272 | C14H16N4S |
| 4849057 | 4849 | F09 | 693632 | 269.32 | C13H11N5S |
| 4849057 | 4849 | F10 | 63701 | 291 | C12H13N5O4 |
| 4849057 | 4849 | F11 | 267033 | 292 | C17H24O4 |
| 4849057 | 4849 | G02 | 49660 | 273 | C11H17N4S.Cl |
| 4849057 | 4849 | G03 | 80396 | 272 | C12H7F3O2S |
| 4849057 | 4849 | G04 | 139109 | 282 | C16H14N2O3 |
| 4849057 | 4849 | G05 | 191392 | 272 | C16H14ClNO |
| 4849057 | 4849 | G06 | 406021 | 284 | C10H12N4O4S |
| 4849057 | 4849 | G07 | 627708 | 277 | C13H11NO4S |
| 4849057 | 4849 | G08 | 643174 | 280 | C18H16O3 |
| 4849057 | 4849 | G09 | 697923 | 267 | C11H9NO5S |
| 4849057 | 4849 | G10 | 79456 | 286 | C15H14N2O4 |
| 4849057 | 4849 | G11 | 292684 | 294 | C11H20ClN3O2S |
| 4849057 | 4849 | H02 | 63878 | 280 | C9H13N3O5.ClH |
| 4849057 | 4849 | H03 | 82116 | 276 | C8H16N6OS2 |
| 4849057 | 4849 | H04 | 145669 | 274 | C18H14N2O |
| 4849057 | 4849 | H05 | 191393 | 272 | C16H14ClNO |
| 4849057 | 4849 | H06 | 408120 | 270 | C15H10O5 |
| 4849057 | 4849 | H07 | 632233 | 285 | C16H15NO2S |
| 4849057 | 4849 | H08 | 643186 | 283 | C20H13NO |
| 4849057 | 4849 | H09 | 12825 | 300 | C15H10BrNO |
| 4849057 | 4849 | H10 | 95848 | 300 | C17H16O5 |
| 4849057 | 4849 | H11 | 349155 | 291 | C14H13NO6 |
| 4850057 | 4850 | A02 | 407010 | 286 | C15H10O6 |
| 4850057 | 4850 | A03 | 631583 | 299 | C19H13N3O |
| 4850057 | 4850 | A04 | 650573 | 288 | C18H12N2O2 |
| 4850057 | 4850 | A05 | 38721 | 320.05 | C14H10Cl4 |
| 4850057 | 4850 | A06 | 104801 | 308 | C11H9BrO4.Na |
| 4850057 | 4850 | A07 | 208913 | 308 | C17H28N2O3 |
| 4850057 | 4850 | A08 | 616232 | 308 | C6H12Br2O4 |
| 4850057 | 4850 | A09 | 631152 | 303 | C11H9N7O2S |
| 4850057 | 4850 | A10 | 1027 | 322.82 | C5H7Br3O |
| 4850057 | 4850 | A11 | 634224 | 274 | C17H10N2O2 |
| 4850057 | 4850 | B02 | 407335 | 288 | C11H13FN2O6 |
| 4850057 | 4850 | B03 | 634863 | 292 | C10H12O6S2 |
| 4850057 | 4850 | B04 | 652287 | 292.37 | C14H12O3S2 |
| 4850057 | 4850 | B05 | 59269 | 317 | C19H40O3 |
| 4850057 | 4850 | B06 | 106995 | 314.25 | C15H10N2O6 |
| 4850057 | 4850 | B07 | 224131 | 301 | C6H10NO8P.2Na |
| 4850057 | 4850 | B08 | 618332 | 316 | C10H4Br2O2 |
| 4850057 | 4850 | B09 | 631160 | 317 | C12H11N7O2S |
| 4850057 | 4850 | B10 | 18891 | 334 | C21H22N2S |
| 4850057 | 4850 | B11 | 666168 | 290 | C15H10N6O |
| 4850057 | 4850 | C02 | 610744 | 300 | C15H10ClN3O2 |
| 4850057 | 4850 | C03 | 635140 | 297 | C12H9ClN2O3S |
| 4850057 | 4850 | C04 | 658709 | 297.31 | C17H15NO4 |
| 4850057 | 4850 | C05 | 65346 | 309 | C12H15N5O5 |
| 4850057 | 4850 | C06 | 128305 | 314 | C17H14O6 |
| 4850057 | 4850 | C07 | 266535 | 307.26 | C14H13NO7 |
| 4850057 | 4850 | C08 | 619165 | 313 | C19H28N2Si |
| 4850057 | 4850 | C09 | 633001 | 308 | C12H8N2O6S |
| 4850057 | 4850 | C10 | 36693 | 332.44 | C20H28O4 |
| 4850057 | 4850 | C11 | 118732 | 310 | C16H14N4O3 |
| 4850057 | 4850 | D02 | 611750 | 289 | C12H17BrO3 |
| 4850057 | 4850 | D03 | 636126 | 295 | C18H17NO3 |
| 4850057 | 4850 | D04 | 659997 | 299 | C13H11ClO6 |
| 4850057 | 4850 | D05 | 68093 | 318 | C10H8N2O2S2Zn |
| 4850057 | 4850 | D06 | 147340 | 302 | C14H19NO4.ClH |
| 4850057 | 4850 | D07 | 267461 | 302.28 | C16H14O6 |
| 4850057 | 4850 | D08 | 620277 | 320 | C19H17FN4 |
| 4850057 | 4850 | D09 | 637914 | 314 | C16H14N2O3S |
| 4850057 | 4850 | D10 | 47147 | 323.44 | C20H25N3O |
| 4850057 | 4850 | D11 | 4810 | 327 | C14H9N5O5 |
| 4850057 | 4850 | E02 | 622616 | 292 | C15H16O6 |
| 4850057 | 4850 | E03 | 636132 | 292 | C18H16N2O2 |
| 4850057 | 4850 | E04 | 662553 | 289 | C18H15N3O |
| 4850057 | 4850 | E05 | 78365 | 317.82 | C17H20ClN3O |
| 4850057 | 4850 | E06 | 154020 | 320 | C13H16N6O4 |
| 4850057 | 4850 | E07 | 328477 | 307 | C14H11ClN2O4 |
| 4850057 | 4850 | E08 | 622732 | 309 | C17H13ClN4 |
| 4850057 | 4850 | E09 | 664327 | 307 | C11H15F6NO2 |
| 4850057 | 4850 | E10 | 65937 | 322 | C15H9Cl2NO3 |
| 4850057 | 4850 | E11 | 28002 | 280 | C18H14N.Cl |
| 4850057 | 4850 | F02 | 625487 | 288 | C15H10F2N2S |
| 4850057 | 4850 | F03 | 636786 | 290 | C19H14O3 |
| 4850057 | 4850 | F04 | 680516 | 290 | C18H18N4 |
| 4850057 | 4850 | F05 | 79451 | 317 | C14H11N3O6 |
| 4850057 | 4850 | F06 | 168415 | 309 | C13H9F6NO |
| 4850057 | 4850 | F07 | 329279 | 308 | C13H7Cl2N3O2 |
| 4850057 | 4850 | F08 | 625483 | 305 | C15H10ClFN2S |
| 4850057 | 4850 | F09 | 667467 | 308 | C17H12N2S2 |
| 4850057 | 4850 | F10 | 80756 | 321 | C20H19NO3 |
| 4850057 | 4850 | F11 | 60309 | 286 | C18H10N2O2 |
| 4850057 | 4850 | G02 | 629301 | 296 | C18H8N4O |
| 4850057 | 4850 | G03 | 643175 | 294 | C19H18O3 |
| 4850057 | 4850 | G04 | 25149 | 302 | C20H30O2 |
| 4850057 | 4850 | G05 | 82025 | 317 | C14H11N3O6 |
| 4850057 | 4850 | G06 | 175636 | 309 | C18H13ClN2O |
| 4850057 | 4850 | G07 | 330770 | 311 | C16H17N5O2 |
| 4850057 | 4850 | G08 | 625590 | 315 | C22H21NO |
| 4850057 | 4850 | G09 | 680506 | 301 | C18H23NO3 |
| 4850057 | 4850 | G10 | 93739 | 338 | C20H19N3.ClH |
| 4850057 | 4850 | G11 | 338259 | 302 | C20H19N3.ClH |
| 4850057 | 4850 | H02 | 630374 | 295 | C16H13N3O3 |
| 4850057 | 4850 | H03 | 643774 | 290 | C17H26N2O2 |
| 4850057 | 4850 | H04 | 34757 | 313 | C17H15NO5 |
| 4850057 | 4850 | H05 | 92510 | 310 | C13H19Cl3N2 |
| 4850057 | 4850 | H06 | 202000 | 308 | C14H7Cl2NO3 |
| 4850057 | 4850 | H07 | 352890 | 302.72 | C11H14N4O4.ClH |
| 4850057 | 4850 | H08 | 629659 | 307 | C17H13N3OS |
| 4850057 | 4850 | H09 | 682864 | 310 | C18H16ClN3 |
| 4850057 | 4850 | H10 | 106408 | 329 | C17H19N3O4 |
| 4850057 | 4850 | H11 | 651079 | 315 | C16H17N3O4 |
| 4851057 | 4851 | A02 | 118735 | 331 | C15H11ClN4O3 |
| 4851057 | 4851 | A03 | 299187 | 326.35 | C18H18N2O4 |
| 4851057 | 4851 | A04 | 360861 | 332 | C21H32O3 |
| 4851057 | 4851 | A05 | 624161 | 326 | C4H8O6S4.2Na |
| 4851057 | 4851 | A06 | 635337 | 337 | C18H15N3O4 |
| 4851057 | 4851 | A07 | 658388 | 336 | C18H25N3O.ClH |
| 4851057 | 4851 | A08 | 61811 | 354 | C22H30N2O2 |
| 4851057 | 4851 | A09 | 241509 | 357 | C15H23N3O5S |
| 4851057 | 4851 | A10 | 383468 | 350 | C20H30O5 |
| 4851057 | 4851 | A11 | 637680 | 342 | C18H18N2O3S |
| 4851057 | 4851 | B02 | 140911 | 332 | C14H8N2O2S3 |
| 4851057 | 4851 | B03 | 305782 | 336 | C15H11F3N4S |
| 4851057 | 4851 | B04 | 376791 | 338 | C16H26N4O4 |
| 4851057 | 4851 | B05 | 624358 | 322 | C11H14Br2O |
| 4851057 | 4851 | B06 | 636084 | 335 | C18H19ClO4 |
| 4851057 | 4851 | B07 | 664329 | 336 | C11H8ClF6NO2 |
| 4851057 | 4851 | B08 | 99016 | 346 | C22H26N4 |
| 4851057 | 4851 | B09 | 257473 | 345 | C22H17ClN2 |
| 4851057 | 4851 | B10 | 600305 | 352 | C20H24N4O2 |
| 4851057 | 4851 | B11 | 642649 | 359 | C17H11ClN2O5 |
| 4851057 | 4851 | C02 | 204985 | 324 | C18H12O6 |
| 4851057 | 4851 | C03 | 321803 | 329 | C19H23NO4 |
| 4851057 | 4851 | C04 | 603108 | 336 | C16H15ClFN3O2 |
| 4851057 | 4851 | C05 | 632536 | 323.48 | C21H29N3 |
| 4851057 | 4851 | C06 | 637578 | 325 | C20H15N5 |
| 4851057 | 4851 | C07 | 5890 | 344 | C18H16O7 |
| 4851057 | 4851 | C08 | 123390 | 352 | C20H18NO5 |
| 4851057 | 4851 | C09 | 313981 | 351 | C6H15AuClP |
| 4851057 | 4851 | C10 | 616355 | 350 | C16H27N5O2Si |
| 4851057 | 4851 | C11 | 643162 | 356 | C19H18BrNO |
| 4851057 | 4851 | D02 | 224124 | 336 | C14H12N2O8 |
| 4851057 | 4851 | D03 | 335142 | 326 | C18H15N3O.ClH |
| 4851057 | 4851 | D04 | 621094 | 325 | C16H12FN5O2 |
| 4851057 | 4851 | D05 | 632839 | 340 | C21H21NO.ClH |
| 4851057 | 4851 | D06 | 643164 | 330 | C17H16BrNO |
| 4851057 | 4851 | D07 | 11926 | 341 | C17H11NO7 |
| 4851057 | 4851 | D08 | 135996 | 353 | C15H16N2OS.BrH |
| 4851057 | 4851 | D09 | 316157 | 342 | C18H18N2O5 |
| 4851057 | 4851 | D10 | 620358 | 346 | C21H30O4 |
| 4851057 | 4851 | D11 | 643163 | 342 | C18H16BrNO |
| 4851057 | 4851 | E02 | 235082 | 337 | C20H14N2O2.Na |
| 4851057 | 4851 | E03 | 337612 | 337 | C23H15NO2 |
| 4851057 | 4851 | E04 | 622608 | 322.24 | C9H11N3OS2.BrH |
| 4851057 | 4851 | E05 | 632841 | 329 | C22H19NO2 |
| 4851057 | 4851 | E06 | 645617 | 329 | C11H12N4S4 |
| 4851057 | 4851 | E07 | 20514 | 343 | C20H29N3O2 |
| 4851057 | 4851 | E08 | 143648 | 346 | C12H15N5O5.ClH |
| 4851057 | 4851 | E09 | 320864 | 350 | C15H9Cl2N3O3 |
| 4851057 | 4851 | E10 | 631529 | 341 | C17H9ClN2O2S |
| 4851057 | 4851 | E11 | 646189 | 350 | C17H21NO2Se |
| 4851057 | 4851 | F02 | 282752 | 334 | C20H30O4 |
| 4851057 | 4851 | F03 | 338720 | 325 | C8H6Cl5NO2 |
| 4851057 | 4851 | F04 | 622690 | 340 | C10H13N3S2.ClHO4 |
| 4851057 | 4851 | F05 | 634658 | 324 | C18H16N2O4 |
| 4851057 | 4851 | F06 | 648422 | 328 | C19H20O5 |
| 4851057 | 4851 | F07 | 26040 | 341.88 | C21H24ClNO |
| 4851057 | 4851 | F08 | 157930 | 341 | C21H21S.Cl |
| 4851057 | 4851 | F09 | 323241 | 349.34 | C16H22N4Se |
| 4851057 | 4851 | F10 | 633209 | 355 | C13H8Cl2N4O2S |
| 4851057 | 4851 | F11 | 646200 | 346 | C14H21NO4Se |
| 4851057 | 4851 | G02 | 294577 | 332 | C20H28O4 |
| 4851057 | 4851 | G03 | 339004 | 335 | C14H11ClN4O2S |
| 4851057 | 4851 | G04 | 623135 | 332 | C22H17FO2 |
| 4851057 | 4851 | G05 | 635121 | 325 | C17H15N3O2S |
| 4851057 | 4851 | G06 | 651080 | 336 | C15H14ClN3O4 |
| 4851057 | 4851 | G07 | 44690 | 359 | C21H27N2O.Cl |
| 4851057 | 4851 | G08 | 173905 | 353 | C18H25ClN2O3 |
| 4851057 | 4851 | G09 | 376265 | 359.53 | C19H29N5S |
| 4851057 | 4851 | G10 | 634232 | 344 | C23H20O3 |
| 4851057 | 4851 | G11 | 647613 | 358 | C22H18N2O3 |
| 4851057 | 4851 | H02 | 294961 | 328 | C16H10BrNO2 |
| 4851057 | 4851 | H03 | 349156 | 325 | C14H15NO8 |
| 4851057 | 4851 | H04 | 623637 | 324 | C19H16O5 |
| 4851057 | 4851 | H05 | 635306 | 334 | C16H9Cl2NO3 |
| 4851057 | 4851 | H06 | 658285 | 338 | C15H12ClNO2S2 |
| 4851057 | 4851 | H07 | 53908 | 342 | C19H18O6 |
| 4851057 | 4851 | H08 | 178249 | 354 | C19H14O7 |
| 4851057 | 4851 | H09 | 382766 | 354 | C18H27NO.BrH |
| 4851057 | 4851 | H10 | 634396 | 347 | C17H15ClN2O2S |
| 4851057 | 4851 | H11 | 651084 | 347 | C16H17N3O6 |
| 4852057 | 4852 | A02 | 668270 | 348 | C21H16O5 |
| 4852057 | 4852 | A03 | 26273 | 374 | C17H18N4O4S |
| 4852057 | 4852 | A04 | 157389 | 372 | C22H30ClN3 |
| 4852057 | 4852 | A05 | 284751 | 364 | C10H11ClN5O6P |
| 4852057 | 4852 | A06 | 614826 | 361.4 | C22H19NO4 |
| 4852057 | 4852 | A07 | 635448 | 365 | C10H13BrCuN4S |
| 4852057 | 4852 | A08 | 757 | 399 | C22H25NO6 |
| 4852057 | 4852 | A09 | 146397 | 384 | C21H18NO4.Cl |
| 4852057 | 4852 | A10 | 302358 | 390 | C15H18NOS2.HO4S |
| 4852057 | 4852 | A11 | 621486 | 379 | C20H17N3O5 |
| 4852057 | 4852 | B02 | 671424 | 358 | C20H14N4O3 |
| 4852057 | 4852 | B03 | 32982 | 368.39 | C21H20O6 |
| 4852057 | 4852 | B04 | 163088 | 372 | C20H18NO4.Cl |
| 4852057 | 4852 | B05 | 302979 | 374 | C22H30O5 |
| 4852057 | 4852 | B06 | 617540 | 361 | C24H40O2 |
| 4852057 | 4852 | B07 | 635542 | 370 | C16H14N6O5 |
| 4852057 | 4852 | B08 | 14974 | 396 | C20H28O8 |
| 4852057 | 4852 | B09 | 166381 | 384 | C21H25N3O2S |
| 4852057 | 4852 | B10 | 305884 | 382 | C20H19N5O.ClH |
| 4852057 | 4852 | B11 | 634568 | 366 | C17H10N4O4S |
| 4852057 | 4852 | C02 | 672904 | 354 | C19H41ClN.Cl |
| 4852057 | 4852 | C03 | 36437 | 376 | C22H32O5 |
| 4852057 | 4852 | C04 | 174163 | 363 | C10H7ClHg |
| 4852057 | 4852 | C05 | 327697 | 361 | C17H14Cl2N4O |
| 4852057 | 4852 | C06 | 620279 | 374 | C22H22N4O2 |
| 4852057 | 4852 | C07 | 640580 | 375 | C18H12Cl2N2OS |
| 4852057 | 4852 | C08 | 24817 | 400 | C21H20O8 |
| 4852057 | 4852 | C09 | 169600 | 395 | C19H23BrO4 |
| 4852057 | 4852 | C10 | 322069 | 396 | C20H29NO2.BrH |
| 4852057 | 4852 | C11 | 62791 | 397 | C28H44O |
| 4852057 | 4852 | D02 | 678932 | 346 | C17H19N5O.ClH |
| 4852057 | 4852 | D03 | 83265 | 363.47 | C22H21NO2S |
| 4852057 | 4852 | D04 | 182986 | 364 | C16H20N4O6 |
| 4852057 | 4852 | D05 | 343513 | 373 | C22H25F2NO2 |
| 4852057 | 4852 | D06 | 626734 | 368 | C21H24N2O4 |
| 4852057 | 4852 | D07 | 645033 | 372 | C18H15Cl2N5 |
| 4852057 | 4852 | D08 | 77021 | 396 | C21H26F2O5 |
| 4852057 | 4852 | D09 | 172946 | 385 | C21H23NO6 |
| 4852057 | 4852 | D10 | 403148 | 398 | C22H22O7 |
| 4852057 | 4852 | D11 | 689872 | 360 | C20H16N4O3 |
| 4852057 | 4852 | E02 | 684845 | 352 | C14H16F4N2O4 |
| 4852057 | 4852 | E03 | 97911 | 373 | C17H18N4O2.HNO3 |
| 4852057 | 4852 | E04 | 185056 | 365 | C15H13ClN4O3S |
| 4852057 | 4852 | E05 | 352876 | 376 | C20H24O7 |
| 4852057 | 4852 | E06 | 629713 | 362 | C24H14N2O2 |
| 4852057 | 4852 | E07 | 647363 | 372 | C20H20O7 |
| 4852057 | 4852 | E08 | 98542 | 381 | C22H23NO5 |
| 4852057 | 4852 | E09 | 249992 | 393 | C21H19N3O3S |
| 4852057 | 4852 | E10 | 603577 | 384 | C4H14Cl2N2PtSi |
| 4852057 | 4852 | E11 | 157004 | 340 | C18H16N2O5 |
| 4852057 | 4852 | F02 | 1011 | 370.41 | C23H18N2O3 |
| 4852057 | 4852 | F03 | 115538 | 366 | C21H18O6 |
| 4852057 | 4852 | F04 | 211500 | 362 | C19H22O7 |
| 4852057 | 4852 | F05 | 407806 | 374.52 | C23H34O4 |
| 4852057 | 4852 | F06 | 629971 | 363 | C20H17N3O4 |
| 4852057 | 4852 | F07 | 681730 | 366.46 | C24H22N4 |
| 4852057 | 4852 | F08 | 104117 | 389 | C20H18Cl2N2O2 |
| 4852057 | 4852 | F09 | 262665 | 385 | C24H19NO4 |
| 4852057 | 4852 | F10 | 293927 | 338 | C18H10O3S2 |
| 4852057 | 4852 | F11 | 329277 | 378 | C17H13Cl2N3O3 |
| 4852057 | 4852 | G02 | 14574 | 377 | C20H24N2OS.ClH |
| 4852057 | 4852 | G03 | 123115 | 376.41 | C18H24N4O5 |
| 4852057 | 4852 | G04 | 241906 | 378 | C23H22O5 |
| 4852057 | 4852 | G05 | 601101 | 373 | C21H24N2O2.ClH |
| 4852057 | 4852 | G06 | 631521 | 375 | C17H8Cl2N2O2S |
| 4852057 | 4852 | G07 | 681741 | 368 | C25H24N2O |
| 4852057 | 4852 | G08 | 126727 | 398 | C22H22O7 |
| 4852057 | 4852 | G09 | 268986 | 394 | C21H20N3.Br |
| 4852057 | 4852 | G10 | 69852 | 348 | C10H26N4.4ClH |
| 4852057 | 4852 | G11 | 163443 | 394.47 | C26H22N2O2 |
| 4852057 | 4852 | H02 | 24113 | 369 | C21H21ClN2S |
| 4852057 | 4852 | H03 | 140377 | 370 | C21H22O6 |
| 4852057 | 4852 | H04 | 265459 | 380 | C6H14Cl2N2Pt |
| 4852057 | 4852 | H05 | 604535 | 369 | C20H19NO4S |
| 4852057 | 4852 | H06 | 635437 | 371 | C16H10Cl3NO3 |
| 4852057 | 4852 | H07 | 377 | 384.43 | C25H20O4 |
| 4852057 | 4852 | H08 | 136037 | 383 | C15H16O5.C7H9N |
| 4852057 | 4852 | H09 | 281245 | 391 | C23H34O5 |
| 4852057 | 4852 | H10 | 314622 | 365 | C20H15NO6 |
| 4852057 | 4852 | H11 | 371846 | 398 | C24H22N4O2 |
| 4853057 | 4853 | A02 | 624169 | 393 | C12H20N2S4.2ClH |
| 4853057 | 4853 | A03 | 657456 | 399 | C17H11BrN4OS |
| 4853057 | 4853 | A04 | 2979 | 444 | C20H30Cl4O2 |
| 4853057 | 4853 | A05 | 33410 | 462 | C27H27NO6 |
| 4853057 | 4853 | A06 | 51812 | 451 | C21H22N2O7.ClH |
| 4853057 | 4853 | A07 | 84074 | 464 | C21H21BrP.Br |
| 4853057 | 4853 | A08 | 138429 | 453 | C21H31N3O8 |
| 4853057 | 4853 | A09 | 196524 | 428 | C22H20O9 |
| 4853057 | 4853 | A10 | 293015 | 416 | C23H25NO4.ClH |
| 4853057 | 4853 | A11 | 351306 | 435 | C25H26N2O3S |
| 4853057 | 4853 | B02 | 634473 | 381 | C21H19NO6 |
| 4853057 | 4853 | B03 | 678917 | 391 | C19H22N4O3.ClH |
| 4853057 | 4853 | B04 | 4644 | 443 | C30H50O2 |
| 4853057 | 4853 | B05 | 35489 | 475 | C25H18NO.I |
| 4853057 | 4853 | B06 | 56737 | 443 | C22H26N4O4S |
| 4853057 | 4853 | B07 | 85561 | 462.39 | C20H13N3O7S.Na |
| 4853057 | 4853 | B08 | 142982 | 453 | C20H24N2O2S.CH4O3S |
| 4853057 | 4853 | B09 | 211489 | 456 | C27H20O7 |
| 4853057 | 4853 | B10 | 311153 | 433.55 | C24H28N3O.C2H3O2 |
| 4853057 | 4853 | B11 | 374898 | 411 | C25H21N3O3 |
| 4853057 | 4853 | C02 | 635321 | 392 | C23H18ClNO3 |
| 4853057 | 4853 | C03 | 680509 | 388 | C21H25N3O2.ClH |
| 4853057 | 4853 | C04 | 11930 | 418 | C17H20INO.ClH |
| 4853057 | 4853 | C05 | 35949 | 470 | C28H27N3O2S |
| 4853057 | 4853 | C06 | 66300 | 415 | C10H14O4S.C9H20N4 |
| 4853057 | 4853 | C07 | 85700 | 454.31 | C22H19N2O.I |
| 4853057 | 4853 | C08 | 149765 | 455 | C15H21N3O.2H3O4P |
| 4853057 | 4853 | C09 | 256927 | 418 | C6H20Cl2N2O2Pt |
| 4853057 | 4853 | C10 | 329696 | 418 | C23H30O7 |
| 4853057 | 4853 | C11 | 600300 | 472 | C8H26Cl2N2PtSi2 |
| 4853057 | 4853 | D02 | 635326 | 381.17 | C16H10Cl2N2O5 |
| 4853057 | 4853 | D03 | 684480 | 397 | C25H17ClN2O |
| 4853057 | 4853 | D04 | 14229 | 472.88 | C23H30ClN3O.2ClH |
| 4853057 | 4853 | D05 | 39863 | 417 | C28H36N2O |
| 4853057 | 4853 | D06 | 67580 | 432 | C22H24O9 |
| 4853057 | 4853 | D07 | 98904 | 412 | C21H14NS.ClO4 |
| 4853057 | 4853 | D08 | 167410 | 448 | C21H20O11 |
| 4853057 | 4853 | D09 | 265473 | 426 | C21H35N3O4S |
| 4853057 | 4853 | D10 | 330515 | 402 | C23H30O6 |
| 4853057 | 4853 | D11 | 603578 | 455 | C4H14Cl4N2PtSi |
| 4853057 | 4853 | E02 | 635328 | 386 | C20H13Cl2NO3 |
| 4853057 | 4853 | E03 | 705330 | 385 | C21H23NO4S |
| 4853057 | 4853 | E04 | 19857 | 420 | C27H33NO3 |
| 4853057 | 4853 | E05 | 40666 | 423.87 | C17H18ClN5O4S |
| 4853057 | 4853 | E06 | 67690 | 405 | C27H19NO3 |
| 4853057 | 4853 | E07 | 100856 | 409 | C19H20N4O2.2ClH |
| 4853057 | 4853 | E08 | 168597 | 414 | C12H27ClPb |
| 4853057 | 4853 | E09 | 267700 | 454 | C21H25Cl2N3O4 |
| 4853057 | 4853 | E10 | 331757 | 429 | C24H28N2O3.ClH |
| 4853057 | 4853 | E11 | 618261 | 407 | C20H25NO8 |
| 4853057 | 4853 | F02 | 635435 | 391.85 | C23H18ClNO3 |
| 4853057 | 4853 | F03 | 534 | 437 | C16H12HgO2 |
| 4853057 | 4853 | F04 | 24048 | 442 | C21H18N2O.HI |
| 4853057 | 4853 | F05 | 43321 | 420 | C21H24O9 |
| 4853057 | 4853 | F06 | 71300 | 402 | C21H22O8 |
| 4853057 | 4853 | F07 | 104129 | 455 | C21H25Cl2N5.ClH |
| 4853057 | 4853 | F08 | 170984 | 461.54 | C28H29F2N3O |
| 4853057 | 4853 | F09 | 267712 | 453 | C28H36O5 |
| 4853057 | 4853 | F10 | 337766 | 471 | C22H22N8.2ClH |
| 4853057 | 4853 | F11 | 620280 | 412 | C24H24N6O |
| 4853057 | 4853 | G02 | 635438 | 381 | C16H10Cl2N2O5 |
| 4853057 | 4853 | G03 | 740 | 454.44 | C20H22N8O5 |
| 4853057 | 4853 | G04 | 24818 | 414 | C22H22O8 |
| 4853057 | 4853 | G05 | 47438 | 419 | C24H31FO5 |
| 4853057 | 4853 | G06 | 71669 | 412 | C19H14F6N4 |
| 4853057 | 4853 | G07 | 109444 | 423 | C27H34N2.ClH |
| 4853057 | 4853 | G08 | 173904 | 445 | C24H29ClN2O4 |
| 4853057 | 4853 | G09 | 273829 | 443 | C27H34N6 |
| 4853057 | 4853 | G10 | 345081 | 405 | C15H15Cl2N2O5P |
| 4853057 | 4853 | G11 | 623746 | 449 | C20H20N2O6S2 |
| 4853057 | 4853 | H02 | 644735 | 395 | C18H13N5O6 |
| 4853057 | 4853 | H03 | 2186 | 431 | C28H30O4 |
| 4853057 | 4853 | H04 | 24819 | 414 | C22H22O8 |
| 4853057 | 4853 | H05 | 48151 | 424 | C28H41NO2 |
| 4853057 | 4853 | H06 | 73413 | 462.39 | C20H13N3O7S.Na |
| 4853057 | 4853 | H07 | 113090 | 457 | C30H48O3 |
| 4853057 | 4853 | H08 | 174176 | 407 | C13H18HgO2 |
| 4853057 | 4853 | H09 | 292663 | 422 | C22H30O8 |
| 4853057 | 4853 | H10 | 349644 | 426 | C22H26N4O5 |
| 4853057 | 4853 | H11 | 624158 | 429 | C18H20O4S4 |
| 4854057 | 4854 | A02 | 624947 | 416 | C19H15Cl2N5O2 |
| 4854057 | 4854 | A03 | 635366 | 405 | C21H25ClN2S2 |
| 4854057 | 4854 | A04 | 641253 | 473 | C26H17ClN2O5 |
| 4854057 | 4854 | A05 | 657446 | 433 | C19H13N2O4S2.Cl |
| 4854057 | 4854 | A06 | 668260 | 453 | C22H17BrN2O4 |
| 4854057 | 4854 | A07 | 689857 | 408 | C25H29NO4 |
| 4854057 | 4854 | A08 | 32992 | 548 | C27H33NO11 |
| 4854057 | 4854 | A09 | 96932 | 506 | C22H23N2S2.I |
| 4854057 | 4854 | A10 | 15623 | 464 | C13H19O9Sb.Na |
| 4854057 | 4854 | A11 | 643148 | 425 | C25H15NO6 |
| 4854057 | 4854 | B02 | 626120 | 413 | C20H22Cl2O5 |
| 4854057 | 4854 | B03 | 635436 | 405 | C16H9Cl4NO3 |
| 4854057 | 4854 | B04 | 641607 | 403 | C24H21NO5 |
| 4854057 | 4854 | B05 | 657598 | 448 | C18H10N3O5S2.Cl |
| 4854057 | 4854 | B06 | 670224 | 423 | C21H33Cl2NO.ClH |
| 4854057 | 4854 | B07 | 693172 | 414 | C25H45FN.Cl |
| 4854057 | 4854 | B08 | 34391 | 480 | C25H25N2.I |
| 4854057 | 4854 | B09 | 102815 | 599.59 | C30H33NO12 |
| 4854057 | 4854 | B10 | 40341 | 467 | C24H16Cl2N2O4 |
| 4854057 | 4854 | B11 | 10010 | 573.6 | C31H40N4O2.2ClH |
| 4854057 | 4854 | C02 | 627666 | 454 | C27H22N2O5 |
| 4854057 | 4854 | C03 | 635824 | 420 | C24H24N2O5 |
| 4854057 | 4854 | C04 | 642048 | 432 | C21H25N3O3S2 |
| 4854057 | 4854 | C05 | 657603 | 446 | C19H16N3O3S.Br |
| 4854057 | 4854 | C06 | 670225 | 448 | C22H37NO.C4H4O4 |
| 4854057 | 4854 | C07 | 697443 | 426 | C25H27NO3.HCl |
| 4854057 | 4854 | C08 | 36354 | 507 | C20H23NO5.C4H6O6 |
| 4854057 | 4854 | C09 | 90487 | 427 | C30H50O |
| 4854057 | 4854 | C10 | 164909 | 451 | C23H24O2Sn |
| 4854057 | 4854 | C11 | 623051 | 383 | C19H11ClN2O5 |
| 4854057 | 4854 | D02 | 629738 | 453 | C30H32N2O2 |
| 4854057 | 4854 | D03 | 635833 | 412 | C21H20N2O5S |
| 4854057 | 4854 | D04 | 643599 | 434 | C24H23N3O3S |
| 4854057 | 4854 | D05 | 658293 | 413 | C16H13N2O5S2.Cl |
| 4854057 | 4854 | D06 | 670226 | 452 | C21H34FNO.C4H4O4 |
| 4854057 | 4854 | D07 | 698031 | 459 | C24H22N6O2S |
| 4854057 | 4854 | D08 | 45383 | 506.47 | C25H22N4O8 |
| 4854057 | 4854 | D09 | 106997 | 422 | C19H36N2O5.1/2H2O4S |
| 4854057 | 4854 | D10 | 183359 | 462 | C17H29NO2S2Sn |
| 4854057 | 4854 | D11 | 623059 | 389 | C18H13ClN2O4S |
| 4854057 | 4854 | E02 | 634503 | 407 | C23H19ClN2O3 |
| 4854057 | 4854 | E03 | 637916 | 450 | C22H16ClN5O4 |
| 4854057 | 4854 | E04 | 647418 | 456 | C26H33N3O2.ClH |
| 4854057 | 4854 | E05 | 658494 | 450 | C23H16ClN3O5 |
| 4854057 | 4854 | E06 | 670229 | 448 | C22H37NO.C4H4O4 |
| 4854057 | 4854 | E07 | 7521 | 530.66 | C30H42O8 |
| 4854057 | 4854 | E08 | 49451 | 558.71 | C32H46O8 |
| 4854057 | 4854 | E09 | 622586 | 414 | C15H19N5OS4 |
| 4854057 | 4854 | E10 | 282880 | 436 | C23H50NO4P |
| 4854057 | 4854 | E11 | 90829 | 409 | C21H40N2O.2ClH |
| 4854057 | 4854 | F02 | 634926 | 432 | C29H21NOS |
| 4854057 | 4854 | F03 | 638634 | 406 | C28H23NO2 |
| 4854057 | 4854 | F04 | 648419 | 406 | C24H22O6 |
| 4854057 | 4854 | F05 | 659174 | 423 | C9H6Cl2F10O3 |
| 4854057 | 4854 | F06 | 673622 | 429 | C23H27NO7 |
| 4854057 | 4854 | F07 | 7522 | 549 | C30H44O9 |
| 4854057 | 4854 | F08 | 56817 | 519 | C30H30O8 |
| 4854057 | 4854 | F09 | 624206 | 411 | C19H32ClNS2.ClH |
| 4854057 | 4854 | F10 | 376248 | 475 | C31H22O5 |
| 4854057 | 4854 | F11 | 138925 | 454 | C17H34N4O10 |
| 4854057 | 4854 | G02 | 634928 | 468 | C29H29N3O3 |
| 4854057 | 4854 | G03 | 639828 | 463 | C18H13BrClN5O3 |
| 4854057 | 4854 | G04 | 650792 | 438 | C17H27NO2S5 |
| 4854057 | 4854 | G05 | 662825 | 401 | C10H15Br2Cl3 |
| 4854057 | 4854 | G06 | 682769 | 422 | C27H22N2O3 |
| 4854057 | 4854 | G07 | 14975 | 497 | C25H36O10 |
| 4854057 | 4854 | G08 | 79688 | 476 | C23H23Cl2N3O4 |
| 4854057 | 4854 | G09 | 640974 | 442 | C25H15FN2O5 |
| 4854057 | 4854 | G10 | 605583 | 408 | C21H46NO4P |
| 4854057 | 4854 | G11 | 622589 | 428 | C15H17N5O2S4 |
| 4854057 | 4854 | H02 | 635312 | 412.27 | C22H15Cl2NO3 |
| 4854057 | 4854 | H03 | 641228 | 431.41 | C25H18FNO5 |
| 4854057 | 4854 | H04 | 657298 | 426 | C26H31NO2.ClH |
| 4854057 | 4854 | H05 | 664181 | 416 | C11H8F12O3 |
| 4854057 | 4854 | H06 | 689228 | 429 | C23H44O5Si |
| 4854057 | 4854 | H07 | 18298 | 572 | C30H26CuN4O4.2H |
| 4854057 | 4854 | H08 | 93419 | 575 | C28H30O13 |
| 4854057 | 4854 | H09 | 672425 | 397 | C19H14N2O4.HNO3 |
| 4854057 | 4854 | H10 | 637993 | 451 | C22H26N4O2.2ClH |
| 4854057 | 4854 | H11 | 643351 | 444 | C22H24CuN2O4 |
| 4855057 | 4855 | A02 | 118030 | 482 | C27H25NSn |
| 4855057 | 4855 | A03 | 167780 | 488 | C23H35Cl2N3O4 |
| 4855057 | 4855 | A04 | 184403 | 575 | C18H14NO4.2C5H5N.2ClH.Cl |
| 4855057 | 4855 | A05 | 269754 | 549 | C29H40O10 |
| 4855057 | 4855 | A06 | 330516 | 537 | C30H32O9 |
| 4855057 | 4855 | A07 | 617570 | 561 | C23H21ClN5NiO4.Cl |
| 4855057 | 4855 | A08 | 640985 | 478 | C30H23NO5 |
| 4855057 | 4855 | A09 | 653000 | 565 | C21H48N12O6 |
| 4855057 | 4855 | A10 | 670140 | 485 | C22H15Br2NO2 |
| 4855057 | 4855 | A11 | 690634 | 519 | C29H22N6O4 |
| 4855057 | 4855 | B02 | 126728 | 503 | C27H34O9 |
| 4855057 | 4855 | B03 | 169676 | 590 | C24H31F3N4OS.3ClH |
| 4855057 | 4855 | B04 | 240419 | 497 | C20H23Br2N3O2 |
| 4855057 | 4855 | B05 | 285223 | 525 | C13H14N2O4.C13H14N2O4 |
| 4855057 | 4855 | B06 | 330753 | 587 | C31H38O11 |
| 4855057 | 4855 | B07 | 623093 | 587 | C25H17F3N6O2S3 |
| 4855057 | 4855 | B08 | 641233 | 563.95 | C32H18ClNO7 |
| 4855057 | 4855 | B09 | 654259 | 535.78 | C21H19BrN6O4.ClH |
| 4855057 | 4855 | B10 | 671136 | 567.46 | C27H16Cl2N2O4S2 |
| 4855057 | 4855 | B11 | 699479 | 562 | C26H23ClN3.CF3O3S |
| 4855057 | 4855 | C02 | 129414 | 552 | C29H38N2O4.2ClH |
| 4855057 | 4855 | C03 | 172924 | 521 | C26H32O11 |
| 4855057 | 4855 | C04 | 243928 | 504 | C22H21N3O3S.CH4O3S |
| 4855057 | 4855 | C05 | 288010 | 489 | C25H29ClN4O2.ClH |
| 4855057 | 4855 | C06 | 345647 | 546.53 | C30H26O10 |
| 4855057 | 4855 | C07 | 623095 | 487 | C22H16F3N5O3S |
| 4855057 | 4855 | C08 | 641240 | 496 | C28H20N2O7 |
| 4855057 | 4855 | C09 | 657457 | 547 | C22H19BrN4O4S2 |
| 4855057 | 4855 | C10 | 671394 | 566 | C32H31N5O3S |
| 4855057 | 4855 | C11 | 703550 | 492 | C29H29N7O |
| 4855057 | 4855 | D02 | 132791 | 495 | C25H34O10 |
| 4855057 | 4855 | D03 | 174121 | 505 | C24H24N8O5 |
| 4855057 | 4855 | D04 | 245432 | 482 | C17H24ClN3O11 |
| 4855057 | 4855 | D05 | 290205 | 564 | C31H44Cl2N2O3 |
| 4855057 | 4855 | D06 | 354844 | 508.52 | C28H28O9 |
| 4855057 | 4855 | D07 | 635544 | 484 | C19H13Cl3N4O5 |
| 4855057 | 4855 | D08 | 641245 | 553 | C30H17ClN2O7 |
| 4855057 | 4855 | D09 | 657722 | 490.4 | C24H21Cl2NO4S |
| 4855057 | 4855 | D10 | 679524 | 533 | C27H20N2O8S |
| 4855057 | 4855 | D11 | 5159 | 641 | C32H32O14 |
| 4855057 | 4855 | E02 | 133071 | 512.02 | C20H24ClN5O2.C2H6O3S |
| 4855057 | 4855 | E03 | 177365 | 566.46 | C23H23N7O4S.2ClH |
| 4855057 | 4855 | E04 | 255109 | 546 | C28H39N3O8 |
| 4855057 | 4855 | E05 | 305222 | 494 | C30H39NO5 |
| 4855057 | 4855 | E06 | 361813 | 497 | C26H36N6O4 |
| 4855057 | 4855 | E07 | 638646 | 591 | C26H24Cl4N2O3.ClH |
| 4855057 | 4855 | E08 | 641250 | 507 | C26H16Cl2N2O5 |
| 4855057 | 4855 | E09 | 657799 | 491 | C27H38N2O4.ClH |
| 4855057 | 4855 | E10 | 679527 | 479 | C25H22N2O6S |
| 4855057 | 4855 | E11 | 7525 | 693 | C36H52O13 |
| 4855057 | 4855 | F02 | 139105 | 539 | C21H25ClN6O2.C2H6O3S |
| 4855057 | 4855 | F03 | 180973 | 563.65 | C26H29NO.C6H8O7 |
| 4855057 | 4855 | F04 | 258812 | 592 | C29H33NO10.ClH |
| 4855057 | 4855 | F05 | 305819 | 541.33 | C18H20F6N2O.H3O4P |
| 4855057 | 4855 | F06 | 363744 | 513 | C18H20N2O.C10H16O4S |
| 4855057 | 4855 | F07 | 640584 | 516 | C28H19Cl2N3OS |
| 4855057 | 4855 | F08 | 642033 | 477 | C25H28N6O4 |
| 4855057 | 4855 | F09 | 658139 | 505 | C27H15N5O6 |
| 4855057 | 4855 | F10 | 681744 | 555 | C36H34N4O2 |
| 4855057 | 4855 | F11 | 7530 | 872.96 | C42H64O19 |
| 4855057 | 4855 | G02 | 164914 | 487 | C18H29Cl3OSn |
| 4855057 | 4855 | G03 | 181486 | 563 | C35H34N2O5 |
| 4855057 | 4855 | G04 | 268251 | 575.75 | C29H49N7O5 |
| 4855057 | 4855 | G05 | 328166 | 535 | C27H34O11 |
| 4855057 | 4855 | G06 | 363998 | 513 | C24H32N4O4.2ClH |
| 4855057 | 4855 | G07 | 640637 | 513 | C31H24N6S |
| 4855057 | 4855 | G08 | 642040 | 526 | C29H23N3O7 |
| 4855057 | 4855 | G09 | 658350 | 493 | C24H24N6O2S2 |
| 4855057 | 4855 | G10 | 686349 | 509 | C16H12O9S5 |
| 4855057 | 4855 | G11 | 19990 | 770 | C40H51NO14 |
| 4855057 | 4855 | H02 | 165563 | 548.59 | C28H36O11 |
| 4855057 | 4855 | H03 | 184398 | 486 | C25H26O10 |
| 4855057 | 4855 | H04 | 269148 | 542 | C28H31NO10 |
| 4855057 | 4855 | H05 | 330500 | 560.69 | C30H44N2O8 |
| 4855057 | 4855 | H06 | 603624 | 483 | C19H20Cl2N6O3S |
| 4855057 | 4855 | H07 | 640638 | 537 | C24H19Br2N5 |
| 4855057 | 4855 | H08 | 644794 | 536 | C25H14Cl2F3NO5 |
| 4855057 | 4855 | H09 | 659999 | 478 | C18H21BrN2O6.ClH |
| 4855057 | 4855 | H10 | 687330 | 509 | C22H40N6O4Si2 |
| 4855057 | 4855 | H11 | 30916 | 758 | C20H20Hg2O6 |
| 4856057 | 4856 | A02 | 46061 | 644 | C21H24F3N3S.2C4H6O4 |
| 4856057 | 4856 | A03 | 97338 | 623 | C38H42N2O6 |
| 4856057 | 4856 | A04 | 169774 | 751 | C32H40BrN5O5.CH4O3S |
| 4856057 | 4856 | A05 | 281613 | 648 | C29H30ClN3O6S3 |
| 4856057 | 4856 | A06 | 607316 | 827 | C44H50N4O12 |
| 4856057 | 4856 | A07 | 653558 | 605 | C16H34Ge2O4 |
| 4856057 | 4856 | A08 | 67574 | 923.04 | C46H56N4O10.H2O4S |
| 4856057 | 4856 | A09 | 306864 | 1015 | C51H82O20 |
| 4856057 | 4856 | A10 | 154890 | 504 | C22H22NO4.C2H3O5S |
| 4856057 | 4856 | A11 | 292567 | 748 | C40H68O11.Na |
| 4856057 | 4856 | B02 | 52141 | 737 | C40H64O12 |
| 4856057 | 4856 | B03 | 103248 | 712 | C42H53N3O7 |
| 4856057 | 4856 | B04 | 208734 | 812 | C42H53NO15 |
| 4856057 | 4856 | B05 | 304421 | 825 | C46H56N4O10 |
| 4856057 | 4856 | B06 | 619907 | 833 | C14H6I4MnO6 |
| 4856057 | 4856 | B07 | 669356 | 677 | C35H56N6O5.ClH |
| 4856057 | 4856 | B08 | 76027 | 1058 | C39H27N5O15S5.4Na |
| 4856057 | 4856 | B09 | 325319 | 1112 | C57H89N7O15 |
| 4856057 | 4856 | B10 | 173046 | 479 | C16H16O4Pb |
| 4856057 | 4856 | B11 | 675593 | 711 | C28H42N10O4S4 |
| 4856057 | 4856 | C02 | 65104 | 622 | C30H51N7O7 |
| 4856057 | 4856 | C03 | 109350 | 767 | C45H58N4O7 |
| 4856057 | 4856 | C04 | 218439 | 622 | C29H25N7O5.2Cl |
| 4856057 | 4856 | C05 | 328426 | 805 | C40H52O17 |
| 4856057 | 4856 | C06 | 620261 | 809 | C13H16N2O4.4C8H8O2 |
| 4856057 | 4856 | C07 | 676963 | 807 | C34H34N6OS.2C4H4O4 |
| 4856057 | 4856 | C08 | 90636 | 907 | C46H56N4O9.H2O4S |
| 4856057 | 4856 | C09 | 363182 | 1187 | C61H90N2O21 |
| 4856057 | 4856 | C10 | 219734 | 499 | C34H34N4 |
| 4856057 | 4856 | C11 | 677392 | 849 | C40H36N10O10S |
| 4856057 | 4856 | D02 | 68075 | 696.84 | C41H48N2O8 |
| 4856057 | 4856 | D03 | 116693 | 663 | C21H21S.C18H13O8 |
| 4856057 | 4856 | D04 | 243023 | 862 | C42H51NO16.ClH |
| 4856057 | 4856 | D05 | 332598 | 625.76 | C35H47NO9 |
| 4856057 | 4856 | D06 | 624953 | 605 | C36H48O6Si |
| 4856057 | 4856 | D07 | 7532 | 969 | C49H76O19 |
| 4856057 | 4856 | D08 | 107412 | 1110 | C55H59N5O20 |
| 4856057 | 4856 | D09 | 603169 | 1027 | C42H36N8O2.2C7H7O3S |
| 4856057 | 4856 | D10 | 267229 | 586 | C30H35NO11 |
| 4856057 | 4856 | D11 | 166454 | 528 | C30H40N4.2Cl |
| 4856057 | 4856 | E02 | 70845 | 788 | C39H49NO16 |
| 4856057 | 4856 | E03 | 136044 | 701 | C36H48N2O12 |
| 4856057 | 4856 | E04 | 248436 | 654 | C10H13Br2N5O4PtS |
| 4856057 | 4856 | E05 | 337851 | 696 | C40H45N3O6S |
| 4856057 | 4856 | E06 | 625331 | 660 | C18H25N5.3C4H4O4 |
| 4856057 | 4856 | E07 | 24559 | 1085.16 | C52H76O24 |
| 4856057 | 4856 | E08 | 115493 | 977 | C27H44O8.C27H44O7 |
| 4856057 | 4856 | E09 | 638352 | 934.76 | C32H22N6O14S4.4Na |
| 4856057 | 4856 | E10 | 683792 | 582 | C34H40N2O2.2ClH |
| 4856057 | 4856 | E11 | 192965 | 499 | C17H36GeN2.2ClH |
| 4856057 | 4856 | F02 | 73495 | 678 | C22H21NO7.C12H26O4S |
| 4856057 | 4856 | F03 | 145366 | 671.83 | C37H62N2O4.2ClH |
| 4856057 | 4856 | F04 | 265450 | 729.78 | C37H47NO14 |
| 4856057 | 4856 | F05 | 353527 | 692 | C28H25ClN3O2Sn.C6H15N.H |
| 4856057 | 4856 | F06 | 625873 | 640 | C31H33N3O10S |
| 4856057 | 4856 | F07 | 34931 | 932.78 | C34H24N4O14S4.4Na |
| 4856057 | 4856 | F08 | 226080 | 914.18 | C51H79NO13 |
| 4856057 | 4856 | F09 | 640342 | 1020 | C48H44N12O6S2.2Cl |
| 4856057 | 4856 | F10 | 700582 | 548.91 | C22H36N4S6 |
| 4856057 | 4856 | F11 | 260610 | 531 | C34H34N4O2 |
| 4856057 | 4856 | G02 | 76455 | 853 | C46H76O14 |
| 4856057 | 4856 | G03 | 153858 | 692 | C34H46ClN3O10 |
| 4856057 | 4856 | G04 | 268242 | 744 | C41H41NO10.ClH |
| 4856057 | 4856 | G05 | 359463 | 644 | C32H38N6O4.2ClH |
| 4856057 | 4856 | G06 | 640335 | 613 | C26H18Cl2N6O6S |
| 4856057 | 4856 | G07 | 49842 | 909.06 | C46H58N4O9.H2O4S |
| 4856057 | 4856 | G08 | 253995 | 1093 | C40H24EuF12O8.C5H5N.H |
| 4856057 | 4856 | G09 | 139490 | 519 | C21H27N7O6.2Na |
| 4856057 | 4856 | G10 | 70929 | 747 | C41H50N2O11 |
| 4856057 | 4856 | G11 | 276299 | 527 | C10H8N10PtS2 |
| 4856057 | 4856 | H02 | 93135 | 609 | C37H40N2O6 |
| 4856057 | 4856 | H03 | 156215 | 812 | C42H53NO15 |
| 4856057 | 4856 | H04 | 274893 | 738 | C40H44N2O7.2ClH |
| 4856057 | 4856 | H05 | 600681 | 695 | C21H28N2.C20H18O8 |
| 4856057 | 4856 | H06 | 645567 | 690 | C6H5Cl3Sn.C15H35NO6P2 |
| 4856057 | 4856 | H07 | 58514 | 1183 | C57H82O26 |
| 4856057 | 4856 | H08 | 269146 | 1085 | C52H76O24 |
| 4856057 | 4856 | H09 | 324368 | 524 | C27H58NO6P |
| 4856057 | 4856 | H10 | 265211 | 846 | C42H55NO17 |
| 4856057 | 4856 | H11 | 239375 | 724 | C38H45NO13 |
