# Supplementary material for: Novel lactate dehydrogenase inhibitors with in vivo efficacy against Cryptosporidium parvum
Source: PLoS Pathog. 2019 Jul 29;15(7):e1007953. doi: 10.1371/journal.ppat.1007953 (PMC6687188; doi:10.1371/journal.ppat.1007953)
Supplement: S3 Table — (DOC) [file ppat.1007953.s003.doc]

**S3 Table.** **Cytotoxicity IC_50_ values in**

**HCT-8 cells for inhibitors of CpLDH.**

| **COMPOUND** | **CYTOTOXICITY IC_50_ (µM)** |
| --- | --- |
| NSC51148 | 39.65 |
| NSC1771 | 110.11 |
| NSC626433 | 265.03 |
| NSC349438 | 93.90 |
| NSC10447 | 290.28 |
| NSC85561 | 103.29 |
| NSC73413 | 219.80 |
| NSC657799 | 113.90 |
| NSC686349 | 282.94 |
| NSC638352 | 116.49 |
| NSC34931 | 71.08 |
| NSC253995 | 83.05 |
| NSC70925 | 141.90 |
| NSC70929 | 180.85 |
| NSC36354 | 81.80 |
| NSC638634 | 156.56 |
| NSC56817 | 64.18 |
| NSC79688 | 144.60 |
| NSC18298 | 81.87 |
| NSC115538 | 158.01 |
| NSC71948 | 105.72 |
| NSC22842 | 83.55 |
| NSC175296 | 156.15 |
| NSC33006 | 87.34 |
| NSC80396 | 91.92 |
| NSC82116 | 117.98 |
| NSC158011 | 148.53 |
| NSC22225 | 96.82 |
| NSC37031 | 332.98 |
