# Supplementary material for: Novel lactate dehydrogenase inhibitors with in vivo efficacy against Cryptosporidium parvum
Source: PLoS Pathog. 2019 Jul 29;15(7):e1007953. doi: 10.1371/journal.ppat.1007953 (PMC6687188; doi:10.1371/journal.ppat.1007953)
Supplement: S4 Table — (DOCX) [file ppat.1007953.s004.docx]

**S4 Table. CpLDH inhibition (%) values for Mechanistic Set IV compounds.**

| **COMPOUND NSC NUMBER** | **MEAN CpLDH INHIBITION (%)** |
| --- | --- |
| 1026 | -29.26 |
| 295156 | -1.00 |
| 755 | -5.20 |
| 614928 | -7.87 |
| 51148 | 64.58 |
| 369317 | -8.49 |
| 176324 | -9.23 |
| 63446 | -4.16 |
| 357683 | -9.72 |
| 693053 | -6.53 |
| 3970 | -33.30 |
| 118976 | -18.30 |
| 11779 | -22.15 |
| 695218 | -28.71 |
| 54297 | -31.48 |
| 369318 | -37.61 |
| 620050 | -37.27 |
| 107415 | -39.15 |
| 606532 | -39.53 |
| 1771 | 13.35 |
| 4728 | -23.83 |
| 261726 | -40.28 |
| 18804 | -36.91 |
| 3852 | -37.64 |
| 99733 | -38.11 |
| 625639 | -116.71 |
| 625748 | -117.70 |
| 128734 | -52.88 |
| 403883 | -51.63 |
| 3905 | -48.97 |
| 4960 | 0 |
| 285166 | -48.97 |
| 18805 | -40.28 |
| 4170 | -36.91 |
| 111041 | -37.64 |
| 61805 | -38.11 |
| 664298 | -116.71 |
| 132493 | -117.70 |
| 635441 | -52.88 |
| 5200 | -51.63 |
| 5354 | 0 |
| 529469 | -40.28 |
| 37364 | -36.91 |
| 4857 | -37.64 |
| 163501 | -38.10 |
| 95580 | -114.38 |
| 673912 | -116.36 |
| 159935 | -51.31 |
| 635975 | -52.75 |
| 7210 | -48.97 |
| 65381 | 0 |
| 626433 | 48.62 |
| 349438 | 13.12 |
| 11897 | -2.25 |
| 253272 | -2.87 |
| 95678 | -37.43 |
| 1620 | -4.87 |
| 166464 | -7.16 |
| 657449 | -13.90 |
| 7833 | -11.00 |
| 89303 | -7.26 |
| 635968 | -7.14 |
| 400944 | -2.52 |
| 40212 | -35.86 |
| 326397 | -5.75 |
| 98447 | 0.02 |
| 4280 | -27.03 |
| 296961 | -16.86 |
| 666526 | -2.48 |
| 11905 | -39.26 |
| 174280 | -5.75 |
| 749 | -18.77 |
| 603719 | -2.52 |
| 45388 | -35.86 |
| 347466 | -3.84 |
| 148958 | -3.43 |
| 26045 | -41.99 |
| 326231 | -24.75 |
| 688795 | -40.28 |
| 18938 | -83.85 |
| 21548 | -0.10 |
| 126849 | -22.36 |
| 375575 | -28.69 |
| 664286 | -26.92 |
| 10447 | 39.07 |
| 65423 | -32.77 |
| 146604 | -38.75 |
| 283162 | -65.77 |
| 400978 | -79.24 |
| 667235 | -25.51 |
| 33004 | -8.80 |
| 169543 | -46.26 |
| 379531 | -50.91 |
| 664331 | -15.78 |
| 13966 | -21.73 |
| 80087 | -24.95 |
| 175274 | -25.99 |
| 286193 | -26.38 |
| 405158 | -30.77 |
| 667251 | -17.96 |
| 56544 | -23.78 |
| 175634 | -31.59 |
| 602617 | -40.33 |
| 674495 | -21.24 |
| 15200 | -34.76 |
| 85236 | -30.32 |
| 176655 | -23.23 |
| 292147 | -13.84 |
| 634471 | -23.23 |
| 676561 | -16.89 |
| 66914 | -32.10 |
| 299879 | -32.05 |
| 607347 | -68.99 |
| 697726 | -69.94 |
| 22194 | -50.61 |
| 85998 | -57.91 |
| 191384 | -34.30 |
| 307454 | -54.21 |
| 634650 | -31.23 |
| 7364 | -53.68 |
| 86100 | -9.40 |
| 319726 | -58.85 |
| 625355 | -50.28 |
| 186 | -47.49 |
| 29603 | -54.90 |
| 105808 | -67.00 |
| 191389 | -64.07 |
| 310618 | -54.97 |
| 637729 | -66.55 |
| 9856 | -25.71 |
| 87206 | -25.74 |
| 328587 | -53.59 |
| 635563 | -59.68 |
| 750 | -36.33 |
| 32946 | -50.60 |
| 119686 | -46.09 |
| 263500 | -58.44 |
| 320846 | -61.75 |
| 643028 | -52.45 |
| 9706 | -62.78 |
| 99027 | -71.89 |
| 373853 | -15.72 |
| 637833 | -64.71 |
| 1906 | -58.60 |
| 54650 | -73.33 |
| 145150 | -64.31 |
| 267213 | -67.55 |
| 336628 | -66.49 |
| 643031 | -51.68 |
| 659501 | -27.66 |
| 118742 | -28.34 |
| 375294 | -39.63 |
| 645987 | -40.48 |
| 4114 | -42.22 |
| 63984 | -45.68 |
| 146268 | -43.94 |
| 278619 | -43.32 |
| 382007 | -43.42 |
| 663996 | -51.63 |
| 106296 | -44.71 |
| 13973 | -3.43 |
| 69187 | -16.81 |
| 89671 | -22.45 |
| 154754 | -36.36 |
| 208914 | -37.53 |
| 605756 | -35.77 |
| 635404 | -38.57 |
| 643910 | -38.49 |
| 31702 | -41.86 |
| 119875 | -45.38 |
| 19994 | -14.24 |
| 70422 | -12.00 |
| 97703 | -3.23 |
| 155595 | -11.00 |
| 264880 | -2.01 |
| 621889 | -0.31 |
| 636817 | -5.00 |
| 654705 | -2.14 |
| 36826 | -1.63 |
| 168221 | -24.94 |
| 22842 | 3.48 |
| 71851 | -30.23 |
| 102811 | -35.09 |
| 165897 | -41.74 |
| 269142 | -41.10 |
| 622627 | -26.97 |
| 637731 | -17.56 |
| 655255 | -35.82 |
| 39202 | -37.56 |
| 175296 | 0.00 |
| 22992 | -3.61 |
| 72961 | -2.40 |
| 105014 | -16.58 |
| 169779 | -13.00 |
| 284356 | -16.93 |
| 622640 | -12.99 |
| 639754 | -8.79 |
| 658144 | -3.51 |
| 41809 | -14.35 |
| 224117 | -0.81 |
| 33006 | 13.74 |
| 74420 | -7.03 |
| 126771 | -15.17 |
| 175493 | -0.88 |
| 308847 | -2.34 |
| 622684 | -0.38 |
| 640391 | -1.88 |
| 687667 | -11.60 |
| 45575 | -10.08 |
| 233872 | -35.08 |
| 35866 | -7.39 |
| 76747 | -6.97 |
| 129943 | -7.55 |
| 185065 | -8.17 |
| 404241 | -6.48 |
| 625641 | -6.68 |
| 640624 | -5.57 |
| 693632 | 0.06 |
| 63701 | -0.31 |
| 267033 | -8.12 |
| 49660 | -3.53 |
| 80396 | -8.51 |
| 139109 | -11.32 |
| 191392 | -18.93 |
| 406021 | -17.89 |
| 627708 | -13.84 |
| 643174 | -15.39 |
| 697923 | -17.27 |
| 79456 | -17.92 |
| 292684 | -11.50 |
| 63878 | -5.88 |
| 82116 | 10.05 |
| 145669 | 0.07 |
| 191393 | -10.83 |
| 408120 | -6.79 |
| 632233 | -7.14 |
| 643186 | -12.83 |
| 12825 | -13.98 |
| 95848 | -17.43 |
| 349155 | -5.62 |
| 407010 | -2.40 |
| 631583 | -36.52 |
| 650573 | -21.28 |
| 38721 | -27.12 |
| 104801 | -32.70 |
| 208913 | -30.10 |
| 616232 | -42.37 |
| 631152 | -45.89 |
| 1027 | -2.97 |
| 634224 | -37.07 |
| 407335 | -11.80 |
| 634863 | -18.12 |
| 652287 | -15.92 |
| 59269 | 0.04 |
| 106995 | -5.05 |
| 224131 | -4.77 |
| 618332 | -7.70 |
| 631160 | -15.11 |
| 18891 | -7.99 |
| 666168 | -17.65 |
| 610744 | -19.53 |
| 635140 | -15.60 |
| 658709 | -17.00 |
| 65346 | -11.87 |
| 128305 | -10.13 |
| 266535 | -18.25 |
| 619165 | -14.52 |
| 633001 | -15.73 |
| 36693 | -16.69 |
| 118732 | -7.91 |
| 611750 | -11.25 |
| 636126 | -16.52 |
| 659997 | -9.35 |
| 68093 | -17.03 |
| 147340 | -13.68 |
| 267461 | -44.20 |
| 620277 | -46.45 |
| 637914 | -47.07 |
| 47147 | -37.67 |
| 4810 | -33.40 |
| 622616 | -39.56 |
| 636132 | -58.66 |
| 662553 | -64.36 |
| 78365 | -60.70 |
| 154020 | -38.91 |
| 328477 | -31.70 |
| 622732 | -35.66 |
| 664327 | -14.71 |
| 65937 | -25.00 |
| 28002 | -26.04 |
| 625487 | -32.18 |
| 636786 | -25.27 |
| 680516 | -19.55 |
| 79451 | -40.98 |
| 168415 | -49.77 |
| 329279 | -39.07 |
| 625483 | -18.24 |
| 667467 | -21.66 |
| 80756 | -22.71 |
| 60309 | -23.17 |
| 629301 | -23.71 |
| 643175 | -25.23 |
| 25149 | -16.60 |
| 82025 | -16.46 |
| 175636 | -17.73 |
| 330770 | -9.40 |
| 625590 | -11.59 |
| 680506 | -8.88 |
| 93739 | -24.21 |
| 338259 | -17.29 |
| 630374 | -20.24 |
| 643774 | -24.42 |
| 34757 | -24.18 |
| 92510 | -23.70 |
| 202000 | -23.71 |
| 352890 | -27.24 |
| 629659 | -32.05 |
| 682864 | -37.11 |
| 106408 | -29.99 |
| 651079 | -28.42 |
| 118735 | -15.17 |
| 299187 | -23.00 |
| 360861 | -29.11 |
| 624161 | -27.85 |
| 635337 | -28.29 |
| 658388 | -30.45 |
| 61811 | -26.07 |
| 241509 | -30.83 |
| 383468 | -30.29 |
| 637680 | -16.33 |
| 140911 | -0.44 |
| 305782 | -11.03 |
| 376791 | -26.30 |
| 624358 | -23.30 |
| 636084 | -20.56 |
| 664329 | -28.29 |
| 99016 | -25.29 |
| 257473 | -76.90 |
| 600305 | -31.37 |
| 642649 | -27.23 |
| 204985 | -20.51 |
| 321803 | -29.51 |
| 603108 | -29.93 |
| 632536 | -35.42 |
| 637578 | -28.84 |
| 5890 | -32.64 |
| 123390 | -31.27 |
| 313981 | -27.27 |
| 616355 | -24.91 |
| 643162 | -31.17 |
| 224124 | -19.59 |
| 335142 | -27.72 |
| 621094 | -33.52 |
| 632839 | -15.78 |
| 643164 | -21.20 |
| 11926 | -19.30 |
| 135996 | -24.95 |
| 316157 | -4.71 |
| 620358 | -24.86 |
| 643163 | -30.02 |
| 235082 | -28.09 |
| 337612 | -26.65 |
| 622608 | -22.44 |
| 632841 | -21.11 |
| 645617 | -28.16 |
| 20514 | -30.20 |
| 143648 | -33.40 |
| 320864 | -26.08 |
| 631529 | -18.60 |
| 646189 | -21.95 |
| 282752 | -63.64 |
| 338720 | 0.02 |
| 622690 | -0.50 |
| 634658 | -26.76 |
| 648422 | -69.44 |
| 26040 | -70.84 |
| 157930 | -72.47 |
| 323241 | -23.75 |
| 633209 | -19.92 |
| 646200 | -24.52 |
| 294577 | -16.31 |
| 339004 | -31.52 |
| 623135 | -28.47 |
| 635121 | 0.03 |
| 651080 | -28.90 |
| 44690 | -28.19 |
| 173905 | -39.37 |
| 376265 | -34.26 |
| 634232 | -33.26 |
| 647613 | -29.39 |
| 294961 | -25.63 |
| 349156 | -36.89 |
| 623637 | -23.29 |
| 635306 | -37.53 |
| 658285 | -36.39 |
| 53908 | -32.76 |
| 178249 | -31.06 |
| 382766 | -40.98 |
| 634396 | -17.78 |
| 651084 | -15.88 |
| 668270 | -4.80 |
| 26273 | -4.046 |
| 157389 | -3.33 |
| 284751 | -2.65 |
| 614826 | -3.85 |
| 635448 | -5.65 |
| 757 | -4.41 |
| 146397 | -1.75 |
| 302358 | 0.36 |
| 621486 | -0.42 |
| 671424 | -1.46 |
| 32982 | 0.02 |
| 163088 | 0.46 |
| 302979 | -1.17 |
| 617540 | -6.79 |
| 635542 | -10.72 |
| 14974 | 0.167 |
| 166381 | -6.81 |
| 305884 | -10.93 |
| 634568 | -8.97 |
| 672904 | -0.06 |
| 36437 | -19.06 |
| 174163 | -16.59 |
| 327697 | -11.09 |
| 620279 | -17.13 |
| 640580 | -13.66 |
| 24817 | -20.22 |
| 169600 | -28.27 |
| 322069 | -28.89 |
| 62791 | -22.35 |
| 678932 | -5.91 |
| 83265 | -6.04 |
| 182986 | -5.56 |
| 343513 | -0.97 |
| 626734 | -0.09 |
| 645033 | -3.66 |
| 77021 | -2.42 |
| 172946 | -10.73 |
| 403148 | -8.59 |
| 689872 | -7.48 |
| 684845 | -7.56 |
| 97911 | -11.97 |
| 185056 | -5.23 |
| 352876 | -6.93 |
| 629713 | -5.77 |
| 647363 | -3.74 |
| 98542 | -0.82 |
| 249992 | -1.22 |
| 603577 | -0.38 |
| 157004 | -1.50 |
| 1011 | -0.99 |
| 115538 | 0.63 |
| 211500 | -13.24 |
| 407806 | -16.59 |
| 629971 | -14.81 |
| 681730 | -12.34 |
| 104117 | -18.47 |
| 262665 | -17.00 |
| 293927 | -15.46 |
| 329277 | -1.09 |
| 14574 | -14.41 |
| 123115 | -0.99 |
| 241906 | -0.38 |
| 601101 | -6.25 |
| 631521 | -0.12 |
| 681741 | -7.66 |
| 126727 | -5.88 |
| 268986 | -15.01 |
| 69852 | -11.66 |
| 163443 | -14.23 |
| 24113 | -11.52 |
| 140377 | -8.72 |
| 265459 | -11.78 |
| 604535 | -8.81 |
| 635437 | -7.08 |
| 377 | -0.53 |
| 136037 | -0.26 |
| 281245 | -8.53 |
| 314622 | -4.47 |
| 371846 | -16.04 |
| 624169 | -25.23 |
| 657456 | -18.36 |
| 2979 | -29.91 |
| 33410 | -43.24 |
| 51812 | -26.06 |
| 84074 | -40.93 |
| 138429 | -38.75 |
| 196524 | -30.88 |
| 293015 | -3.81 |
| 351306 | -39.05 |
| 634473 | -29.85 |
| 678917 | -41.85 |
| 4644 | -25.29 |
| 35489 | -39.94 |
| 56737 | -38.88 |
| 85561 | 73.45 |
| 142982 | -29.74 |
| 211489 | -11.02 |
| 311153 | -30.69 |
| 374898 | -39.33 |
| 635321 | -39.51 |
| 680509 | -41.56 |
| 11930 | -34.92 |
| 35949 | -34.03 |
| 66300 | -42.12 |
| 85700 | -37.65 |
| 149765 | -43.20 |
| 256927 | -46.96 |
| 329696 | -30.73 |
| 600300 | -49.73 |
| 635326 | -38.78 |
| 684480 | -22.58 |
| 14229 | -42.97 |
| 39863 | -20.27 |
| 67580 | -41.90 |
| 98904 | -42.18 |
| 167410 | -0.52 |
| 265473 | -39.94 |
| 330515 | -43.86 |
| 603578 | -56.74 |
| 635328 | -47.14 |
| 705330 | -49.87 |
| 19857 | -57.96 |
| 40666 | -57.18 |
| 67690 | -56.08 |
| 100856 | -59.57 |
| 168597 | -54.94 |
| 267700 | -49.53 |
| 331757 | -61.61 |
| 618261 | -65.69 |
| 635435 | -63.48 |
| 534 | -63.87 |
| 24048 | -65.48 |
| 43321 | -58.94 |
| 71300 | -64.35 |
| 104129 | -53.74 |
| 170984 | -56.20 |
| 267712 | -57.63 |
| 337766 | -53.76 |
| 620280 | -51.47 |
| 635438 | -43.53 |
| 740 | -51.72 |
| 24818 | -40.47 |
| 47438 | -57.83 |
| 71669 | -53.065 |
| 109444 | -58.72 |
| 173904 | -56.33 |
| 273829 | -39.73 |
| 345081 | -64.34 |
| 623746 | -45.42 |
| 644735 | -56.47 |
| 2186 | -64.59 |
| 24819 | -66.99 |
| 48151 | -66.17 |
| 73413 | 54.98 |
| 113090 | -44.20 |
| 174176 | -60.94 |
| 292663 | -63.04 |
| 349644 | -64.47 |
| 624158 | -8.09 |
| 624947 | -0.76 |
| 635366 | -12.08 |
| 641253 | -12.80 |
| 657446 | -8.01 |
| 668260 | -9.25 |
| 689857 | -0.01 |
| 32992 | -5.35 |
| 96932 | -8.62 |
| 15623 | -7.71 |
| 643148 | -12.75 |
| 626120 | -0.60 |
| 635436 | -79.39 |
| 641607 | -85.63 |
| 657598 | -68.44 |
| 670224 | -73.95 |
| 693172 | -0.60 |
| 34391 | -25.08 |
| 102815 | -3.42 |
| 40341 | -22.69 |
| 10010 | -19.54 |
| 627666 | -0.33 |
| 635824 | -3.02 |
| 642048 | -4.29 |
| 657603 | -45.82 |
| 670225 | -54.38 |
| 697443 | -57.12 |
| 36354 | -56.18 |
| 90487 | -42.73 |
| 164909 | -58.07 |
| 623051 | -30.82 |
| 629738 | -0.22 |
| 635833 | -26.59 |
| 643599 | -33.57 |
| 658293 | -21.86 |
| 670226 | -20.67 |
| 698031 | -6.31 |
| 45383 | -9.24 |
| 106997 | -28.98 |
| 183359 | -29.84 |
| 623059 | -31.35 |
| 634503 | -5.79 |
| 637916 | -14.05 |
| 647418 | -56.81 |
| 658494 | -56.68 |
| 670229 | -52.64 |
| 7521 | -52.65 |
| 49451 | -57.10 |
| 622586 | -55.77 |
| 282880 | -0.05 |
| 90829 | -2.93 |
| 634926 | -0.08 |
| 638634 | 0.30 |
| 648419 | -12.60 |
| 659174 | -13.23 |
| 673622 | -14.54 |
| 7522 | -4.34 |
| 56817 | 37.75 |
| 624206 | -15.13 |
| 376248 | -15.03 |
| 138925 | -14.37 |
| 634928 | -21.25 |
| 639828 | -42.32 |
| 650792 | -46.61 |
| 662825 | -7.35 |
| 682769 | -0.10 |
| 14975 | -3.61 |
| 79688 | 40.15 |
| 640974 | -7.97 |
| 605583 | -33.98 |
| 622589 | -7.95 |
| 635312 | -41.63 |
| 641228 | -51.30 |
| 657298 | -42.34 |
| 664181 | -5.59 |
| 689228 | -5.60 |
| 18298 | 53.86 |
| 93419 | -51.14 |
| 672425 | -0.60 |
| 637993 | -46.33 |
| 643351 | -4.10 |
| 118030 | -56.74 |
| 167780 | -51.52 |
| 184403 | -44.28 |
| 269754 | -47.56 |
| 330516 | -37.93 |
| 617570 | -41.22 |
| 640985 | -66.15 |
| 653000 | -58.42 |
| 670140 | -63.78 |
| 690634 | -61.23 |
| 126728 | -73.96 |
| 169676 | -69.84 |
| 240419 | -69.26 |
| 285223 | -56.39 |
| 330753 | -63.01 |
| 623093 | -49.57 |
| 641233 | -51.96 |
| 654259 | -46.44 |
| 671136 | -17.97 |
| 699479 | -31.87 |
| 129414 | -16.26 |
| 172924 | -39.50 |
| 243928 | -32.64 |
| 288010 | -45.22 |
| 345647 | -176.19 |
| 623095 | -151.18 |
| 641240 | -190.19 |
| 657457 | -150.87 |
| 671394 | -158.34 |
| 703550 | -165.27 |
| 132791 | -161.16 |
| 174121 | -134.26 |
| 245432 | -45.30 |
| 290205 | -205.36 |
| 354844 | -68.14 |
| 635544 | -233.40 |
| 641245 | -19.61 |
| 657722 | -170.05 |
| 679524 | -184.78 |
| 5159 | -38.07 |
| 133071 | -57.79 |
| 177365 | -212.36 |
| 255109 | -205.22 |
| 305222 | -217.96 |
| 361813 | -202.91 |
| 638646 | -17.60 |
| 641250 | -16.86 |
| 657799 | 26.14 |
| 679527 | -27.66 |
| 7525 | -34.67 |
| 139105 | -28.54 |
| 180973 | -19.27 |
| 258812 | -2.78 |
| 305819 | -15.30 |
| 363744 | -9.54 |
| 640584 | -6.68 |
| 642033 | -12.26 |
| 658139 | -4.51 |
| 681744 | -15.26 |
| 7530 | -3.24 |
| 164914 | -26.61 |
| 181486 | -5.18 |
| 268251 | -8.04 |
| 328166 | -4.71 |
| 363998 | -2.96 |
| 640637 | -17.02 |
| 642040 | -9.38 |
| 658350 | -19.59 |
| 686349 | 31.74 |
| 19990 | -27.23 |
| 165563 | -22.46 |
| 184398 | -31.06 |
| 269148 | -25.91 |
| 330500 | -19.04 |
| 603624 | -12.73 |
| 640638 | -14.60 |
| 644794 | -17.50 |
| 659999 | -30.12 |
| 687330 | -17.98 |
| 30916 | -5.35 |
| 46061 | -107.66 |
| 97338 | -120.23 |
| 169774 | -107.83 |
| 281613 | -15.80 |
| 607316 | -7.22 |
| 653558 | -17.98 |
| 67574 | -20.16 |
| 306864 | -7.27 |
| 154890 | -11.41 |
| 292567 | -134.14 |
| 52141 | -100.84 |
| 103248 | -105.50 |
| 208734 | -140.20 |
| 304421 | -126.27 |
| 619907 | -115.80 |
| 669356 | -120.43 |
| 76027 | -85.04 |
| 325319 | -133.91 |
| 173046 | -141.42 |
| 675593 | -141.86 |
| 65104 | -113.50 |
| 109350 | -1.18 |
| 218439 | -11.45 |
| 328426 | -30.81 |
| 620261 | -132.95 |
| 676963 | -97.54 |
| 90636 | -76.58 |
| 363182 | -79.07 |
| 219734 | -99.53 |
| 677392 | -119.10 |
| 68075 | -148.49 |
| 116693 | -151.85 |
| 243023 | -140.35 |
| 332598 | -143.77 |
| 624953 | -142.25 |
| 7532 | -151.91 |
| 107412 | -178.36 |
| 603169 | -164.45 |
| 267229 | -179.02 |
| 166454 | -139.75 |
| 70845 | -114.16 |
| 136044 | -159.43 |
| 248436 | -25.94 |
| 337851 | -16.37 |
| 625331 | -26.39 |
| 24559 | -84.22 |
| 115493 | -100.16 |
| 638352 | 43.74 |
| 683792 | -73.55 |
| 192965 | -81.94 |
| 73495 | -99.50 |
| 145366 | -111.71 |
| 265450 | -83.48 |
| 353527 | -42.92 |
| 625873 | -25.33 |
| 34931 | 43.74 |
| 226080 | -83.38 |
| 640342 | -89.53 |
| 700582 | -83.75 |
| 260610 | -78.82 |
| 76455 | -30.19 |
| 153858 | -47.87 |
| 268242 | -40.28 |
| 359463 | -17.14 |
| 640335 | -79.51 |
| 49842 | -79.52 |
| 253995 | 25.71 |
| 139490 | -2.06 |
| 70929 | 13.50 |
| 276299 | -25.02 |
| 93135 | -27.33 |
| 156215 | -35.58 |
| 274893 | -23.27 |
| 600681 | -36.35 |
| 645567 | -28.84 |
| 58514 | -30.14 |
| 269146 | -38.98 |
| 324368 | -32.50 |
| 265211 | -47.33 |
| 239375 | -42.34 |
| 697468 | -5.22 |
| 622116 | -10.74 |
| 3053 | -3.83 |
| 622124 | 0.01 |
| 18268 | -2.01 |
| 643834 | -1.59 |
| 32065 | -2.35 |
| 615593 | -2.09 |
| 71948 | 60.47 |
| 125066 | -9.07 |
| 125176 | -0.47 |
| 285116 | -2.26 |
| 333856 | -2.71 |
| 70925 | 68.17 |
