# Supplementary figures and images for: Novel lactate dehydrogenase inhibitors with in vivo efficacy against Cryptosporidium parvum
Source: PLoS Pathog. 2019 Jul 29;15(7):e1007953. doi: 10.1371/journal.ppat.1007953 (PMC6687188; doi:10.1371/journal.ppat.1007953)

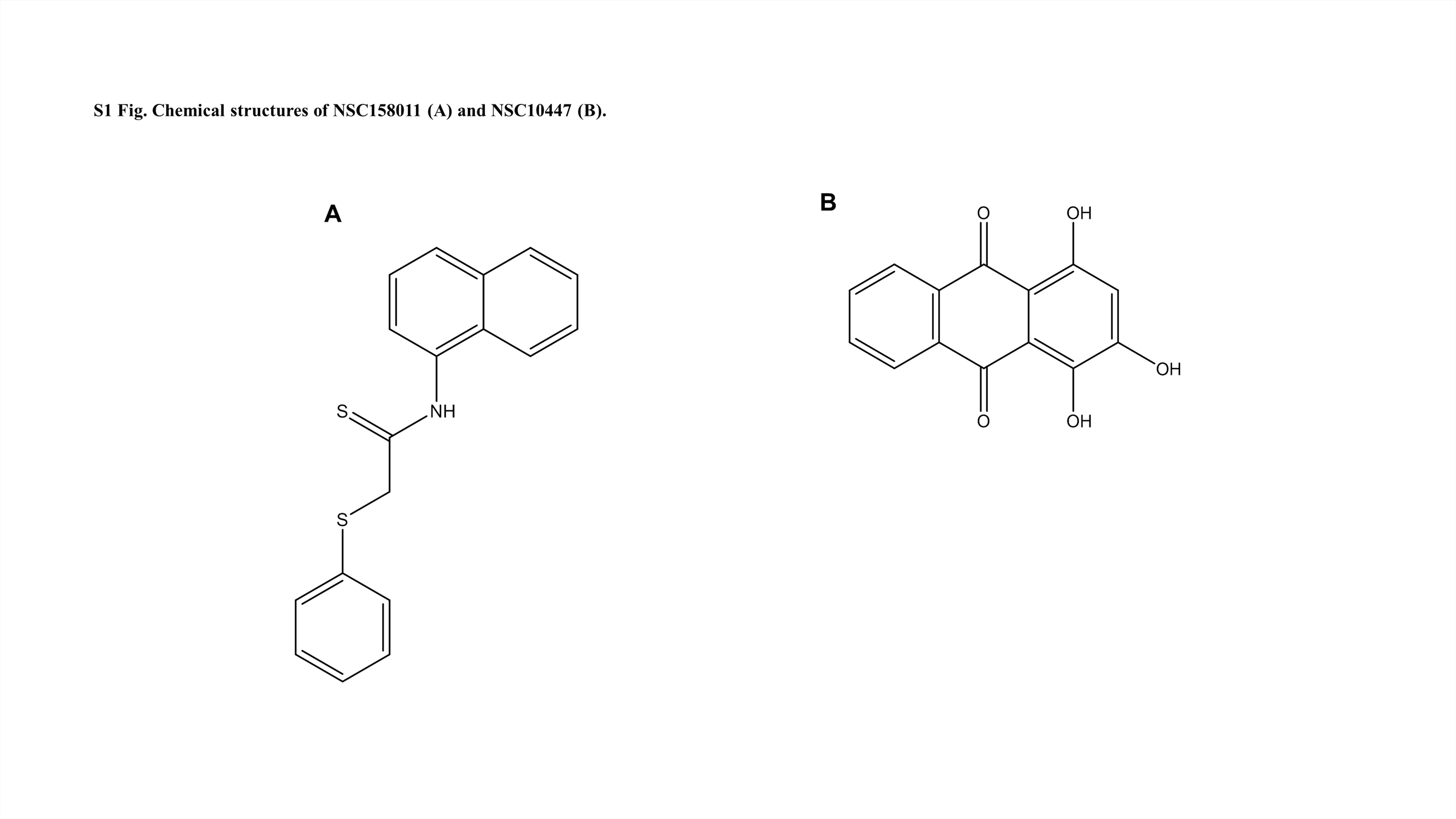

Supplement: S1 Fig — Chemical structures of NSC158011 (A) and NSC10447 (B). (TIF) [file ppat.1007953.s005.tif]

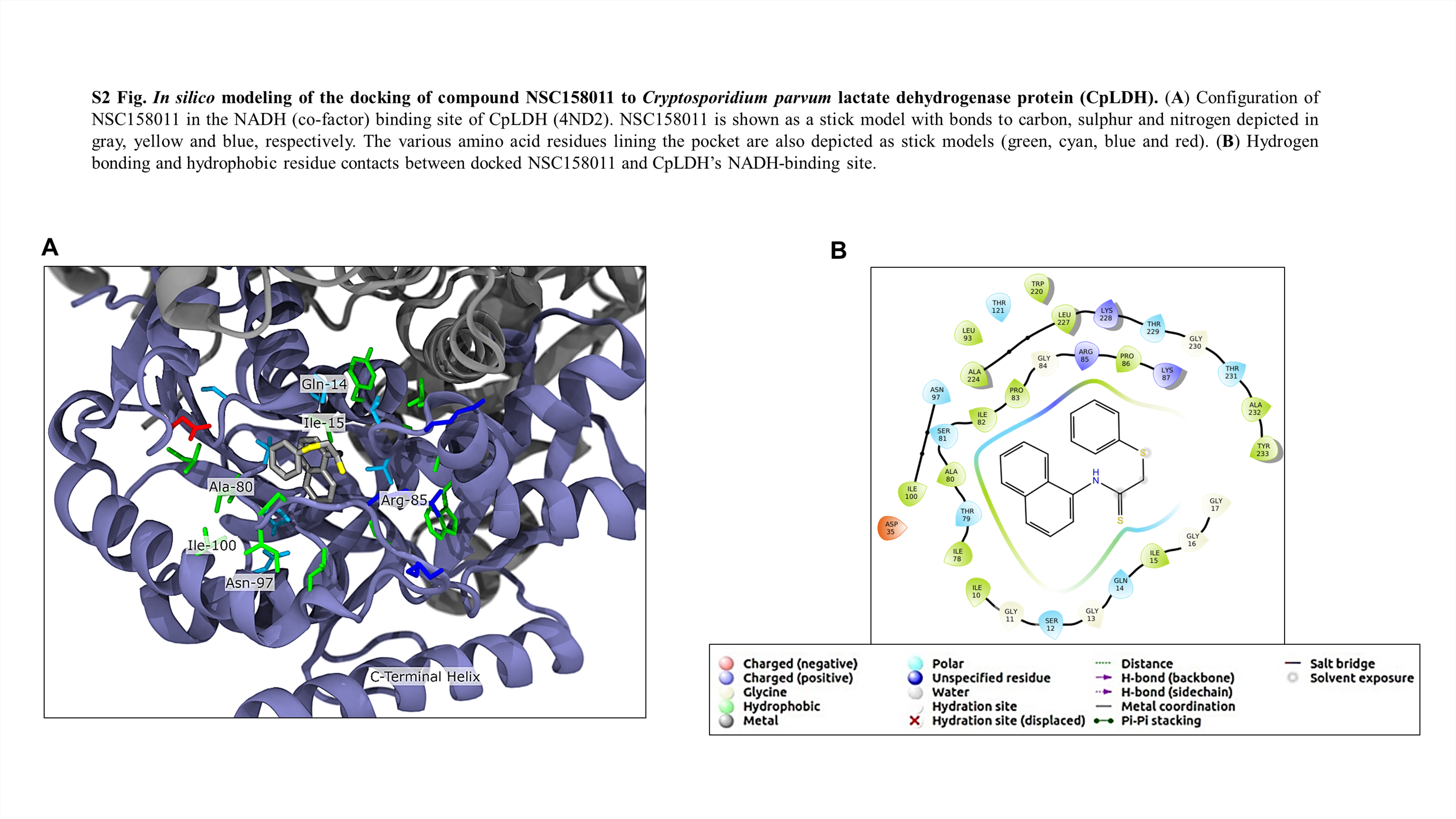

Supplement: S2 Fig — (A) Configuration of NSC158011 in the NADH (co-factor) binding site of CpLDH (4ND2). NSC158011 is shown as a stick model with bonds to carbon, Sulphur and nitrogen depicted in gray, yellow and blue, respectively. The various amino acid residues lining the pocket are also depicted as stick models (green, cyan, blue and red). (B) Hydrogen bonding and hydrophobic residue contacts between docked NSC158011 and CpLDH’s NADH-binding site. (TIF) [file ppat.1007953.s006.tif]

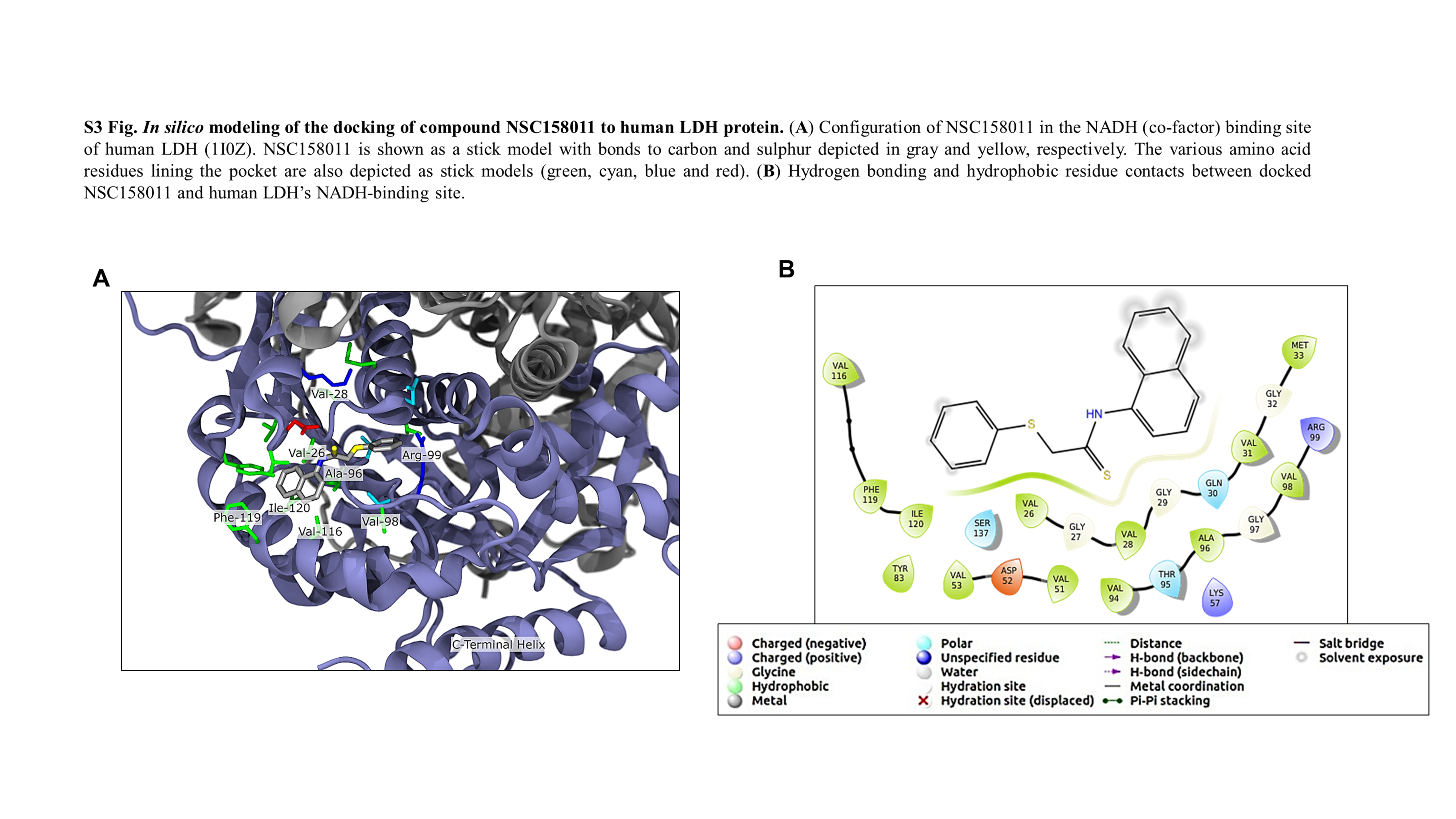

Supplement: S3 Fig — (A) Configuration of NSC158011 in the NADH (co-factor) binding site of human LDH (1I0Z). NSC158011 is shown as a stick model with bonds to carbon and sulphur depicted in gray and yellow, respectively. The various amino acid residues lining the pocket are also depicted as stick models (green, cyan, blue and red). (B) Hydrogen bonding and hydrophobic residue contacts between docked NSC158011 and human LDH’s NADH-binding site. (TIF) [file ppat.1007953.s007.tif]

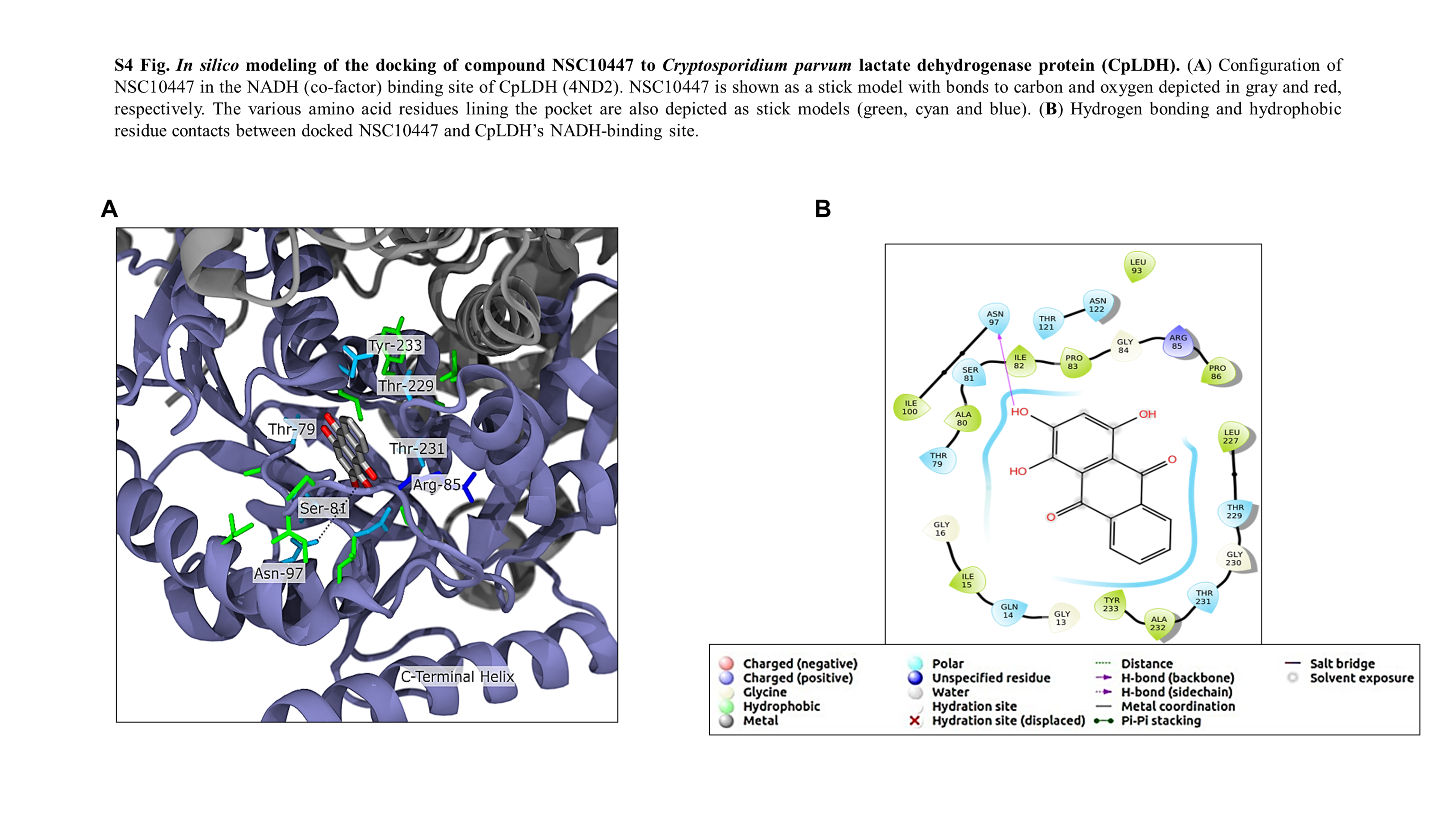

Supplement: S4 Fig — (A) Configuration of NSC10447 in the NADH (co-factor) binding site of CpLDH (4ND2). NSC10447 is shown as a stick model with bonds to carbon and oxygen depicted in gray and red, respectively. The various amino acid residues lining the pocket are also depicted as stick models (green, cyan and blue). (B) Hydrogen bonding and hydrophobic residue contacts between docked NSC10447 and CpLDH’s NADH-binding site. (TIF) [file ppat.1007953.s008.tif]

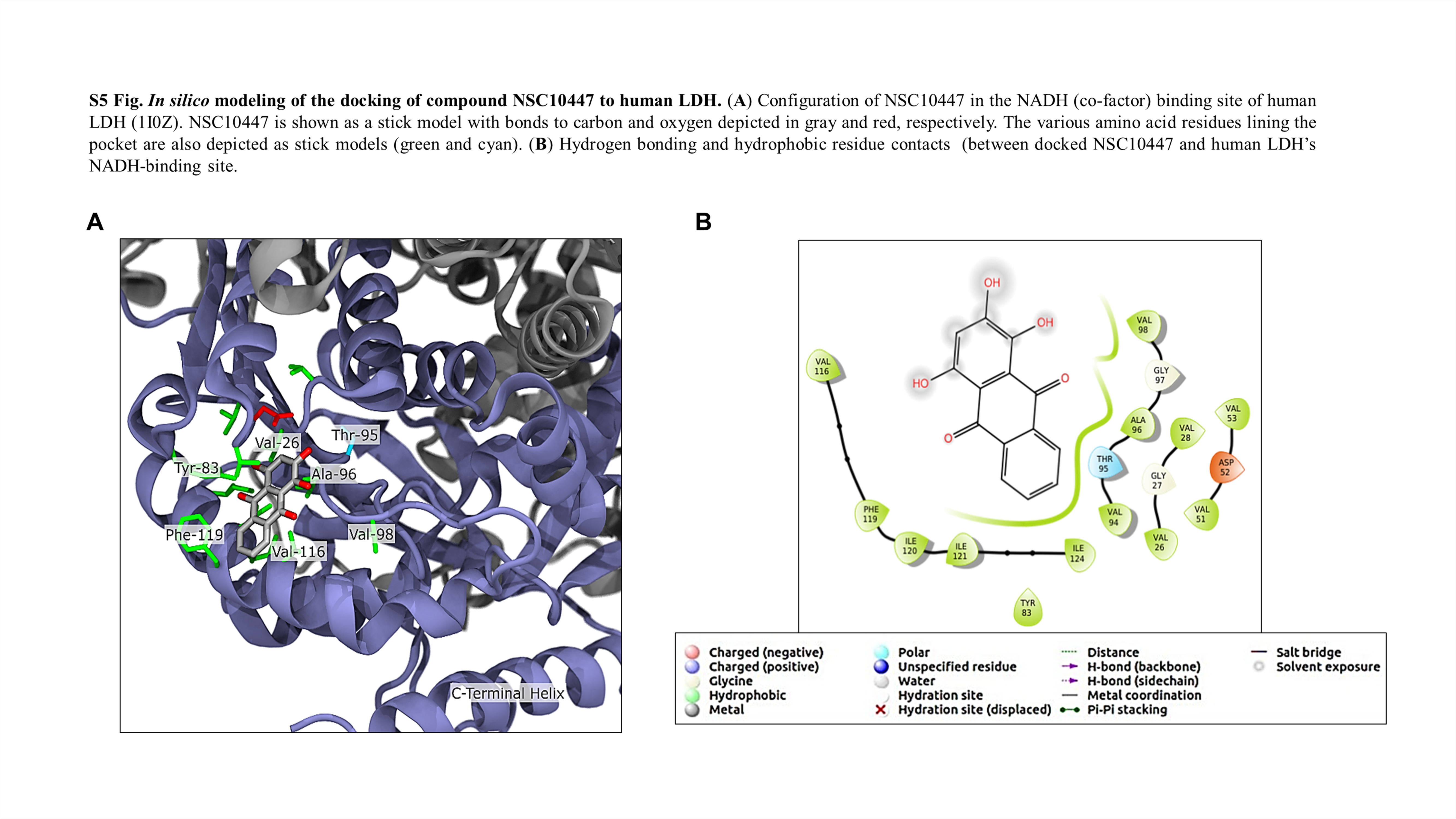

Supplement: S5 Fig — (A) Configuration of NSC10447 in the NADH (co-factor) binding site of human LDH (1I0Z). NSC10447 is shown as a stick model with bonds to carbon and oxygen depicted in gray and red, respectively. The various amino acid residues lining the pocket are also depicted as stick models (green and cyan). (B) Hydrogen bonding and hydrophobic residue contacts (between docked NSC10447 and human LDH’s NADH-binding site. (TIF) [file ppat.1007953.s009.tif]
